# Deep ensemble learning enables highly accurate classification of stored red blood cell morphology

**DOI:** 10.1038/s41598-023-30214-w

**Published:** 2023-02-23

**Authors:** Austin H. Routt, Natalia Yang, Nathaniel Z. Piety, Madeleine Lu, Sergey S. Shevkoplyas

**Affiliations:** Department of Biomedical Engineering, Cullen College of Engineering, University of Houston, 3605 Cullen Blvd, Houston, TX 77204-5060 USA

**Keywords:** Biomedical engineering, Health care, Medical research

## Abstract

Changes in red blood cell (RBC) morphology distribution have emerged as a quantitative biomarker for the degradation of RBC functional properties during hypothermic storage. Previously published automated methods for classifying the morphology of stored RBCs often had insufficient accuracy and relied on proprietary code and datasets, making them difficult to use in many research and clinical applications. Here we describe the development and validation of a highly accurate open-source RBC morphology classification pipeline based on ensemble deep learning (DL). The DL-enabled pipeline utilized adaptive thresholding or semantic segmentation for RBC identification, a deep ensemble of four convolutional neural networks (CNNs) to classify RBC morphology, and Kalman filtering with Hungarian assignment for tracking changes in the morphology of individual RBCs over time. The ensembled CNNs were trained and evaluated on thousands of individual RBCs from two open-access datasets previously collected to quantify the morphological heterogeneity and washing-induced shape recovery of stored RBCs. Confusion matrices and reliability diagrams demonstrated under-confidence of the constituent models and an accuracy of about 98% for the deep ensemble. Such a high accuracy allowed the CNN ensemble to uncover new insights over our previously published studies. Re-analysis of the datasets yielded much more accurate distributions of the effective diameters of stored RBCs at each stage of morphological degradation (discocyte: 7.821 ± 0.429 µm, echinocyte 1: 7.800 ± 0.581 µm, echinocyte 2: 7.304 ± 0.567 µm, echinocyte 3: 6.433 ± 0.490 µm, sphero-echinocyte: 5.963 ± 0.348 µm, spherocyte: 5.904 ± 0.292 µm, stomatocyte: 7.080 ± 0.522 µm). The effective diameter distributions were significantly different across all morphologies, with considerable effect sizes for non-neighboring classes. A combination of morphology classification with cell tracking enabled the discovery of a relatively rare and previously overlooked shape recovery of some sphero-echinocytes to early-stage echinocytes after washing with 1% human serum albumin solution. Finally, the datasets and code have been made freely available online to enable replication, further improvement, and adaptation of our work for other applications.

## Introduction

An estimated 4–5 million patients are transfused with approximately 13 million units of stored red blood cells (RBCs) in the United States every year^1,2^. Most of these RBC units are separated from other components of whole blood soon after collection, mixed with an anticoagulant-preservative solution, and stored in a refrigerator at 1–6 °C for up to 6 weeks^1^. Biochemical and mechanical properties of RBCs deteriorate during the hypothermic storage at a rate that depends on various factors like processing and storage methods and characteristics of the donor^1,3,4^. A hallmark of this so-called “storage lesion” is the gradual transformation of RBC shape from healthy flexible discocytes through various intermediate stages of echinocytosis to rigid and fragile spherocytes, which are prone to lysis and are rapidly cleared by the spleen when transfused^5–8^.

Recently, RBC morphology has emerged as an integrative marker of the overall functional quality of stored blood^6–10^. The rate of the echinocytic transformation is highly variable among individual RBCs, even within the same unit; by the end of the allowable storage, a unit contains a heterogeneous mixture of RBCs at every stage of the morphological degradation^5,11^. Quantifying the distribution of stored RBCs in a unit over different morphological classes is important because both the overall shape deterioration and the presence of even a small fraction of sphero-echinocytes/spherocytes could have a profound impact on the perfusion of capillary networks^6,8,12^. Evaluation of RBC morphology is a notoriously tedious and error-prone process during which an expert manually observes and classifies the shape of 200 to 1,500 individual RBCs to establish a sample distribution that ostensibly reflects the properties of ~ 2 × 10^12^ RBCs contained in a typical unit^5,11,13^.

To simplify the evaluation of RBC morphology, we have previously developed an automated system that combined an easy-to-use microfluidic device for rapidly acquiring thousands of high-quality images of individual RBCs and a binary decision-tree algorithm for segmenting and classifying the images^5^. The classification accuracy of the algorithm (which mimicked the manual RBC classification process) was only 73%^5^. This level of accuracy proved insufficient for most practical applications, such as comparisons of storage conditions and washing methods^12–14^.

Fortunately, rapid advancements in the field of machine learning have yielded deep convolutional neural network architectures (CNNs) that surpassed all former approaches to image classification tasks^15–19^. Most scientific fields have by now felt the impact of deep learning (DL) enabled image analysis and there have already been several attempts to utilize CNNs for classifying morphology of RBCs in different contexts^20–23^. Although these previous studies showed varying degrees of success, each approach has been tailored to a specific imaging modality, and the common lack of dataset and code accessibility made it impossible to compare the approaches. Furthermore, none of these studies explored CNN ensembling^24^ to reduce variance or implemented tracking to follow the evolution of RBC shape changes through time. Thus, the usefulness of these previous solutions to researchers in the field of blood storage and transfusion medicine remains limited.

Here, we describe the development and validation of a DL-enabled RBC morphology classification pipeline that utilizes adaptive thresholding or semantic segmentation for RBC identification, an ensemble of four pre-trained CNNs for classification of RBC morphology, and Kalman filtering with Hungarian assignment for tracking changes in the morphology of individual RBCs over time. We trained and validated the pipeline on two image datasets collected as part of previously published studies^5,25^. The first dataset, ‘Morphological Heterogeneity’ (MH)^26^, was collected in a study that described the previously mentioned automated system consisting of a microfluidic device for acquiring high-quality images and a decision-tree algorithm for segmenting and classifying the images. To generate the MH dataset, seven units of stored RBCs were sampled after 6, 7, and 8 weeks of hypothermal storage. The samples were passed through the microfluidic device to acquire images of more than a million individual RBCs^5^. The second dataset, ‘Cells-In-Wells’ (CIW)^27^, was collected in a study that investigated the dynamics of shape recovery by stored RBCs after washing with normal saline or a 1% solution of human serum albumin (HSA). To generate the CIW dataset, samples from six RBC units were collected after 4, 5, and 6 weeks of cold storage, loaded into an array of microfluidic wells, and washed by adding a large volume of normal saline or 1% HSA. High-resolution images of the cells in wells were acquired every second for about 17 min, so a human expert could quantify the change in shape during the washing process for thousands of individual RBCs^25^.

We demonstrated the utility of the DL-enabled classification of RBC morphology by (i) processing the entire MH dataset to gain better estimates of central tendency and variability of the effective diameter of RBCs belonging to different morphological classes and (ii) tracking each RBC present in the CIW dataset to discover a rare shape recovery transformation which was deemed impossible in previous studies. Finally, we made both datasets (including the original bright-field microscopy images, binary image masks, bounding boxes, various identifiers, statistics, and labels)^26,27^ and the code (developed in MATLAB, an easy-to-learn computing platform popular in academia and industry alike)^28,29^ available freely online. We anticipate that such open access will maximize the potential usefulness of this study to researchers in the field of blood storage and transfusion medicine and will also benefit those in the broader scientific community interested in benchmarking current and future RBC classification models using the datasets.

## Results and discussion

### Development and validation of the DL-enabled RBC morphology classification pipeline: MH dataset

**Figure 1:**
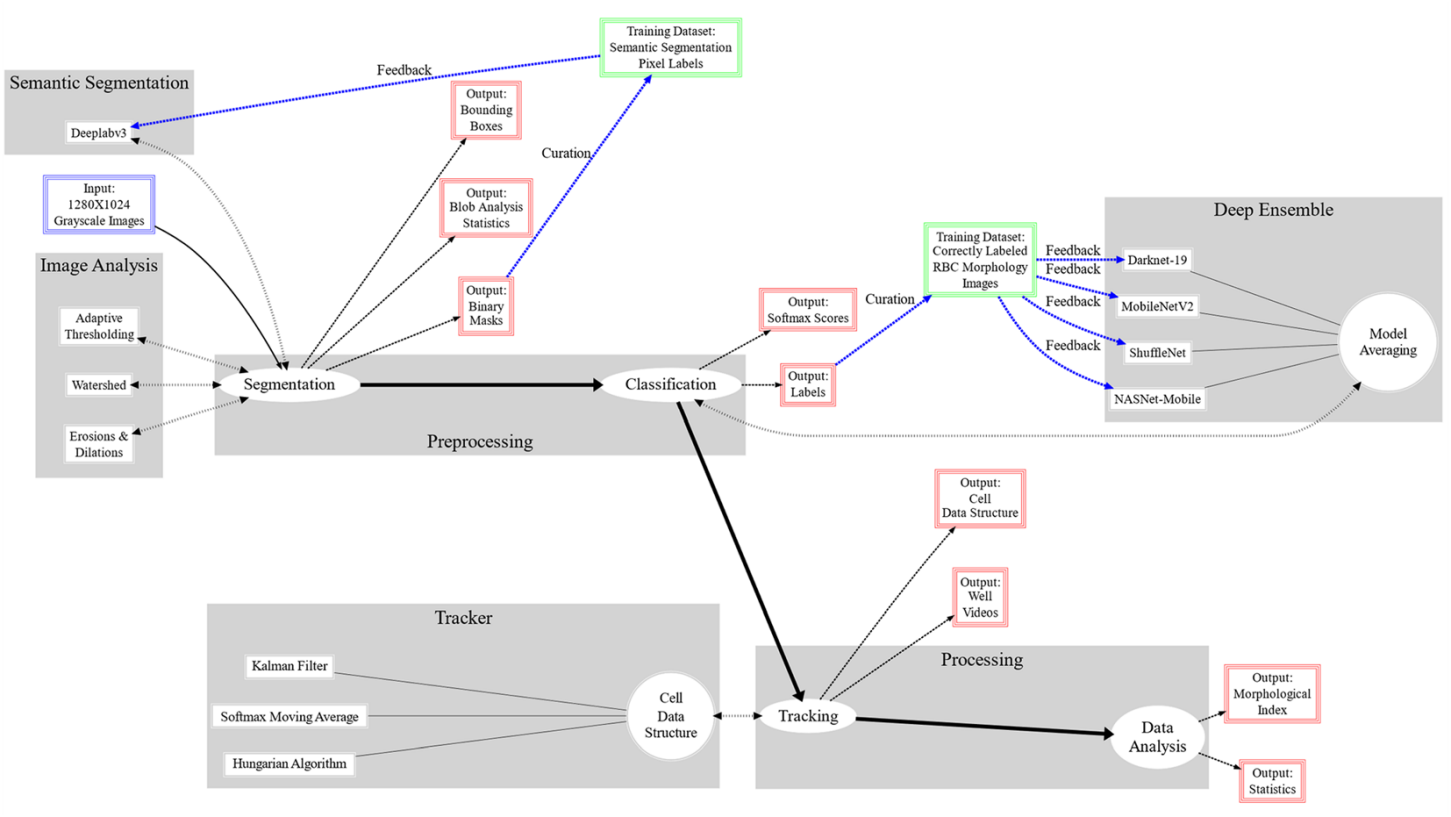
A diagram illustrating the structure and workflow of the RBC image analysis pipeline/framework. Grey boxes are modules composed of subprocesses (white ovals or boxes) that receive and output data. Some outputs (red boxes with blue outgoing arrows) can be used to improve the system with the help of expert curation & feedback (green boxes).

**Figure 2:**
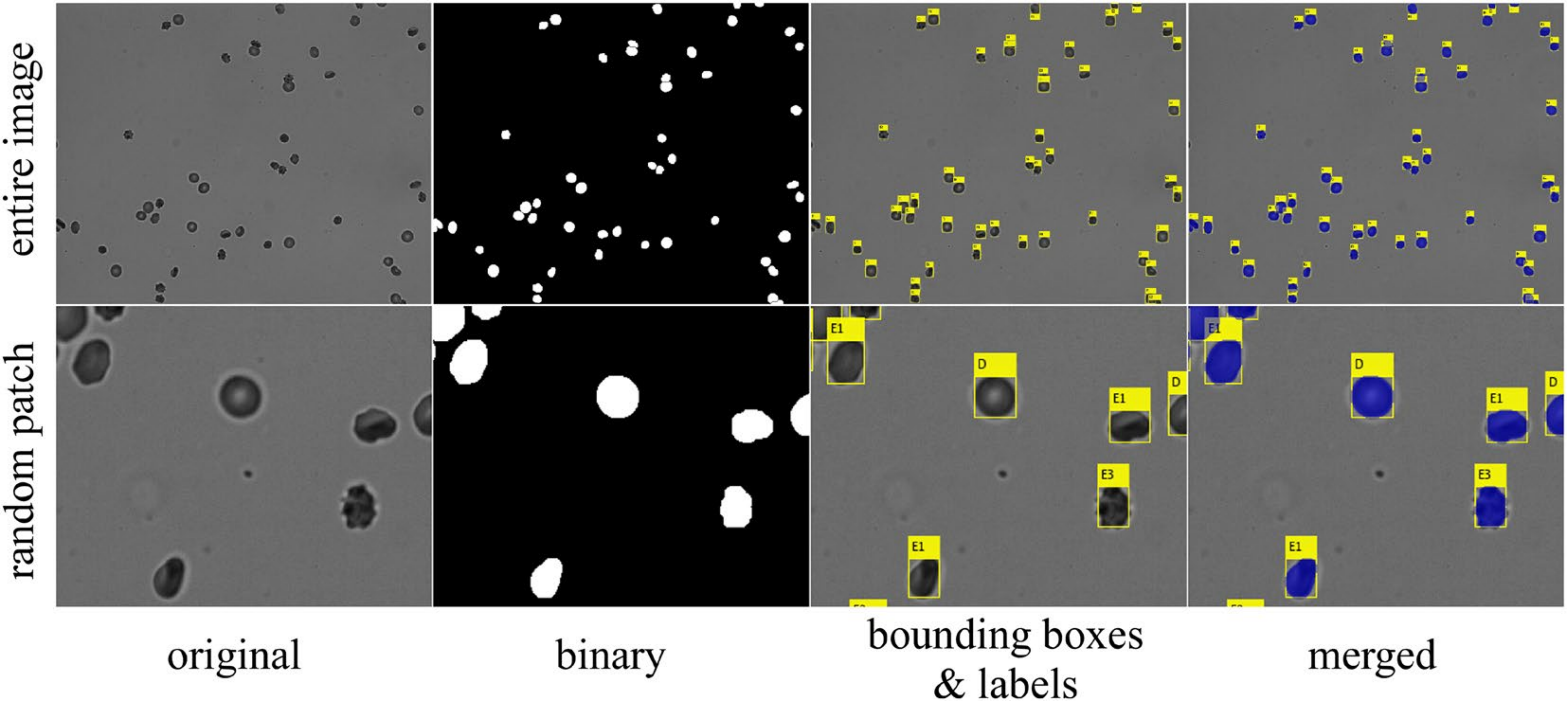
Application of the deep learning (DL) enabled RBC morphology classification to images from the MH dataset. A random image from the MH dataset showing the binary mask, bounding boxes, and RBC labels superimposed on the original image. Labels correspond to one of seven classes: discocyte (D), echinocyte 1 (E1), echinocyte 2 (E2), echinocyte 3 (E3), sphero-echinocyte (SE), spherocyte (S), and stomatocyte (ST).

Figure 1 shows the entire framework of the DL-enabled RBC morphology classification pipeline, composed of modules (e.g., preprocessing, processing, image analysis) that feed data into specific routines (e.g., segmentation, classification, tracking, model averaging). The workflow begins with 1280 × 1024 grayscale images and produces various outputs from each routine (i.e., bounding boxes, labels, statistics, videos). An expert can manually curate lab outputs from images and then feed them back into the system to improve accuracy by increasing the size of the training set. Figure 2 illustrates the application of the morphology classification pipeline to the images from the MH dataset (for additional details see supplementary Fig. S1–S3)^5,26^. Because the original images had relatively clear backgrounds with only slight variations in illumination, we were able to implement a straightforward segmentation method based on adaptive thresholding. The segmentation method used morphological dilations and erosions to remove specks and fill holes, and the watershed algorithm to ensure that touching cells had dividing lines that differentiated their silhouettes. We used the resulting binary mask to perform blob analysis and define RBC bounding boxes, and then passed cropped images of individual RBCs to an ensemble of four CNNs (described below) for the classification of RBC morphology. When properly segmented and cleaned, the MH dataset yielded 1,294,996 individual RBCs (Fig. S4–S12)^26^. A subset of the MH dataset was pre-classified by an expert to create a subset of 13,353 images of individual RBCs classified into seven morphology classes, including discocytes (D), echinocytes 1 (E1), echinocytes 2 (E2), echinocytes 3 (E3), sphero-echinocytes (SE), spherocytes (S), and stomatocytes (ST). This pre-classified set was split into an MH training set (90%) to train the CNNs used in this study and an MH test set (10% holdout) to test the classification accuracy of the trained CNN ensemble.

Classification of RBC morphology was performed through unweighted averaging of models in a heterogeneous ensemble of four CNNs, including *Darknet-19*, *MobileNetV2*, *ShuffleNet*, and *NASNet-Mobile*^30–33^. The purpose of choosing these specific networks was three-fold. First, the best available CNN architecture for RBC morphology classification was unknown, and therefore experimentation with multiple models was required. Second, we wanted to ascertain the benefits of ensembling through unweighted averaging. Finally, we wanted to maximize the accessibility of our code^28,29^ by picking popular networks that are readily available as part of the Deep Learning Toolbox™ in MATLAB, which is a ubiquitous programming and computing platform. Each CNN was validated individually and cumulatively (as part of the ensemble) against the MH test set of 1,335 images of individual RBCs.

### Singular CNN analysis: MH dataset

**Figure 3:**
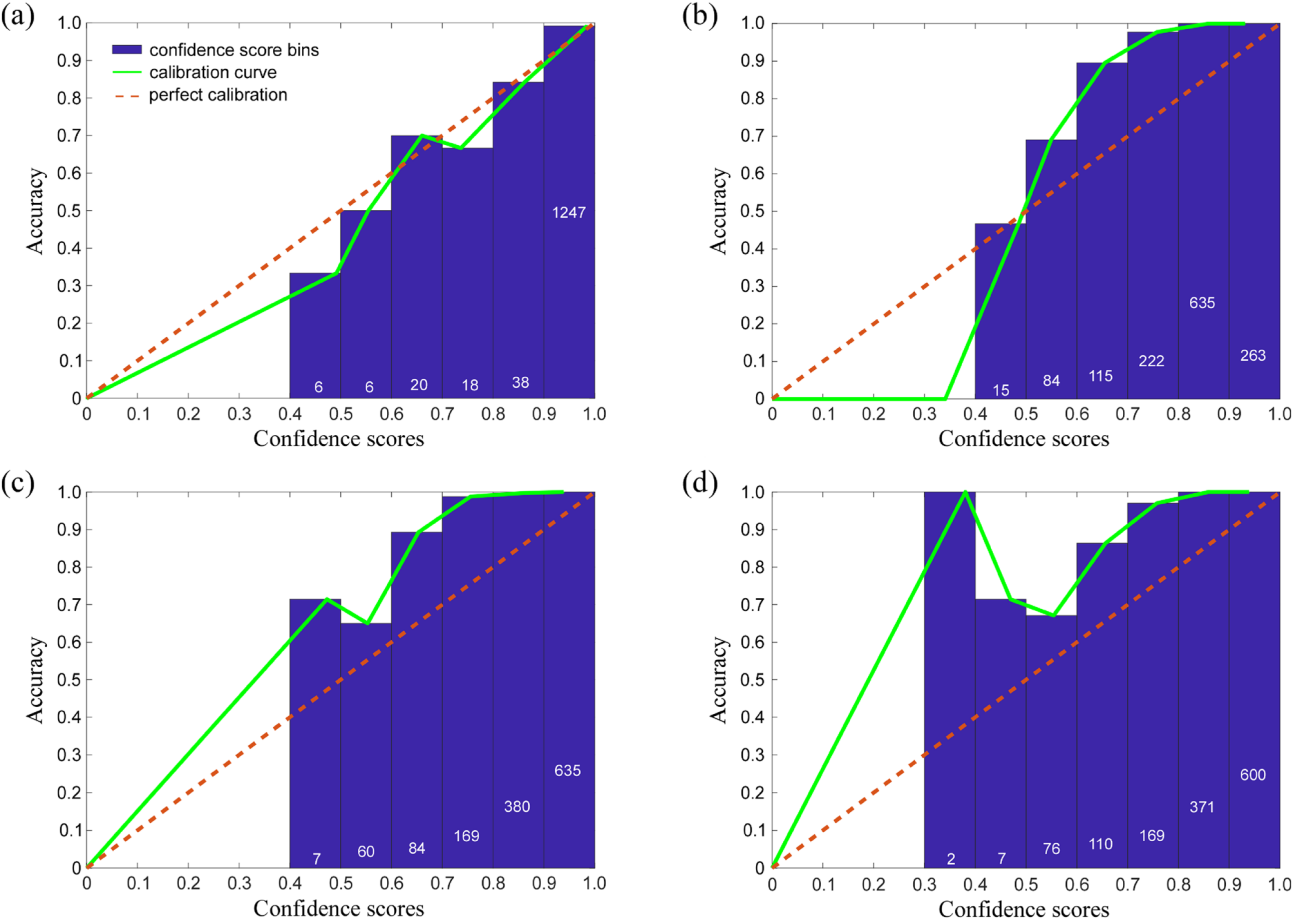
The reliability diagrams for the four convolutional neural network architectures (CNNs) trained to classify RBC images in the MH dataset, including (**a**) Darknet-19 (ECE: 0.96%; MCE: 15.84%), (**b**) MobileNetV2 (ECE: 14.8%; MCE: 34.08%), (**c**) ShuffleNet (ECE: 11.82%; MCE: 24.13%), and (**d**) NASNet-Mobile (ECE: 11.95%; MCE: 61.95%). Blue bars show the average accuracy for confidence scores within a certain range (bin); for each bin, the number of predictions with the confidence score falling within the range of that bin is indicated on the corresponding bar (white font). The calibration curve connects each bin’s average confidence and accuracy; perfect calibration is when the confidence score equals the prediction accuracy. ECE is the ‘expected calibration error,’ which is a weighted average of the difference between bin accuracy and confidence, where weight is the proportion of all confidence scores that fall within a particular bin. MCE is the ‘maximum calibration error,’ which indicates the largest difference between bin accuracy and confidence overall.

Figure 3 shows the reliability diagrams for each of the four CNNs used in this study, which we compared with their overall accuracy to assess their impact on the ensemble. *Darknet-19* had nearly ideal calibration (ECE: 0.96%) aside from some overconfidence (MCE: 15.84%) at the 0.4 to 0.5 confidence interval (Fig. 3a). The *MobileNetV2*’s reliability diagram indicated the greatest deviation from perfect calibration (ECE: 14.8%), and it showed under-confidence on the interval from 0.5 to 0.99; model accuracy was greater than its confidence scores for these values (Fig. 3b). *ShuffleNet* was under-confident on the interval from 0.4 to 0.99 (Fig. 3c). Finally, *NASNet-Mobile* was extremely under-confident (MCE: 61.95%) from 0.3 to 0.99 (Fig. 3d). When tested individually, *Darknet-19*, *MobileNetV2*, *ShuffleNet*, and *NASNet-Mobile* had overall predictive accuracies of 97.4%, 96.1%, 97.4%, and 96.5%, respectively (Fig. S13A).

An analysis of noise robustness also demonstrated Darknet’s superiority at handing gaussian & speckle noise compared to the other models and the ensemble (Fig. S1). Therefore, our validation results indicate that *Darknet-19* with random oversampling is the better model for RBC morphology classification. Although *Darknet-19* had the same overall accuracy as *ShuffleNet* with focal loss, *Darknet-19* was more robust against noise and had the lowest expected and maximum calibration errors (Fig. 3). This indicates that *Darknet-19* was the more objective classifier, as all other models were below 90% accuracy at less than 10% noise and were typically underconfident. However, the under-confidence of the weaker learners may be desirable within an ensemble. Underconfident learners give lower confidence scores, and since our ensemble used the highest confidence score in an unweighted average of confidence scores to predict RBC morphological class, an underconfident weak learner should contribute less to the prediction than a perfectly calibrated or overconfident model. In other words, if an underconfident prediction was wrong, it would have less impact on the average’s highest score, thus reducing the error.

### Deep ensemble analysis: MH dataset

**Figure 4:**
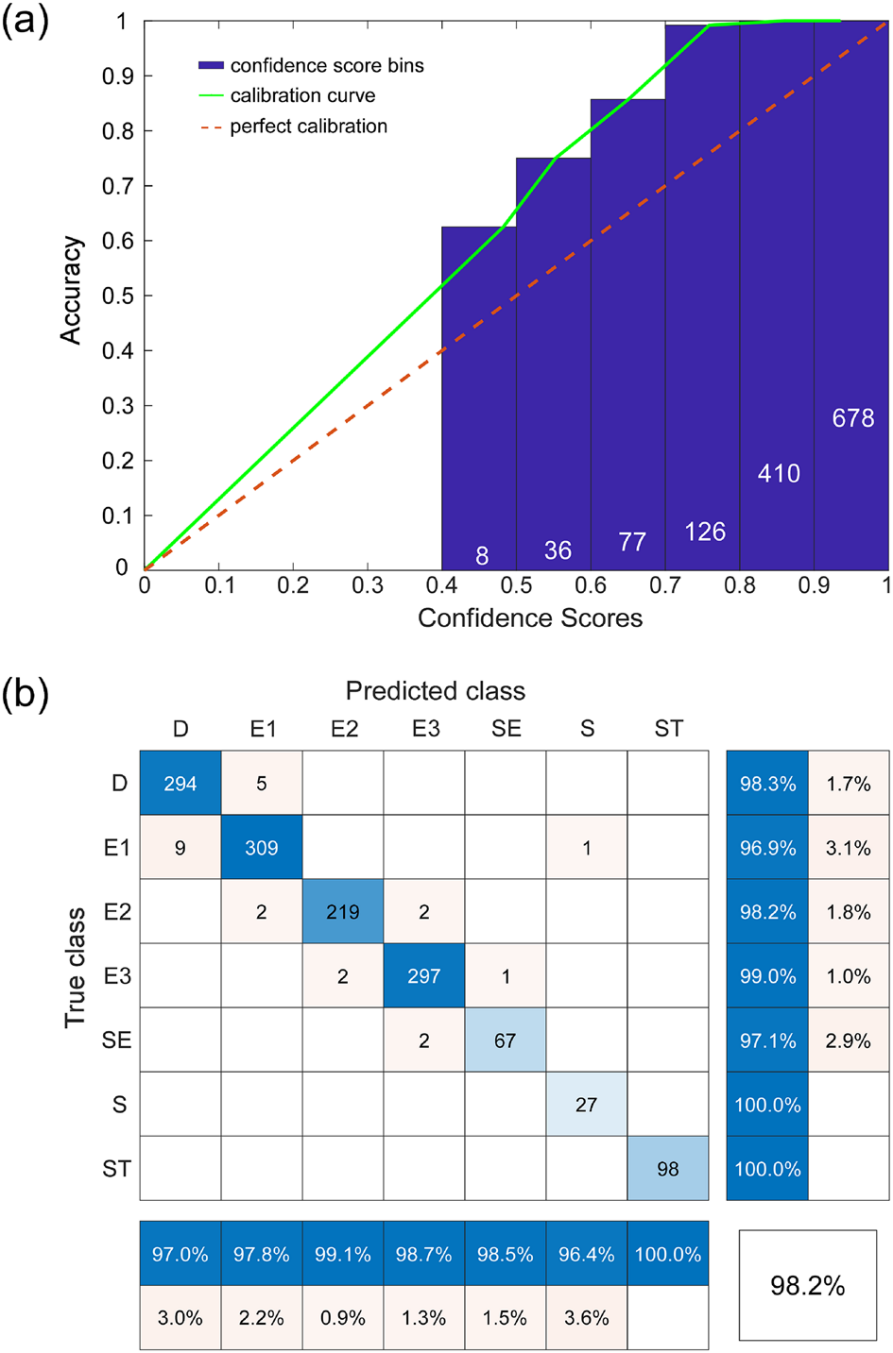
Accuracy of RBC morphology classification using the deep CNN ensemble applied to the MH validation set. (**a**) The ensemble reliability diagram (ECE: 11.6%; MCE: 23.31%). (**b**) The confusion matrix for the ensemble. Each RBC was classified as belonging to one of the following morphological classes: discocyte (D), echinocyte 1 (E1), echinocyte 2 (E2), echinocyte 3 (E3), sphero-echinocyte (SE), spherocyte (S), and stomatocyte (ST).

Figure 4 provides a deeper insight into the predictive capabilities of the ensemble of the four CNNs. Beginning with reliability testing, we checked the objectivity of the ensemble’s confidence scores (Fig. 4a). The deep ensemble was under-confident across the entire range, like three of the four constituent CNN models. As indicated by the confusion matrix, the deep CNN ensemble achieved high class precision rate/low false discovery rate (blue/orange bottom rows) and high recall/low false-negative rate (blue/orange far-right columns) across all morphology classes (Fig. 4b). The white cell in the bottom right corner, where precision and recall meet, is the overall accuracy of the ensemble, which was 98.2% (a value slightly above the best constituent CNN model). The main diagonal of cells in various tones of blue represents correctly classified images, whereas off-diagonal cells in shades of orange are incorrectly classified RBCs. Looking at the few incorrectly classified RBCs, one can see that most are off by a single class. For example, in the E3 predicted column, one sees cells misclassified as E2 or SE. It is likely that these “off-by-one” errors were due to the continuous nature of the storage-induced transformation of RBC morphology being expressed as discrete categories.

As complex models that make few assumptions about the underlying data, deep CNNs have high variance and low bias^24^. Ensembling deep CNNs through an unweighted average is one of many techniques to decrease variance and help the model generalize to unseen data. However, some literature speaks against ensembling heterogeneous networks through unweighted averaging because an overconfident weak learner may dominate the ensemble^24^. The ensemble validation results, as well as the random sampling test results, indicate that overfitting was not an issue in our study, and the ensemble could, in fact, generalize to unseen RBC images more accurately than any singular CNN (Fig. 4). (Supplementary Fig. S10A–G show a subset of 100 randomly selected images for each morphology class.) Still, the caveat of an increase in inference time for a marginal increase in accuracy remains, and one must therefore weigh this benefit against a project's time limitations when using the ensemble.

The classification accuracy demonstrated by the CNN ensemble was about 25 percentage points higher than what we had achieved using the binary decision-tree approach in our earlier study^5^. With an accuracy of > 98%, the CNN ensemble could potentially replace manual morphology classification in many research applications. Moreover, our code was developed using a widely available computational platform (MATLAB), and both the code and the MH dataset are freely available to the readers for testing and modification^28,29^. We expect that the ability to classify large numbers of RBC images automatically will increase the accessibility and reproducibility of morphological analysis performed in blood storage and transfusion research.

### Using the automated DL-enabled classification to measure cell diameter distribution for each morphological class: MH dataset

One of the goals of our original study that generated the MH dataset was to determine how the effective diameter of human RBCs change through the echinocytic transformation to aid the design of novel microfluidic devices for separating and removing SE and S cells from units of stored RBCs^5^. In the current study, we applied the DL-enabled RBC classification pipeline to the whole MH dataset to segment and classify the images de novo and to calculate the effective diameter of each cell. To increase the usefulness of our results, we implemented a rigorous data cleaning procedure that consisted of a low- and high-resolution phase (described in detail in the Supplementary Information, Fig. S4–S12).

Briefly, both phases made extensive use of normality testing and random RBC image sampling to gauge the influence of segmentation errors, as well as partially visible, aggregate, and poorly oriented cells. The primary difference between these phases was that the low-resolution phase cleaned the raw image data by removing statistical outliers, whereas the high-resolution phase relied on a trained *Darknet-19* classifier to separate good from bad standardized and upscaled (to 227 × 227 pixels) RBC images. The overall number of individual RBCs in the MH dataset was reduced from 1,826,730 to 1,294,996 after the data cleaning procedure. Additionally, we used a previously collected dataset of 37,273 images of fresh RBCs obtained from healthy volunteers^5^ to validate the effective diameter calculation. The mean effective diameter for fresh discocytes was 7.65 ± 0.45 μm (see supplementary Table S1), consistent with classical values and previously published literature^5^.

**Figure 5:**
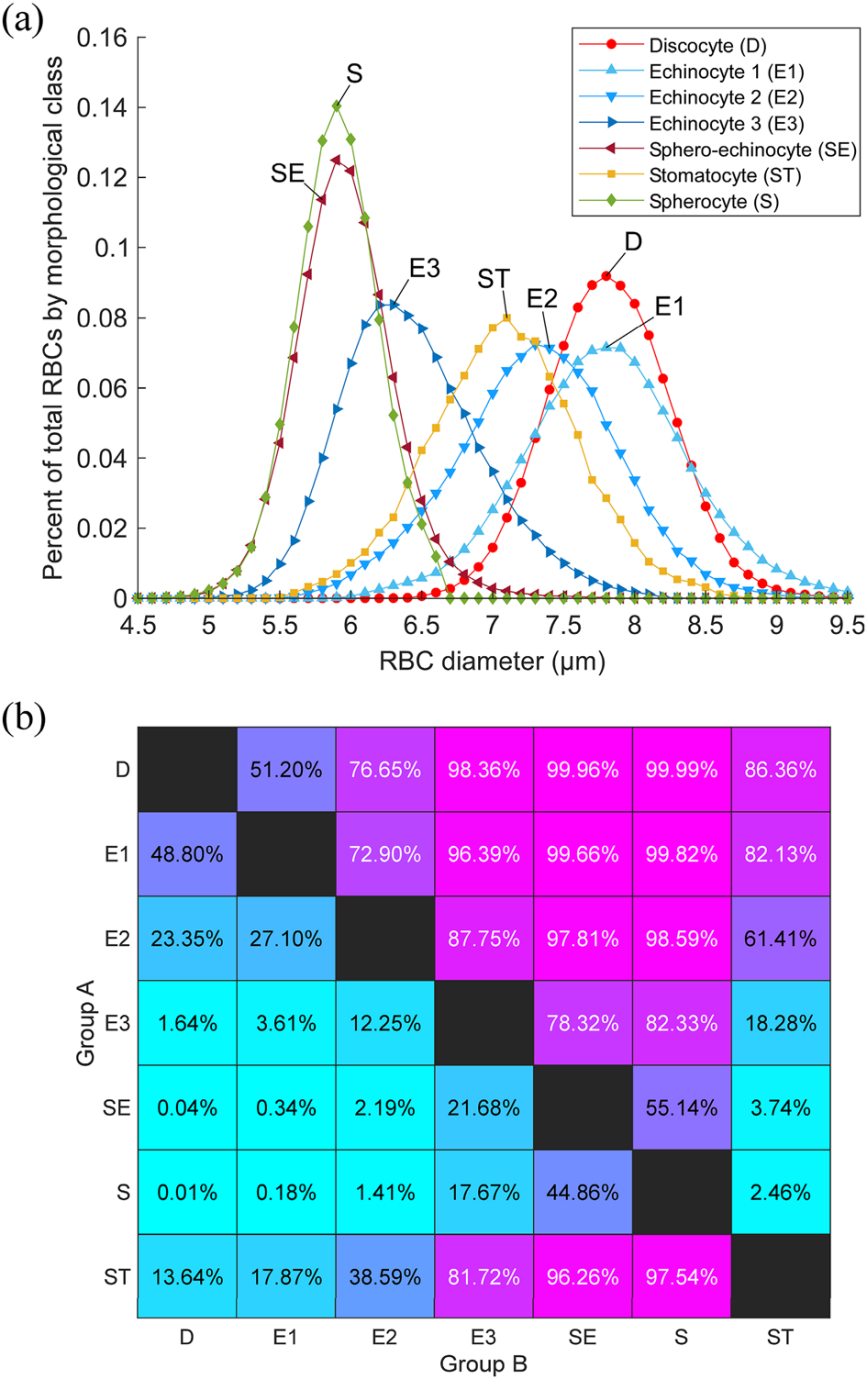
Dependence of effective diameter on RBC morphology. (**a**) Histograms of RBC effective diameters for each morphological class were compiled by classifying the cleaned MH dataset (n = 1,294,996) using the deep CNN ensemble. (**b**) The common-language effect size matrix for the effective RBC diameter of each morphological class shows the probability that a random sample from morphology group A (rows) will have a greater effective diameter than a random sample from morphology group B (columns).

Figure 5 shows the RBC diameter distributions for each morphological class. The diameter histograms were created by binning values from 4 to 10 μm at intervals of 0.1 μm and plotting the bin x-axis centers against the bin counts divided by their sum to indicate the frequency within each morphological class (Fig. 5a). Table 1 shows the descriptive statistics for each diameter distribution, in which we used 10,000 replicates of bias-corrected bootstrapping to calculate the 95% confidence intervals (CI) for the mean, standard deviation (SD), median and interquartile range (IQR) (for details see Fig. S12). Although hypothesis testing determined that the mean and median diameters of all classes were significantly different (p < 0.05), the magnitude of the effect sizes for most neighboring class comparisons (i.e., S vs. SE, E2 vs. ST, and D vs. E1) were small (Cohen’s d < 0.5, see Table S2).

**Table 1:** Descriptive statistics for the effective diameter distributions of each morphological class using high-resolution images (227 × 227 pixels) of individually segmented RBCs from the cleaned MH dataset classified by the deep CNN ensemble (n = 1,294,996).

Morphology Class	Mean ± SD [μm]	Mean 95% CI [μm]	SD 95% CI [μm]	Mean MAD [μm]	Median (IQR) [μm]	Median 95% CI [μm]	IQR 95% CI [μm]	Median MAD [μm]	Sample size
D	7.821 ± 0.429	7.819–7.824	0.427–0.431	0.342	7.816 (0.580)	7.813–7.819	0.577–0.584	0.290	121,571
E1	7.800 ± 0.581	7.799–7.802	0.580–0.583	0.458	7.798 (0.753)	7.795–7.800	0.750–0.756	0.377	368,292
E2	7.304 ± 0.567	7.301–7.307	0.565–0.570	0.451	7.318 (0.753)	7.314–7.322	0.748–0.758	0.376	112,885
E3	6.433 ± 0.490	6.431–6.435	0.488–0.491	0.390	6.387 (0.655)	6.384–6.390	0.651–0.659	0.324	167,345
SE	5.963 ± 0.348	5.961–5.964	0.347–0.349	0.268	5.948 (0.433)	5.947–5.950	0.431–0.435	0.216	273,332
S	5.904 ± 0.292	5.903–5.905	0.291–0.293	0.231	5.905 (0.385)	5.904–5.907	0.384–0.387	0.193	224,930
ST	7.080 ± 0.522	7.074–7.087	0.518–0.527	0.415	7.084 (0.696)	7.076–7.092	0.685–0.706	0.348	26,641

To further clarify the effect size of morphology effective mean diameter, we reframed effect size as a probability of diameter superiority using McGraw and Wong's common-language effect size^34^. The matrix in Fig. 5b shows the probability that a random sample from morphology class A (rows) will have a greater effective diameter than a random sample from morphology class B (columns). For example, there is only a 55.14% chance that a randomly sampled sphero-echinocyte (SE) will have a greater effective diameter than a randomly sampled spherocyte (S) (Fig. 5b). Likewise, if one were to put a randomly chosen discocyte (D) next to a randomly chosen stage 1 echinocyte (E1), there is only a 51.20% chance—or a slightly biased coin-toss—that the discocyte (D) will have a greater diameter (Fig. 5b). Therefore, even though each morphology class has an effective diameter distribution with a distinct mean, one cannot use diameter alone to differentiate between neighboring morphology classes because of the overlap between their distributions.

Improved classification accuracy (98% vs. 73%), and a rigorous data cleaning procedure enabled us to group RBCs into better-defined morphological classes, eliminating artifacts that were present in the original study^5^. Along with the random samples, the RBC diameter distributions for each morphology class (Fig. 5a) reflect this improvement via better approximation of the normal distribution. For example, the distributions for E1, E2, and E3 appear now as distinct classes with well-defined mean diameters (Fig. 5a). Similarly, a small (but significant) difference between SE and S is now apparent, which speaks to sphero-echinocytes still having some surface area to lose (Fig. 5a). Finally, the substantial difference and minimal overlap between the size distributions for S/SE and D classes support the possibility to sort these RBC types by their effective diameters, which may have significant implications for transfusion therapy^25,35^.

### Testing the robustness of the DL-enabled RBC classification pipeline: CIW dataset

**Figure 6:**
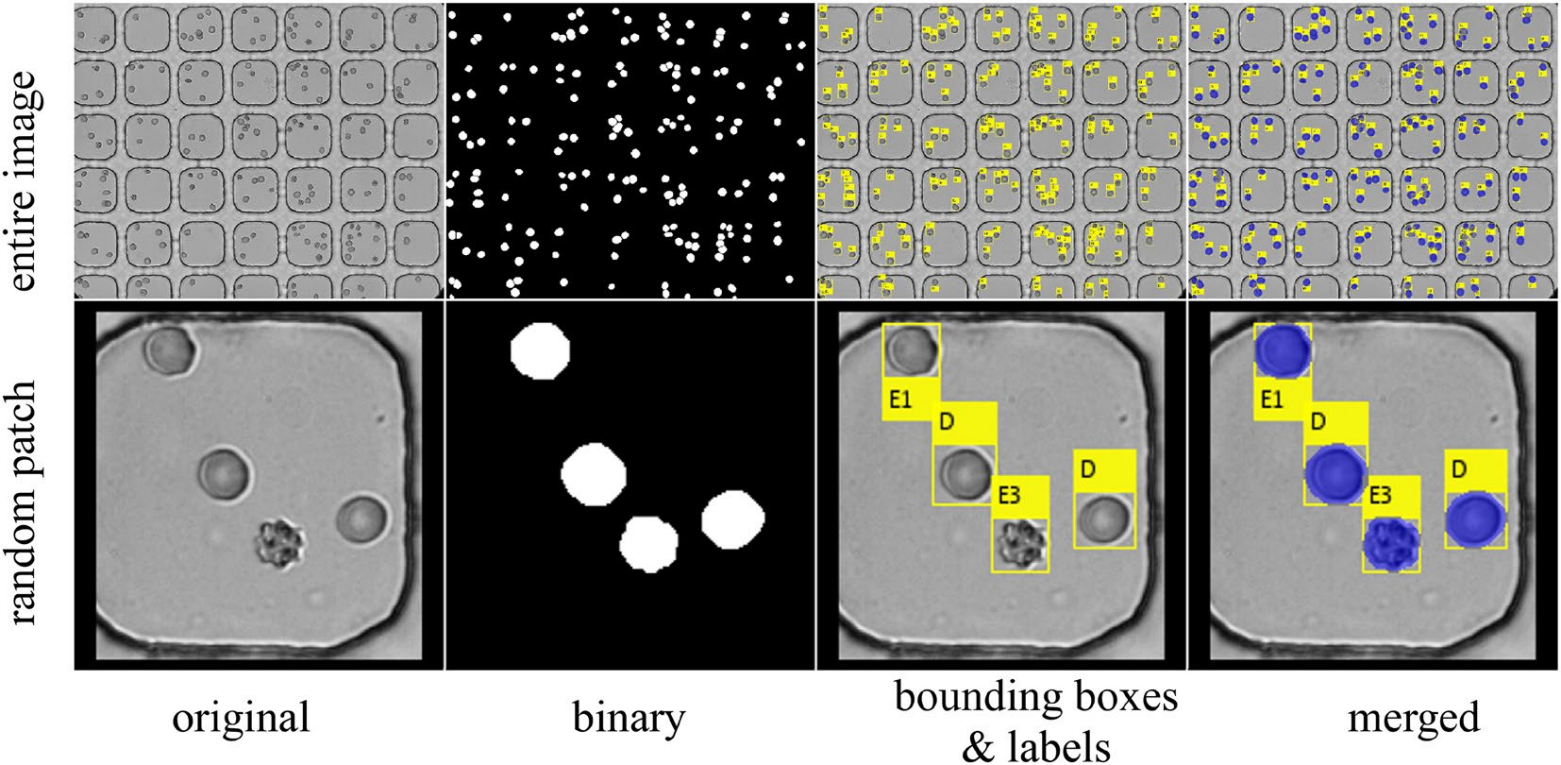
Application of the DL-enabled RBC morphology classification pipeline with a re-trained CNN ensemble to images from the CIW dataset. A random image from the CIW dataset showing the binary mask, bounding boxes, and RBC labels superimposed on the original image.

Next, we wanted to test the robustness of our newly developed classification pipeline by applying it to images from the CIW dataset^25,27^. Figure 6 illustrates the result of our analysis. Because the CIW images had the microfluidic wells in the foreground, we enhanced our preprocessing algorithm with semantic segmentation, which utilized two separate pre-trained *DeepLabv3* models, one for segmenting the wells and the other for segmenting RBCs within each well. The well segmentation function classified each pixel into ‘background’ or ‘well’, resized and cleaned the mask through the morphological opening, and performed blob analysis to find the well-bounding boxes. For each well, the RBC segmentation function removed specks through morphological opening, filled small holes via dilation and applied the watershed algorithm to delineate touching cells. A blob analysis of the resulting binary mask produced area, centroid, and bounding box information for each detected RBC. A subset of the segmented images from the CIW dataset was then manually curated and classified by an expert to create a pre-classified set of 5,000 images of individual RBCs. When applied ‘as-is’ to these pre-classified CIW images, the CNN ensemble (previously trained and validated on the MH dataset) showed a relatively low classification accuracy of 36.3%, with most errors caused by poor recognition of RBCs belonging to the SE and S morphology classes (Fig. S13B).

### Singular CNN analysis: CIW dataset

**Figure 7:**
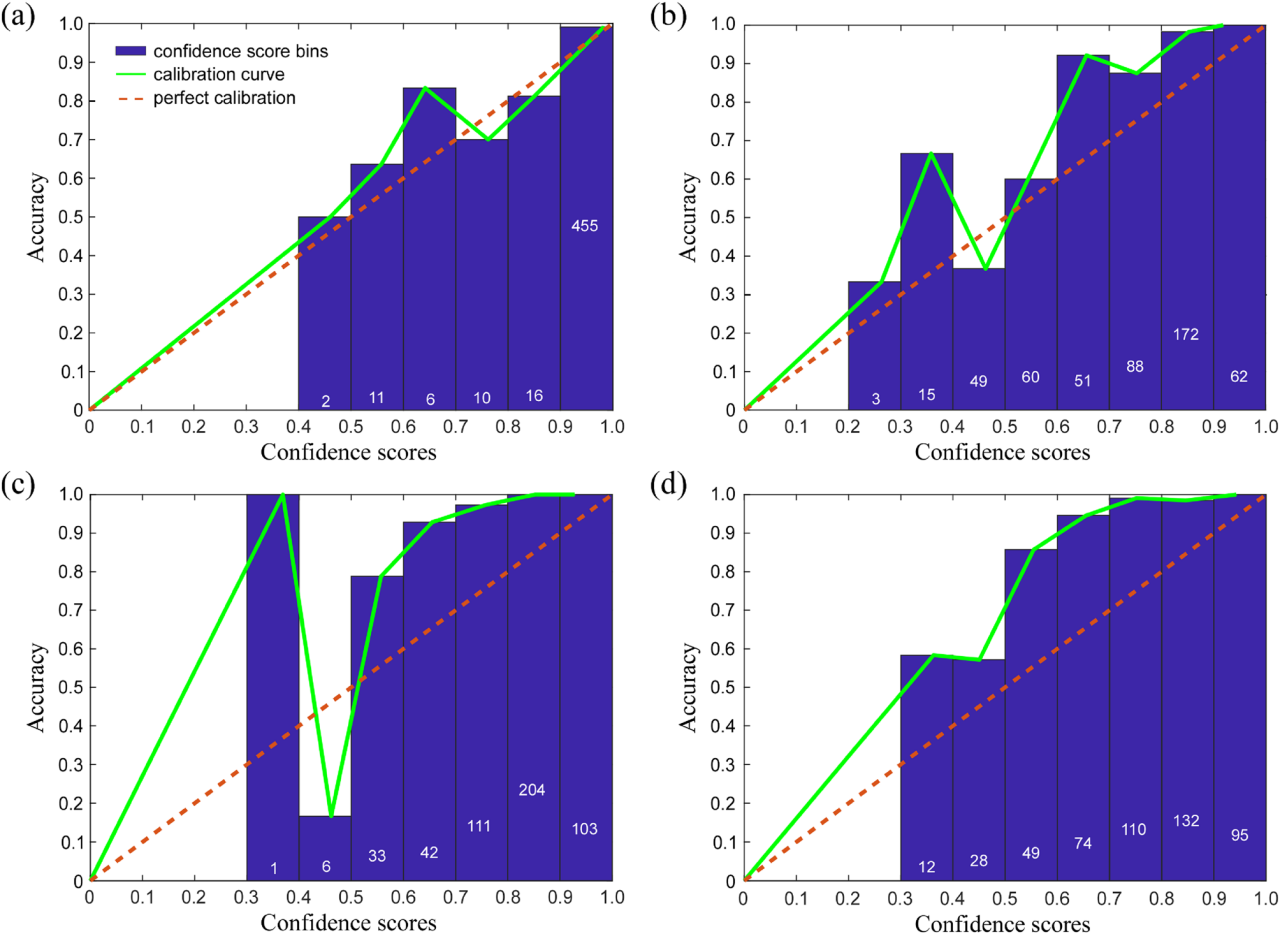
The reliability diagrams for the four original deep CNNs re-trained to classify RBC images from the CIW dataset, including (**a**) Darknet-19 (ECE: 1.44%, MCE: 19.18%), (**b**) MobileNetV2 (ECE: 12.91%, MCE: 30.85%), (**c**) ShuffleNet (ECE: 16.61%, MCE: 63.11%), and (**d**) NASNet-Mobile (ECE: 18.48%, MCE: 30.25%). (NOTE: For notations, please see the caption of Fig. 3).

To restore the classification accuracy of the CNN ensemble, we combined the MH training set with the CIW training set (90% of the pre-classified CIW set) and re-trained each CNN. We then used the CIW test set (10% holdout) to re-evaluate the performance of each re-trained CNN and of the re-trained CNN ensemble overall (Fig. S13C). Figure 7 shows the reliability diagrams of the re-trained networks. The re-trained networks had calibration characteristics similar to what we observed for the MH dataset (Fig. 3). *Darknet-19* had a nearly perfect calibration and the lowest values of ECE and MCE (Fig. 7a). *MobileNetV2* was generally under-confident (Fig. 7b). *ShuffleNet* was also mostly under-confident, albeit displaying some over-confidence and a large MCE for confidence scores ranging from 0.4 to 0.5 (Fig. 7c). Finally, *NASNet-Mobile* was the most under-confident model (ECE: 18.48%) in the ensemble (Fig. 7d). When tested individually against the CIW test set, *Darknet-19*, *MobileNetV2*, *ShuffleNet*, and *NASNet-Mobile* showed classification accuracies of 96.8%, 84%, 96.4%, and 93.8%, respectively.

### Deep ensemble analysis: CIW dataset

**Figure 8:**
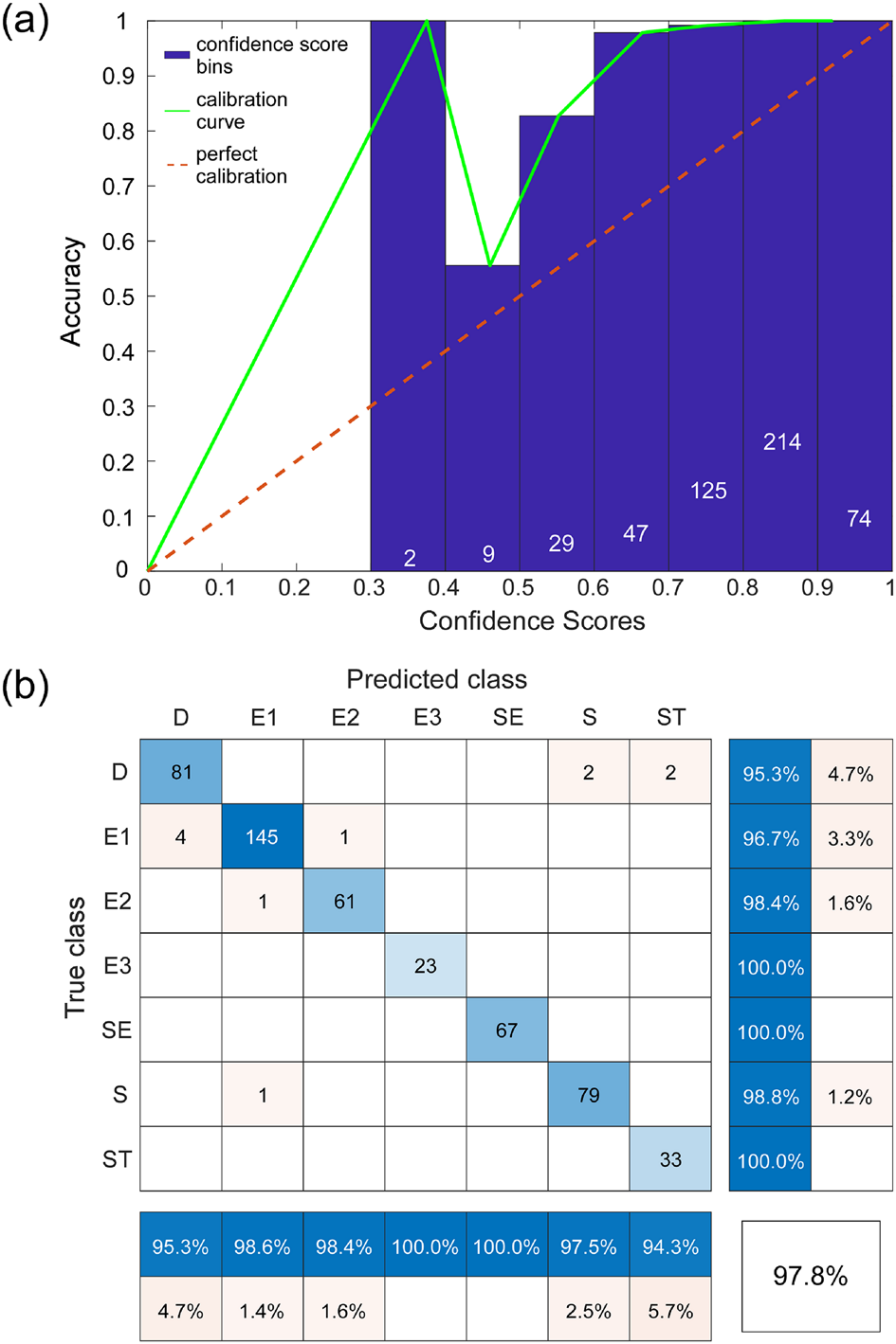
Accuracy of RBC morphology classification using the re-trained deep CNN ensemble applied to the CIW validation set. (**a**) The ensemble reliability diagram (ECE: 18.33%; MCE: 62.49%). (**b**) The confusion matrix for the ensemble.

Figure 8 shows the reliability diagram (Fig. 8a) and the confusion matrix (Fig. 8b) for the classification of RBC morphology using an ensemble of re-trained CNNs applied to the CIW test set. The overall classification accuracy of the re-trained CNN ensemble on the CIW dataset was 97.8%, which was an improvement of 61.5 percentage points over the accuracy of the original CNN ensemble trained only on the MH dataset and applied directly to the CIW dataset.

These results suggest that our DL-enabled classification pipeline developed and validated initially for the MH dataset was not sufficiently robust to deal with relatively common changes in the source images (i.e., the addition of microfluidic wells and slightly different image acquisition parameters) that were characteristic of the CIW dataset (Fig. S14). However, generalizing the segmentation algorithm to recognize foreground features and re-training the CNNs on a training set expanded with a manually curated subset of images from the CIW dataset proved a highly effective solution. The resulting classification accuracy of the re-trained CNN ensemble on the CIW test set (97.8%, Fig. 8b) was only slightly lower than the accuracy of the original CNN ensemble on the MH test set (98.2%, see Fig. 4b), sacrificing a little accuracy for a substantial improvement in robustness. To simplify the adoption, support the reproducibility of our results, and further progress in the field, we released the MH and CIW datasets into the public domain^26,27^ and provided the code itself under an unrestrictive MIT open source license^28,29^.

### Discovering new shape recovery modalities using automated morphology classification: CIW dataset

The images of the CIW dataset depict RBCs as they undergo shape recovery following the replacement of their storage medium with a fresh washing solution, either normal saline or 1% solution of human serum albumin (HSA)^25^. Thus, each RBC from the CIW dataset has a unique time dimension (history) that cells from MH dataset lack. In our original study, a human expert manually classified and tracked select RBCs for the duration of the experiment to document the evolution of their shape change due to washing^25^. In the current study, we tested if our DL-enable morphology classification pipeline can provide a deeper insight into washing-induced shape recovery of stored RBCs. After segmentation and classification of RBCs in each frame, each cell’s parameters were passed to a persistent data structure that tracked each identified RBC through a Kalman filter predicting the cell’s future position in the subsequent frame and making the appropriate associations from a variant of Hungarian assignment. As in the original study, we used the classification data to identify RBCs that underwent a change in shape by the 10-min mark (for example, see Fig. S15).

**Figure 9:**
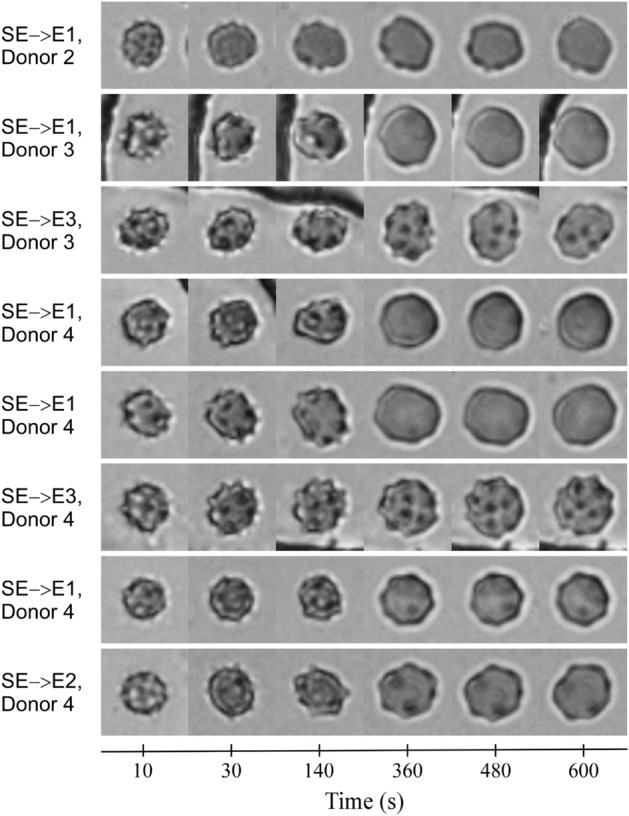
Representative examples of RBCs classified as sphero-echinocytes (SE) recovering shape to various stages of echinocytes (E1, E2, and E3) after washing with HSA identified by the deep CNN ensemble in the CIW dataset.

Figure 9 shows representative examples of RBCs classified as SE recovering their shape to join the E3, E2, and even E1 morphological classes. We found visual evidence of these transformations in several (but not all) donors, and only for washing with 1% HSA. Discovering these anomalous transformations was particularly surprising because one of the findings from our previous research was that washing did not improve the shape of sphero-echinocytes which we assumed was because the loss of surface area by SE cells was too great^25^. Indeed, the mean diameter of E1 is over three standard deviations greater than the mean diameter of SE cells (Table 1), making SE to E1 shape recovery very unlikely, but evidently not impossible (Fig. 9).

High classification accuracy and automated cell tracking enabled us to discover a new mode of shape recovery that was missed by a human expert in the previous study. The notorious tediousness of manual morphology classification, the relative rarity of SE to E shape recovery, and potential unconscious bias against this transformation due to pre-existing knowledge may have contributed to the omission. Our results demonstrate that these factors can be effectively mitigated by an accurate, automated cell tracker and classifier. Furthermore, our findings suggest new opportunities for screening novel rejuvenation regiments, storage solutions, and drug candidates through automated morphology classification and tracking massive numbers of stored RBCs automatically.

## Limitations of the study

Most limitations of our research involve its scope, which is not exhaustive. We do not compare our pipeline to commercially available systems, test all available CNNs or ensembling methods, or try different microscopes, objectives, and point spread functions (PSFs). The preliminary nature of our research means that our study of the image analysis pipeline is specific to the particular microscope/camera setup used to acquire the images, the specific CNN architectures we chose, and ensembling using unweighted averaging. The pipeline’s code is available for other researchers to alter and compare with other systems. Additionally, our system is data-driven, so images meant for scientific analysis must resemble the pictures on which the ensemble of deep neural networks is trained. This is illustrated when we attempt to apply the system trained on the MH dataset to the CIW dataset, which are images taken with the same microscope but with a different foreground. Accuracy suffered until we retrained the ensemble using a small subset of CIW images, and we anticipate a similar situation when using images from other microscopes.

## Materials and methods

### Datasets

This study used two separate sets of RBC images acquired with an inverted bright-field microscope (IX79, Olympus American, Inc., Center Valley, PA) and a high-speed camera (MC1362, Mikrotron GmbH, Unterschleisheim, Germany) for two separate studies previously published by our research group^5,25^. Both datasets have been made publicly available through the UH Dataverse Repository (https://dataverse.tdl.org/dataverse/erythrocyte).^26,27^.

The first dataset, herein referred to as ‘**Morphological Heterogeneity**’ (**MH**), was collected for a study that described a new automated system for classifying the morphology of stored RBCs^5^. The system comprised a microfluidic device for acquiring high-quality images and a decision-tree algorithm for segmenting and classifying the images. To generate the MH dataset, seven units of stored RBCs were purchased from two regional blood centers. The units were kept at 2–6 °C in a blood bank refrigerator and sampled at 6, 7, and 8 weeks of storage. The samples were diluted to approximately 1% hematocrit and passed through the microfluidic device to acquire images of stored RBCs flowing through the field of view of the microscope (at 64 × magnification). The images (1280 × 1024, grayscale) were acquired every 5 s for 30 min (100 fps, 9.617 ms exposure, global shutter) using blue light illumination to enhance contrast (red cells appear dark in blue light). Image acquisition experiments were repeated several times for each unit and storage duration to increase the number of RBC images available for analysis^5^. The MH dataset used in this study included all of the acquired images (including those that were omitted in the original study) which, when properly cleaned and segmented, yielded n = 1,294,996 of individual RBCs. A subset of the MH dataset was pre-classified by an expert to create a set of n = 13,353 images of individual RBCs classified into 7 morphology classes. This pre-classified set was split into an MH training set (90%) to train the CNNs used in this study and an MH test set (10% holdout) to test the classification accuracy of the trained CNN ensemble.

The second dataset, herein referred to as ‘**Cells-In-Wells**’ (**CIW**), was collected for a study which investigated the dynamics of shape recovery by stored RBCs after washing with normal saline or a 1% solution of human serum albumin (HSA)^25^. To generate the CIW dataset, RBC units collected from six different donors were purchased from a local blood center and stored in a blood bank refrigerator at 2–6 °C for up to 6 weeks. The units were sampled at 4 weeks (Donors 1 and 2), 5 weeks (Donors 3 and 5) and 6 weeks (Donors 4 and 6) of hypothermic storage, and the samples were diluted with autologous storage medium to 0.05% hematocrit. After mixing through gentle inversion for 5 min, aliquots of the diluted samples were deposited onto the microfluidic well arrays and washed by adding a large volume of normal saline, 1% HSA or the autologous storage medium (negative control). The microscopic images (at 40 × magnification) of RBCs confined in microfluidic wells were acquired at a rate of 1 frame per second for about 17 min (1,024 frames). Each image acquisition was of the same wells to track the change in shape of the same RBCs throughout the washing process^25^. The CIW dataset used in this study included all available image sequences which, when properly segmented and tracked, yielded 3,250 individual RBCs (and their associated longitudinal data following the shape change). A subset of the CIW dataset was pre-classified by an expert to create a set of 5,000 images of individual RBCs classified into 7 morphology classes. This pre-classified set was split into a CIW training set (90%) to re-train the CNNs initially trained on the MH images, and a CIW test set (10% holdout) to test the classification accuracy of the re-trained CNN ensemble.

### Hardware and software platforms

All computations were performed on a consumer-grade laptop (CPU: Intel i7-8750H, RAM: 16 GB DDR4, GPU: 8 GB NVIDIA GeForce RTX 2070 w/ Max-Q Design) running Windows 10 (Microsoft Corporation, Redmond, WA). All scripts, functions, and neural networks were written and trained in MATLAB R2021b (The MathWorks, Inc., Natick, MA). A conglomeration of MATLAB examples, tutorials, and research articles inspired the segmentation, deep learning, and cell tracking architecture of the image analysis pipeline developed in this study^36–42^. All code has been made publicly available through GitHub (https://github.com/BloodML)^28,29^ and the UH Dataverse Repository (https://dataverse.tdl.org/dataverse/erythrocyte).^26,27^.

### Image segmentation

Our segmentation method first attempts adaptive thresholding and then switches to a semantic segmentation approach if necessary. This stage of the image analysis pipeline requires MATLAB’s Computer Vision Toolbox™, Deep Learning Toolbox™, and Image Processing Toolbox™. The two processed datasets illustrate the difference between thresholding and semantic segmentation, as the backgrounds of the MH images are clear enough for thresholding, and the CIW images require semantic segmentation due to the presence of microfluidic wells in the foreground. Nevertheless, the former approach was used to bootstrap the latter by giving an expert a subset of CIW image masks that only required minor alterations to train deep learning pixel classifiers. Additionally, both segmentation methods used morphological dilations and erosions to remove specks and fill holes and the watershed algorithm to ensure that touching cells have dividing lines that differentiated their silhouettes.

Adaptive thresholding was nested in a broader segmentation function, which produced a binary mask for a target grayscale image given an adaptive thresholding statistic (mean, median, gaussian), the maximum radius in pixels of unwanted specks, and the H-minima transform scalar for the binary blob’s regional minima used in the watershed algorithm. Adaptive thresholding is robust with respect to variations in image illumination because a different threshold value is computed for each pixel given some neighborhood of surrounding pixels and a measure of their central tendency^43^. For the MH dataset, we used the median as the adaptive thresholding statistic (a pixel neighborhood size equal to one plus two times the floor of the image size divided by 16) and the foreground polarity set to dark. After adaptive thresholding returned the threshold matrix, the code binarized the image by setting all pixels above their threshold value to true and those equal or below to false. The code then filled holes in the center and borders of the image. Next, it morphologically eroded and dilated the new mask with a disk structuring element of radius 6 pixels. Finally, the watershed algorithm divided connecting cells by finding and masking the catchment basins around the negative distance transform’s regional minima from the inverse binary image. Because we needed regional minima small, we set the H-minima transform scalar to one.

Even when adaptive thresholding fails, one can manually gather and correct the segmentation efforts with an image labeler to train pixel classification networks. This is how the two *DeepLabv3* models—one for segmenting wells and the other for segmenting RBCs within the wells—were developed to segment the CIW dataset. We gathered full-sized images for the initial well segmentation and corrected the corresponding binary masks of all wells in the first 100 frames of each longitudinal recording. For the cells in the wells, we cropped and padded 10,327 individual wells to get 170 × 170-sized grayscale training images and binary cell masks. There was no validation set for either because our primary concern was how the models performed on the entire CIW dataset.

Before training the models, both two-class networks were made with *resnet-18* CNN backbones and modified with custom pixel classification layers that utilize focal Tversky loss^44–46^. Contrary to the available literature, we set the loss alpha hyperparameter to 0.7, beta to 0.3, and the focal loss gamma parameter to 0.75. These are likely suboptimal values, so both networks may benefit from tuning the loss function’s hyperparameters. Still, we achieved good segmentation results on the entire CIW dataset.

With respect to training the networks, there were only minor differences between the two sessions. The well segmentation model had its target image size reduced to half the scale of the full-sized images (640 × 512), and we set the minibatch size to 8 images. The RBC segmentation model increased its target image size to 224 × 224 and had a minibatch size of 32. Both used data augmentation to randomly scale images from 80 to 150% of their target size. Also, we randomly rotate images (0°, 90°, 180°, 270°) and convert the grayscale images to RGB. To further reduce the likelihood of overfitting, we set L2 regularization to 0.005. The training was done on the GPU using stochastic gradient descent with momentum set to 90% to minimize the loss function, and we shuffled the images every epoch. Each session lasted until loss stagnated and training accuracy plateaued.

Like adaptive thresholding, broader segmentation functions utilized the trained networks. The well segmentation function exported well-bounding boxes given the frame and the *DeepLabv3* well segmentation network. Internally it reduced the image size by half, classified each pixel into background or a well, and then resized and cleaned the mask through a morphological opening operation before MATLAB’s blob analyzer found the bounding boxes. The RBC segmentation function differed in that it cropped and padded the wells before resizing them to 224 × 224 and applying semantic segmentation. The resulting pixel label matrix was then converted to a binary RBC mask and was resized to 170 × 170 before removing the array padding around it. We removed specks through the morphological opening operation and filled small holes via dilation. In both instances, we employed a disk structuring element with a radius of 3 pixels for the opening operation and 2 pixels for dilation. Next, we used the watershed algorithm (described above for the adaptive thresholding approach) before removing blobs with areas under 100 [pixel^2^]. Finally, MATLAB’s blob analyzer yielded each RBC’s area, centroid, and bounding box as output.

### Classification via deep learning

Classification was performed through the unweighted averaging of models in a heterogeneous deep ensemble of four pre-trained CNNs, including *Darknet-19*, *MobileNetV2*, *ShuffleNet*, and *NASNet-Mobile* (available in MATLAB as part of the Deep Learning Toolbox™)^30–33^. For each CNN, the input layer was modified to directly receive 227 × 227 grayscale images. Although there was no alteration to either CNN’s preferred normalization technique, some required a new initial 2D convolution layer to ensure that the dimensions of the subsequent layer matched. Next, a new fully connected layer built for seven morphology classes was created with a weight and bias learning rate factor of 10. Then, each model had its old fully connected layer replaced with this newly created layer and a new classification layer. All models, except *DarkNet-19*, received a focal loss classification layer with hyperparameters alpha and gamma set to 0.25 and 2.0, respectively^47^. This modification was introduced to mitigate any potential bias that class imbalance might cause by dynamically scaling the cross-entropy loss function to make the network more sensitive to misclassified observations. *DarkNet-19* received a standard cross-entropy loss classification layer, as it dealt with class imbalance through random minority oversampling.

Random minority oversampling is when classes with fewer images than the majority class have their images randomly duplicated until all categories have the same number of images as the majority class. Since there is an equal number of pictures in each class, there is no longer an imbalance. However, the random minority oversampling approach fills the minority classes with random duplicates that may be easy to memorize, which may increase the likelihood of overfitting^48,49^.

Ensembling, data augmentation, and L2 regularization are ways to reduce model overfitting by encouraging the CNN to make better generalizations. These methods decrease the model's complexity, or variance, at the expense of a, hopefully small, increase in bias, Deep artificial neural networks have high variance and low bias, and ensembling several deep CNNs through an unweighted average can decrease variance and help the resulting ensemble generalize to unseen data^50^. In this study, the softmax scores of the CNNs in the ensemble were averaged, either by a single frame or through a moving average of 100 frames. Likewise, data augmentation decreases a CNN model’s variance by increasing the size of the training set. That is, a CNN is more likely to accurately classify unseen test samples given that larger training sample sizes more precisely capture the population average of each class^51^. In this study, the size of the training sets was increased by augmenting images through X & Y translations (± 100 pixels), rotations (0-360º), and scaling (75–130% of the image resolution). For the MH dataset, we also added masked duplicates of each RBC to enable the classification of individually segmented RBCs. L2 regularization (weight decay), is a parameter shrinkage method that represses a CNN model’s weights by adding a penalty term to the loss function^52^. It therefore decreases variance by increasing bias towards smaller weights. In this study, the L2 regularization coefficient for all CNN models was set to 0.005 to strike a balance between model simplicity and training data fitting.

Training sessions were carried out on the GPU (using the Parallel Computing Toolbox™) with a variable minibatch size between 10 and 32 randomly shuffled images for each model but with additional hyperparameters (such as learning rate and momentum) held constant. Based on the test set (10% holdout) results, early stopping was used with model checkpoints to decide when training should end (typically around 20 to 300 epochs). The learnable parameters were updated by minimizing loss through stochastic gradient descent with momentum. The initial learning rate was set to a low 3 × 10^–4^ to prevent training from reaching suboptimal results or diverging. Still, momentum was set at the high default value of 0.9 to allow the previous iterations to strongly influence the current update, potentially accelerating model convergence^53^.

### Cell tracking

In addition to image segmentation and classification, the analysis of CIW dataset required tracking the positions of each individual RBCs in each well longitudinally, to document the change in RBC morphology due to washing. For the initial frame, a new (blank) data structure was created to hold tracking information for each RBC, including a unique identifier (id), position and morphology scoring histories, age and visibility counts, and a Kalman filter object. For each unassigned RBC centroid and its associated data obtained from image segmentation and classification functions, a new cell track was created with a Kalman filter object initialized at the respective centroid. Although the motion of the RBCs appears random, through trial and error, we found that a constant velocity model was effective for the Kalman filter when initial location and velocity variance were set to 200 and 50, respectively, motion noise was the vector [100, 25], and the variance inaccuracy of detected location was set to 100.

For the next frame, each cell’s Kalman filter object was used to predict the location of the cell’s centroid in the current frame and thus make an association between existing cell tracks and unassigned RBC centroids detected by segmentation and classification of the current frame. The tracking function computed the distance between the predictions and each new detection to create a cost matrix. The James Munkres's variant of the Hungarian assignment algorithm was used to then assign cell tracks based on the cost matrix and an experimentally found value of 20 for the cost of not assigning a detection to a cell track^42,54,55^. Assigned detections were used to update the respective cell tracks. Existing cell tracks that did not have a detection associated with them had their age and consecutive invisibility counts incremented. To account for segmentation errors, tracks of cells that were (i) invisible for over 50 successive frames or (ii) less than eight frames old and visible less than 30% of the time were deleted. This tracking process was repeated for each subsequent frame.

### Statistical analysis

Statistical analysis was perform using Microsoft Excel or the built-in functions of the Statistics and Machine Learning Toolbox™. Sample normality was examined via descriptive statistics, normal probability plots, q-q plots, and normal distribution fitted histograms. Hypothesis testing was through the Kruskal–Wallis test, two-sample t-testing with Bonferroni adjustment, and 10,000 replicates of bias-corrected bootstrapping. Likewise, all 95% confidence intervals were calculated using 10,000 replicates of bias-corrected bootstrapping, Effect size was analyzed in terms of Cohen's d^56^ and the common-language effect size of McGraw and Wong^34^.

## Funding and Resources

This research was supported in part by the National Heart, Lung, and Blood Institute of the National Institutes of Health under awards R01HL117329 and R01HL151858 (PI: SSS). NY was supported by the Summer Undergraduate Research Scholarship (SURF) and the Provost's Undergraduate Research Scholarship (PURS) from the Office of Undergraduate Research, University of Houston. The content is solely the responsibility of the authors and does not necessarily represent the official views of the National Institutes of Health or the United States Government.

## Supplementary Information

Supplementary Information.

## Data Availability

All datasets generated and analyzed in the current study are available through the University of Houston Dataverse Repository (https://dataverse.tdl.org/dataverse/erythrocyte).^26,27^ All code developed in this study is available through GitHub (https://github.com/BloodML).^28,29^.
