# Supplementary material for: Deep ensemble learning enables highly accurate classification of stored red blood cell morphology
Source: Sci Rep. 2023 Feb 23;13:3152. doi: 10.1038/s41598-023-30214-w (PMC9950070; doi:10.1038/s41598-023-30214-w)
Supplement: Supplementary file 1 — Supplementary Information. [file 41598_2023_30214_MOESM1_ESM.docx]

**Supplementary Information**

**Deep ensemble learning enables highly accurate classification of stored red blood cell morphology**

Austin H. Routt, Natalia Yang, Nathaniel Z. Piety, Madeleine Lu, and Sergey S. Shevkoplyas*

Department of Biomedical Engineering, Cullen College of Engineering, University of Houston, Houston, TX 77204

# **Morphological Heterogeneity Dataset: Noise Robustness Analysis**

**Figure S1** shows images and graphs of the original and retrained models being tested on gaussian and speckle noise using the MH dataset. The percentage noise that S1A and D depict is the percentage of the variance of the noise over the signal for the entire image. First, the image is normalized between 0 and 1 through min-max normalization. The noise variance is then calculated by multiplying the noise parameter percentage with the normalized image's variance. The noise variance is then applied using the noise function, and the noisy image is multiplied by 255.

S1B and E show model accuracy as Gaussian (B) or Speckle (E) noise increases for the models trained on the MH training set. Given a threshold of 90% accuracy as the bare minimum acceptable for RBC classification, Darknet-19 is the more robust model for both types of noise. All other models, including the ensemble, were below 90% accuracy before 10% noise was added, but Darknet-19 was robust enough to handle images with about 30% Gaussian noise and 40% Speckle noise.

After testing the original models, we tested the retrained networks to see if the models became more or less robust against noise after training with additional images. Figures S1B and F indicate that models became less robust to Gaussian and Speckle noise after retraining. Note that for all models, the 90% threshold is reached at lower noise levels. Still, *Darknet-19* appears to remain the most robust model in the group.

**Figures S1**

| A)  **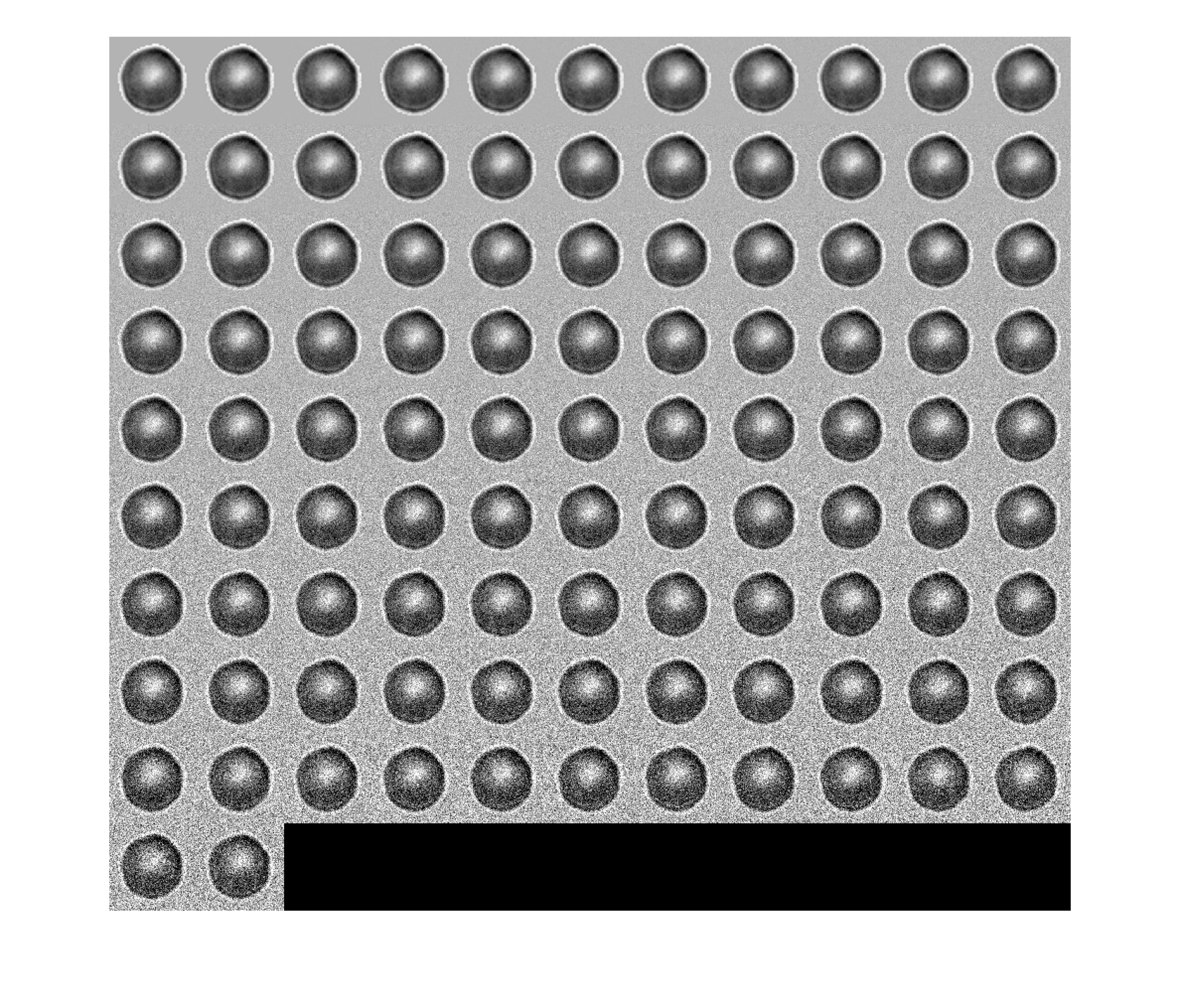** | D)  **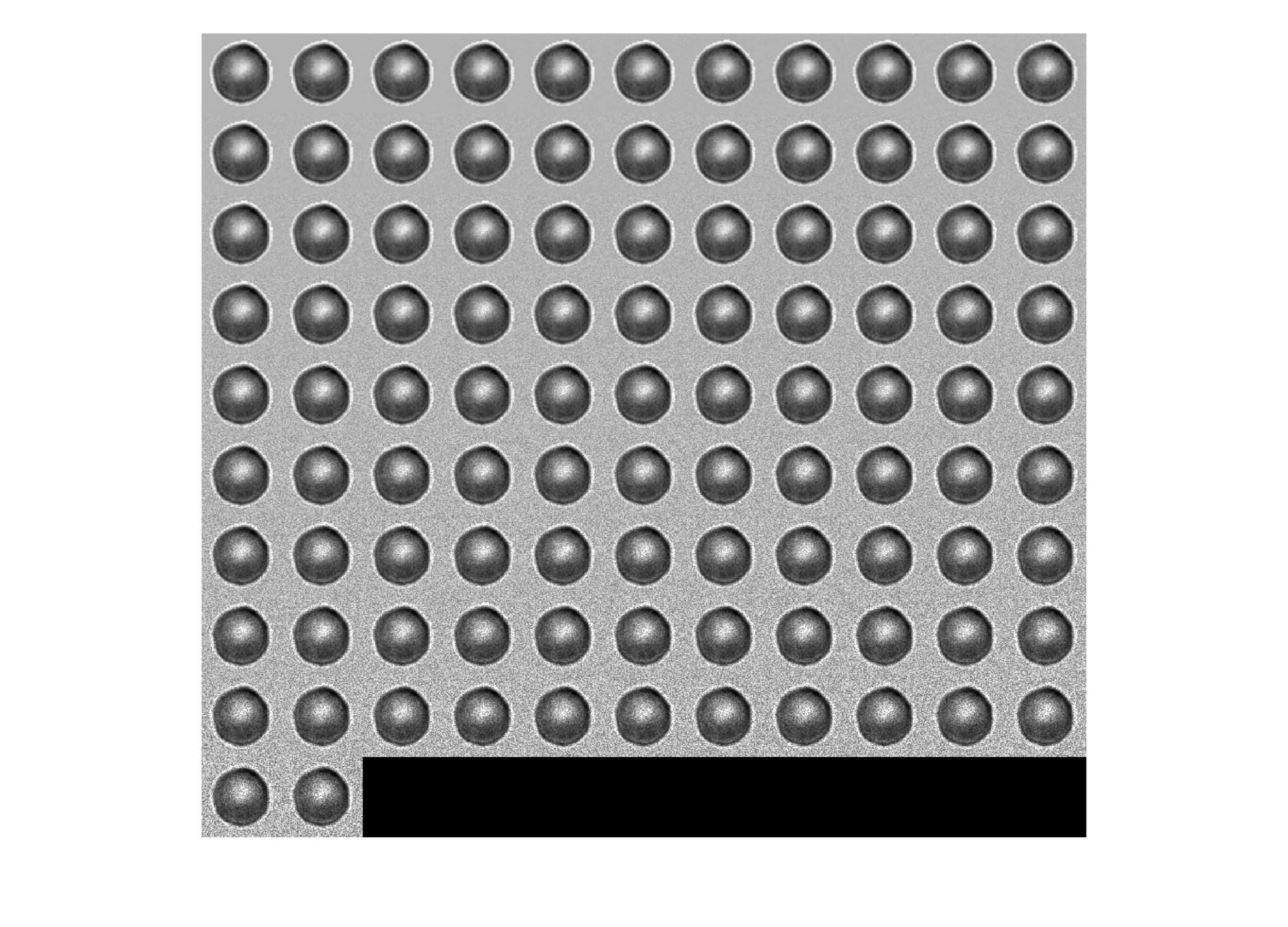** |
| --- | --- |
| B)  **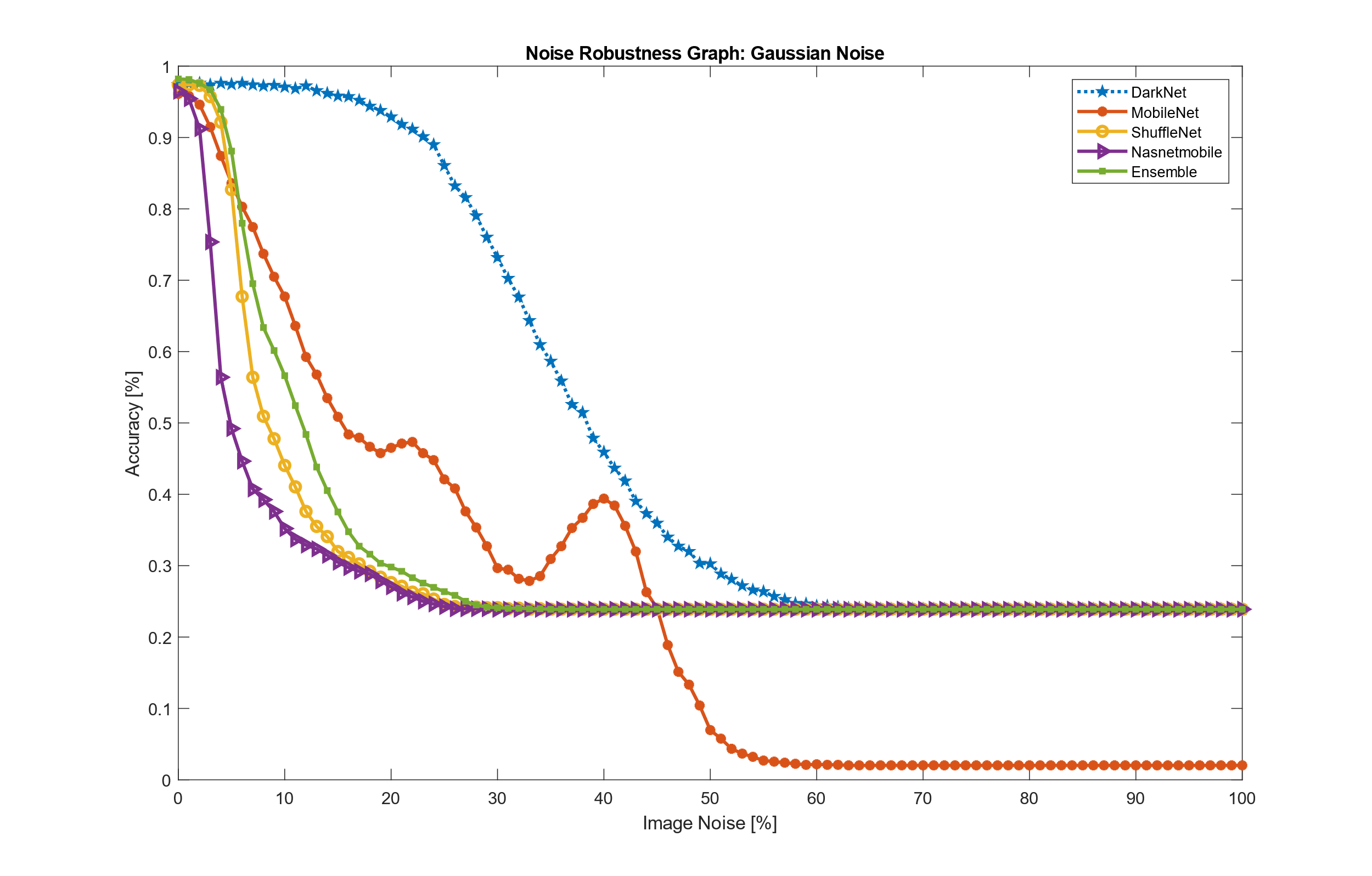** | E)  **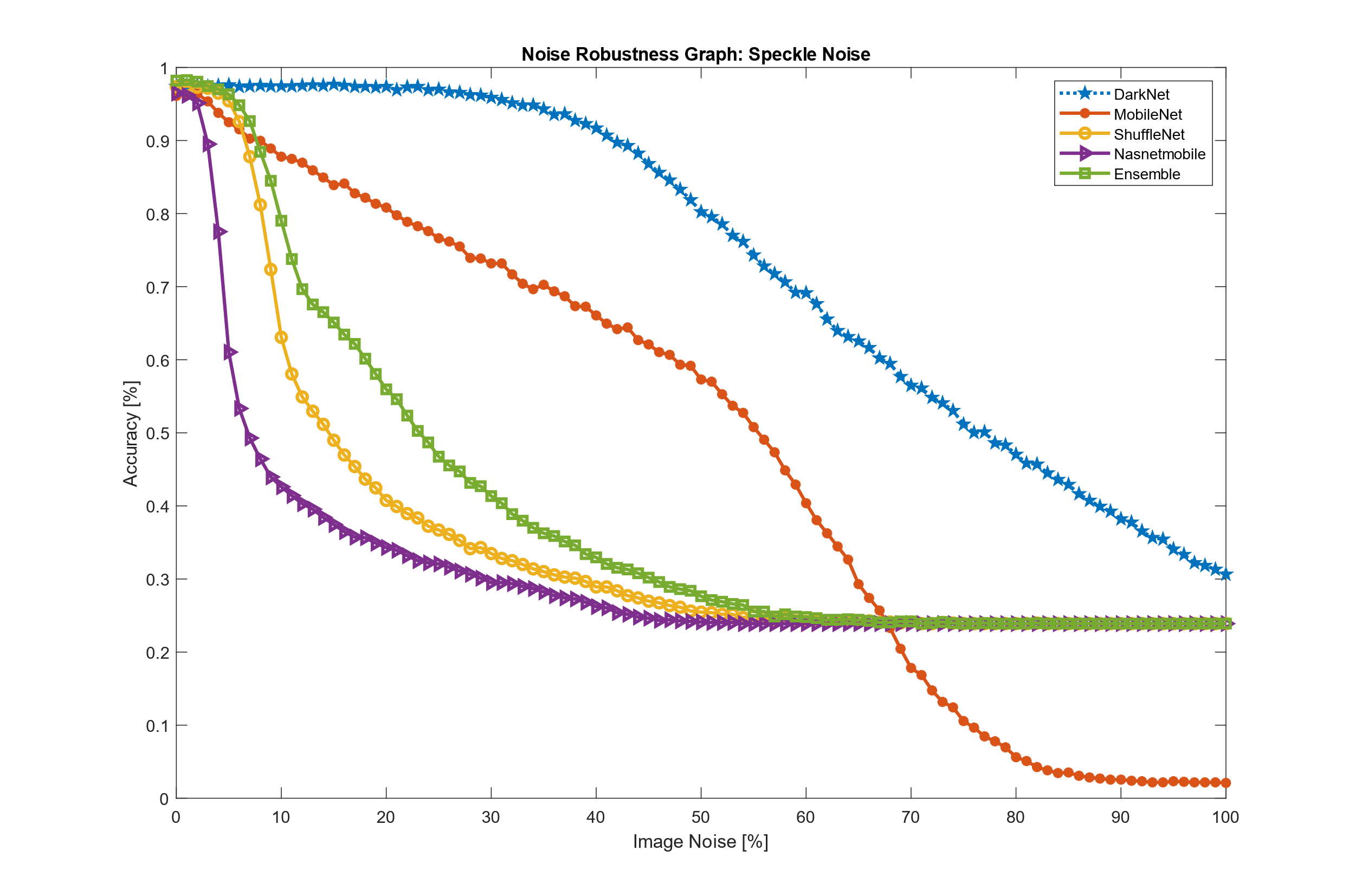** |
| C)  **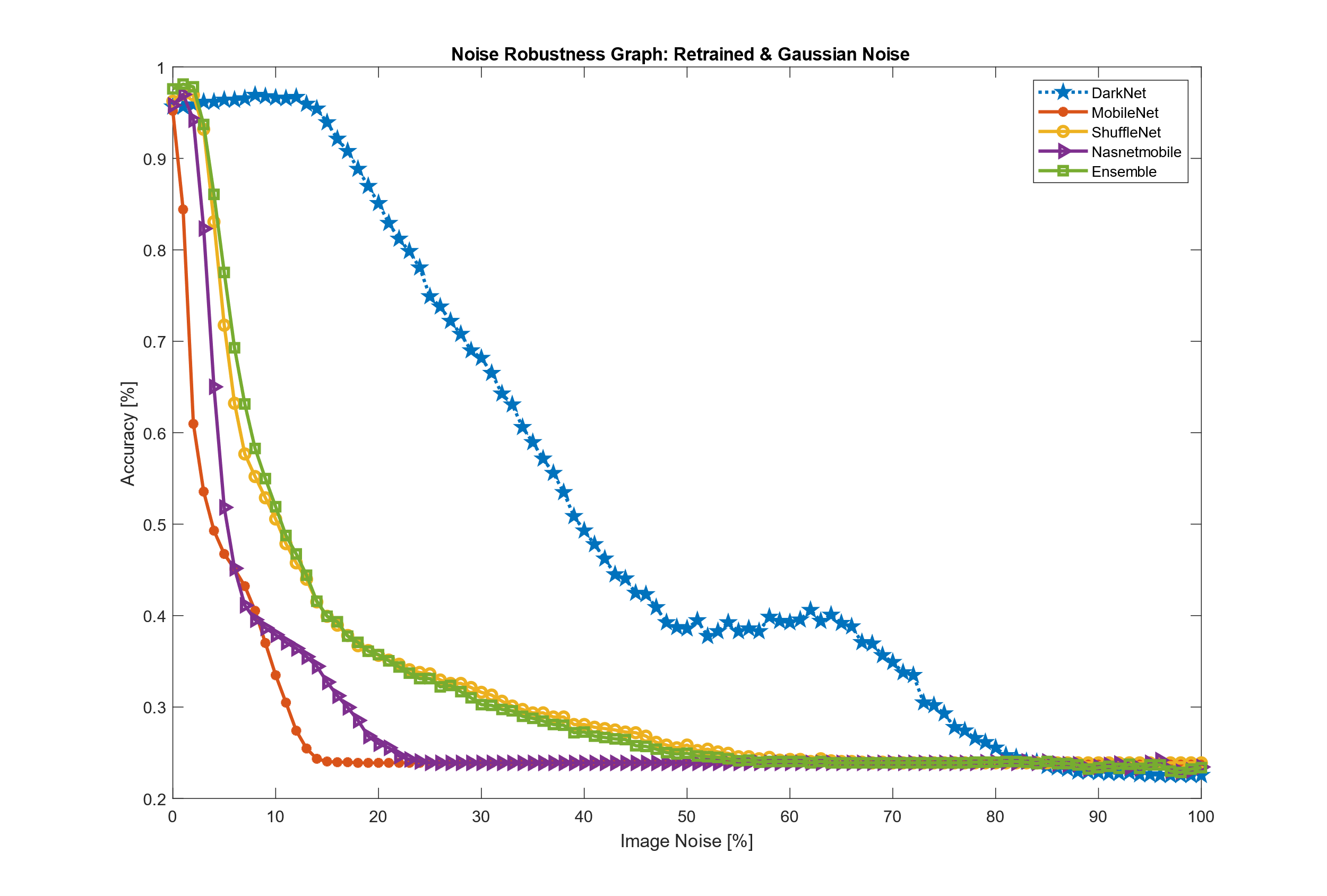** | F)  **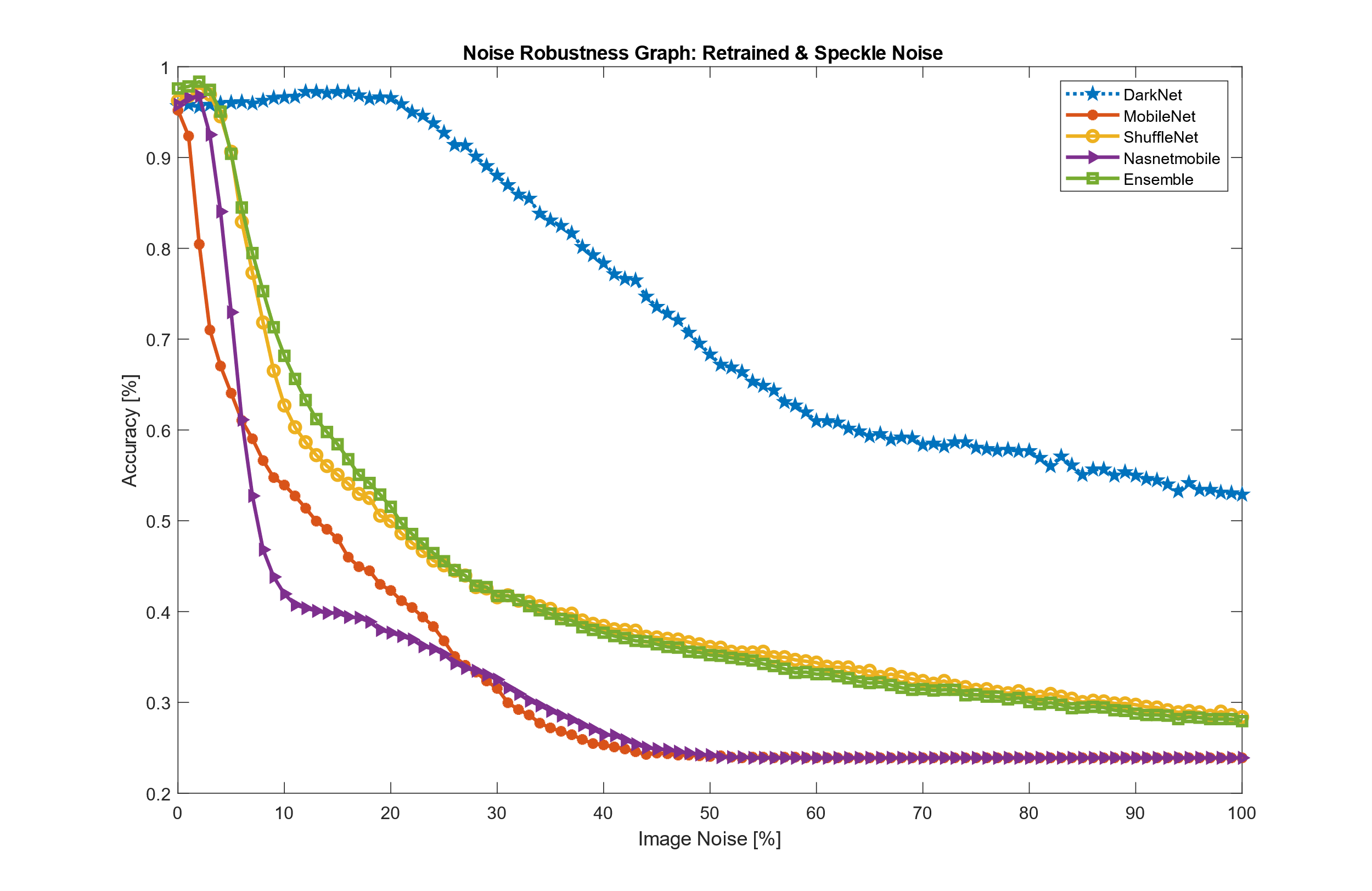** |

**Figure S1:** A) Gaussian noise added 1% at a time from 0% (top left RBC) to 100% (bottom right RBC) to the first RBC in the MH validation set. B & C) Graphs showing the overall accuracy of the original (B) & retrained (C) models for each 1% increase in Gaussian noise added to the MH validation set. D) Speckle noise added 1% at a time from 0% (top left RBC) to 100% (bottom right RBC) to the first RBC in the MH validation set. E & F) Graphs showing the overall accuracy of the original (E) & retrained (F) models for each 1% increase in speckle noise added to the MH validation set.

# **Morphological Heterogeneity Dataset: Comparing the Raw to Processed Images**

**Figures S2** and **S3** depict image 300 from Unit 3, Week 8, & Run 3 in the MH dataset. The first image is the raw input, whereas the second image has superimposed outputs from the preprocessing module of the framework.

**Figure S2**


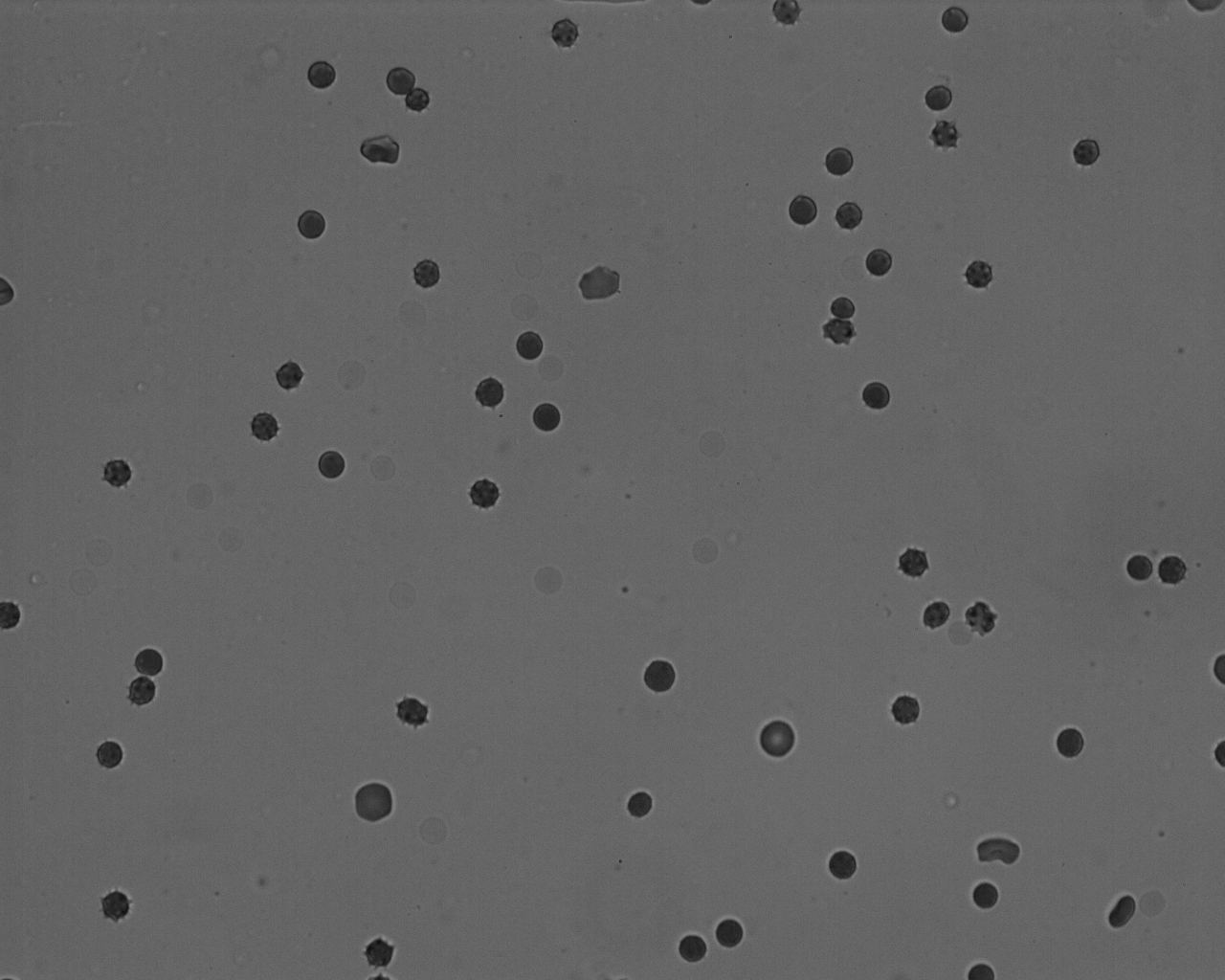


**Figure S2:** An unprocessed sample from the MH dataset (Unit 3, Week 8, Run 3, Image 300).

**Figure S3**


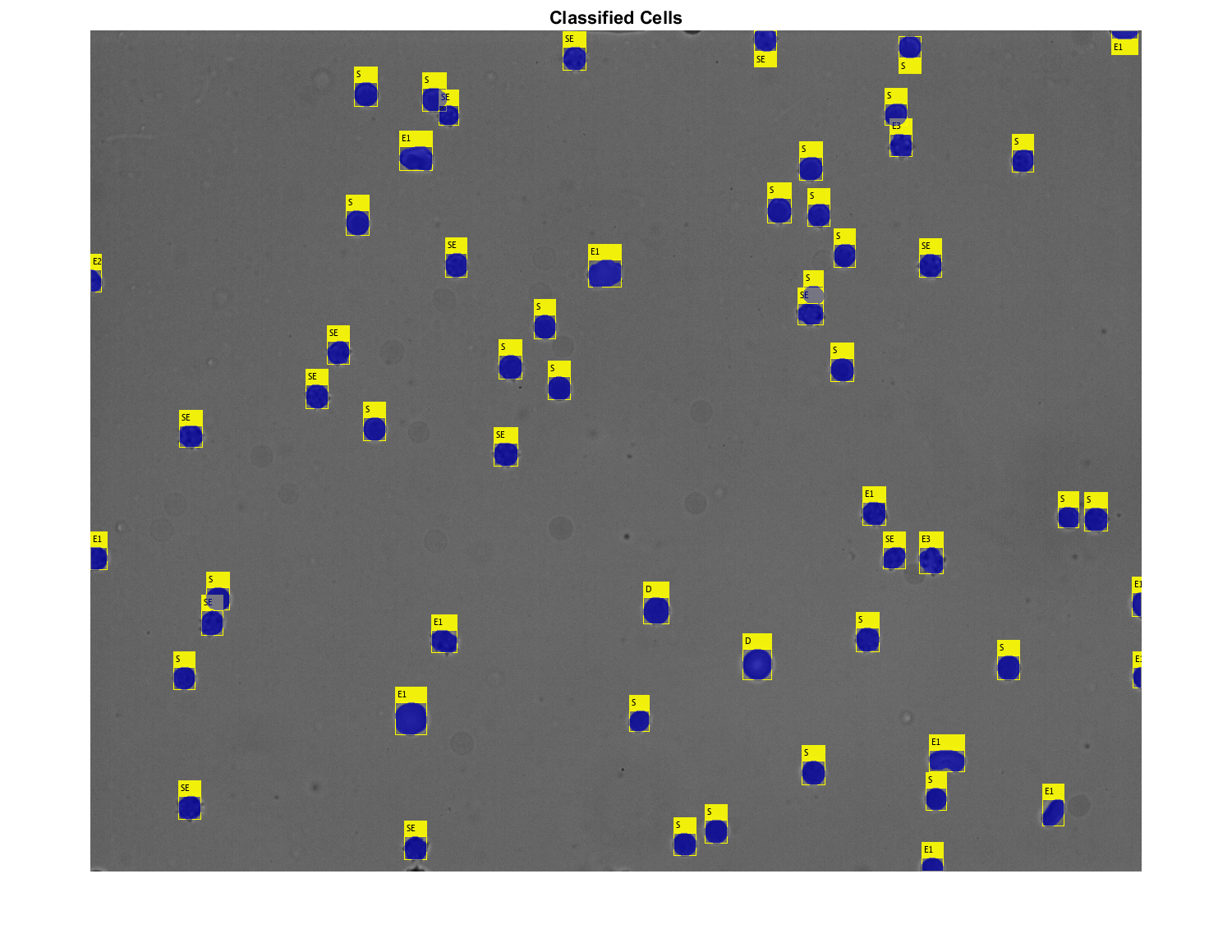
**Figure S3:** A sample from the MH dataset (Unit 3, Week 8, Run 3, Image 300) that has undergone segmentation (adaptive thresholding) and classification with the trained deep ensemble. Note that adaptive thresholding was effective at avoiding numerous ghosts RBCs in the background.

# **Morphological Heterogeneity Dataset: Data Cleaning**

Cleaning occurs in two phases, a low- and high-resolution phase. Both phases make extensive use of normality testing and random RBC image sampling. The primary difference between these phases is that the low-resolution phase cleans the raw image data by removing statistical outliers, whereas the high-resolution phase relies on a trained Darknet-19 classifier to separate good from bad standardized and upscaled (227x227) RBC images.

## **Low-Resolution Data Cleaning**

After gathering all RBCs and their associated data, we create a table that lists all cells. Then we add a diameter column by algebraically rearranging the formula for the area of a circle. We also apply a conversion factor of 0.2193 [um/px] to change pixels to micrometers. Next, we examine and clean the data through normality testing, random RBC image sampling, and the pruning of outliers using various metrics, such as the standard deviation of image height & width, bounding box locations, and average diameter standard deviation.

The distributions of the low-resolution effective diameters were first examined through a box plot (**Figure S4A**), fitted histograms (**Figure S4B**), Q-Q plots (**Figure S4C**), and normal probability plots (**Figure S4D**). These provide evidence indicating a deviation from normality and a large presence of statistical outliers influencing each morphology’s effective diameter distribution.

Cleaning began by removing duplicate images from each Unit-Week-Run. Here, we iterated through the units, weeks, and runs via a nested for-loop and removed individual run duplicates by separating unique bounding box arrays from copies. To verify the removal of duplicates, we randomly sample from both the duplicate and unique subsets, as shown in **Figure S5**. Looking at the unique subset of random samples, one can see the successful removal of duplicates. However, these random samples show misleading RBC data, such as segmentation errors, partially visible cells, and poorly oriented RBCs.

Next, we analyze each morphology class from the unique subset by bounding box height, width, and aspect ratio to judge standard deviation thresholds for each category. Using another nested for-loop, we iterate through morphology labels and then standard deviation values of 1 through 4. For each standard deviation value, we acquire random samples (n = 992) of RBC images below that height and width and equal to and above. Also, we capture RBCs with aspect ratios within and outside the standard deviation. Through visual inspection of these random samples, we chose a standard deviation threshold for each metric within a morphology class. For example, E1 had a threshold of one standard deviation for height & width but a standard deviation of two for aspect ratio because random samples with standard deviations above these values did not sufficiently remove errors while preserving correct images. In contrast, E3 had a global standard deviation threshold of three for all metrics because this was the point where the need to extract errors outweighed the desire to preserve healthy data. To illustrate, **Figure S6A-B** shows E1 outside and within two standard deviations of bounding box aspect ratios, and **Figure S6C-D** depict E3 outside and within three standard deviations of their aspect ratios.

After removing errors based on their bounding box height, width, and aspect ratio standard deviation thresholds, we repeat the process for effective diameter outliers. In short, we capture random samples from each morphology class of RBCs outside various standard deviations of their mean effective diameter and then use these to discern threshold values for each category. For instance, E1 RBCs above or below three standard deviations of their mean effective diameter were removed. In contrast, spherocytes two standard deviations above were typically misclassified as discocytes and so were removed. To verify the success of this approach, we randomly sample (**Figure 7**) from the non-outlier subset after thresholding and found an acceptable level of correct images (~90%).

**Figure S4**

| A)  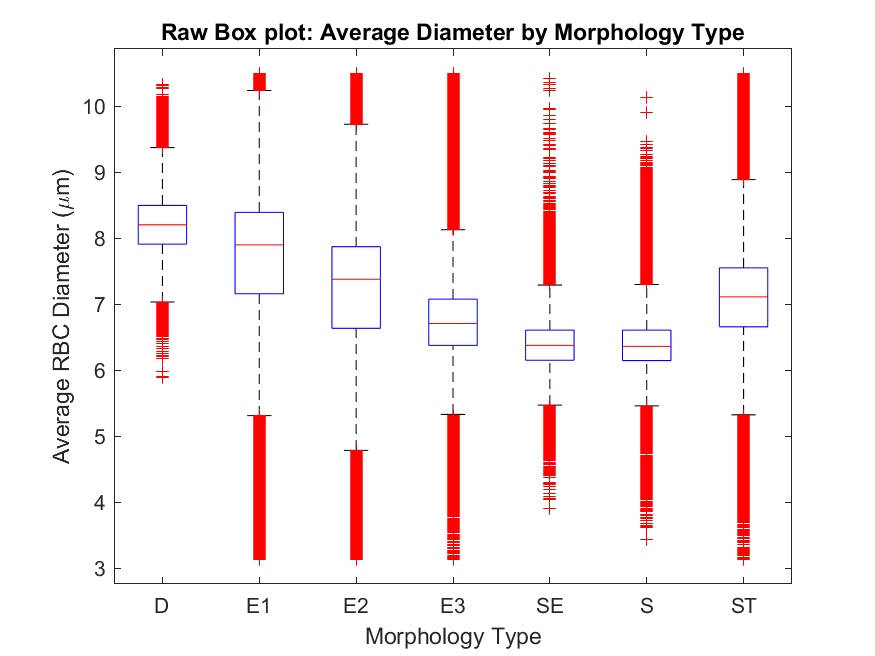 | B)  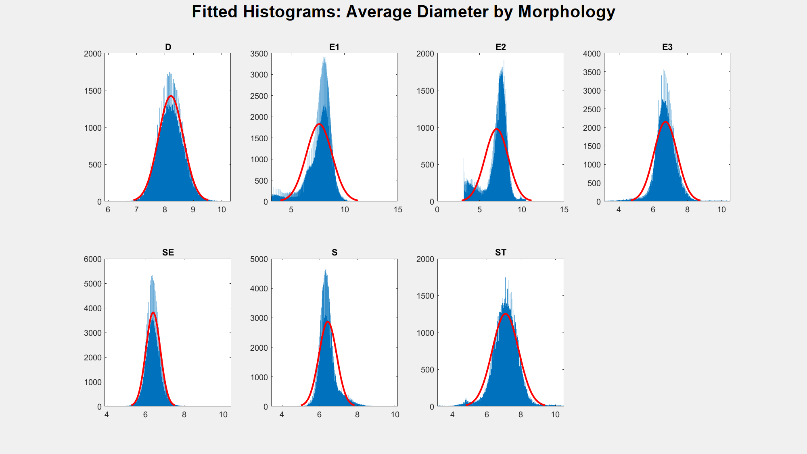 |
| --- | --- |
| C)  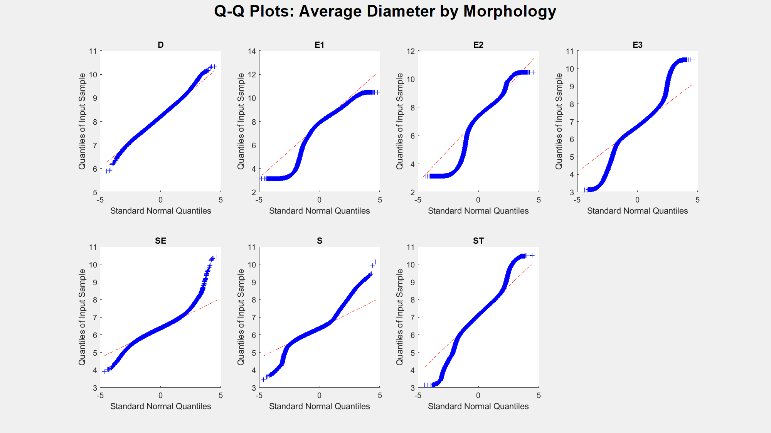 | D)  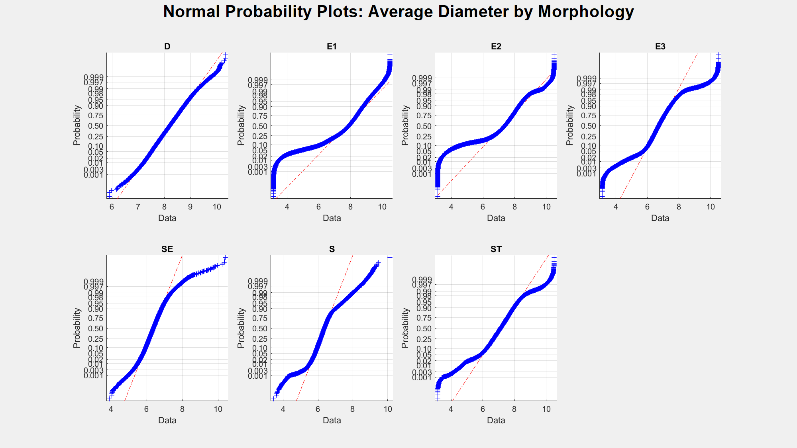 |

**Figure S4:** A) (top-left) The boxplot diagram of the raw effective low-resolution diameter indicating are large number of statistical outliers influencing each morphology’s diameter distribution. B) (top-right) Fitted histograms of each morphology’s effective diameter distribution, showing deviation from normality. C) (bottom-left) Q-Q plots of each morphology’s effective diameter distribution indicating deviation from normality. D) (bottom-right) Normal probability plots of each morphology’s effective diameter distribution, also indicating deviation from normality.

**Figures S5**

| A)  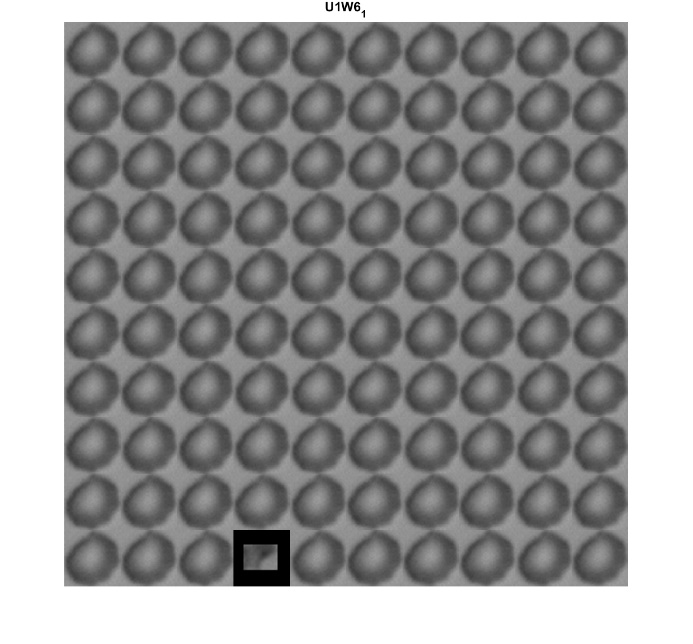 | B)  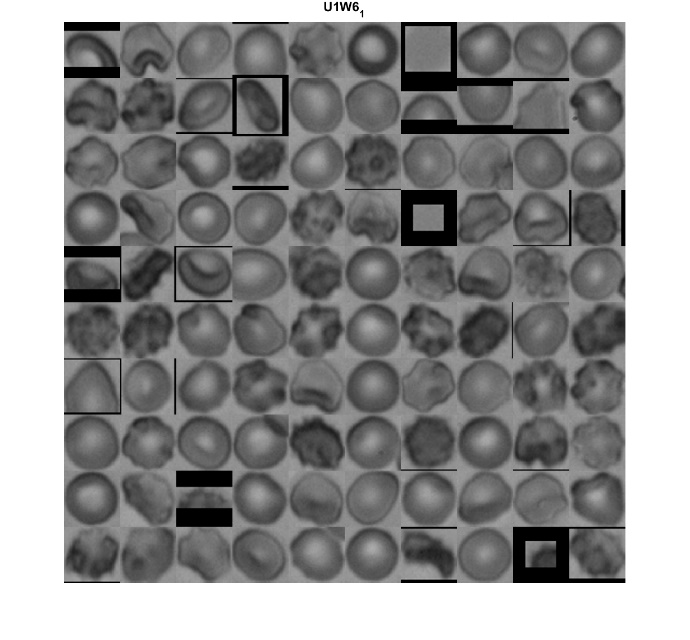 |
| --- | --- |
| C)  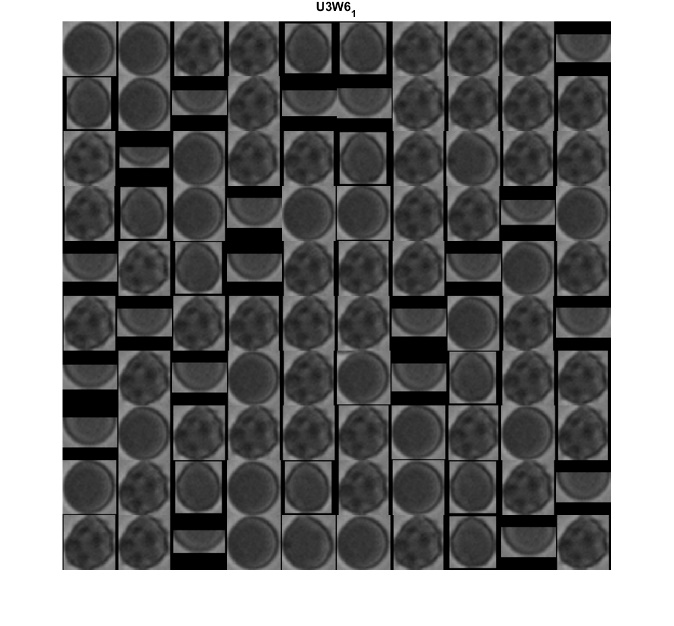 | D)  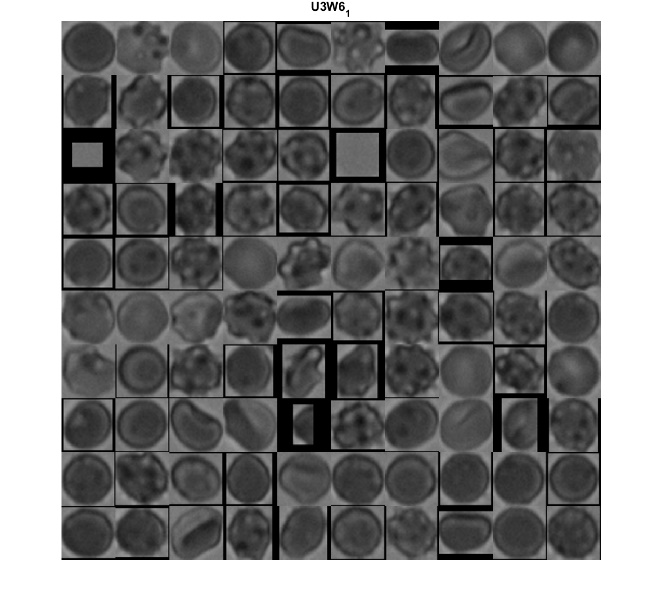 |

**Figure S5:** Random samples from the duplicate (left column) and unique (right column) of two unit-week-runs. The first row is of cropped images from the first run of week six in unit one, whereas the second row shows images from the first run of the eighth week of unit three. Most other unit-week-runs are similar in that duplicates are successfully removed, but errors in segmentation and orientation remain in the unique subset.

**Figure S6**

| A)  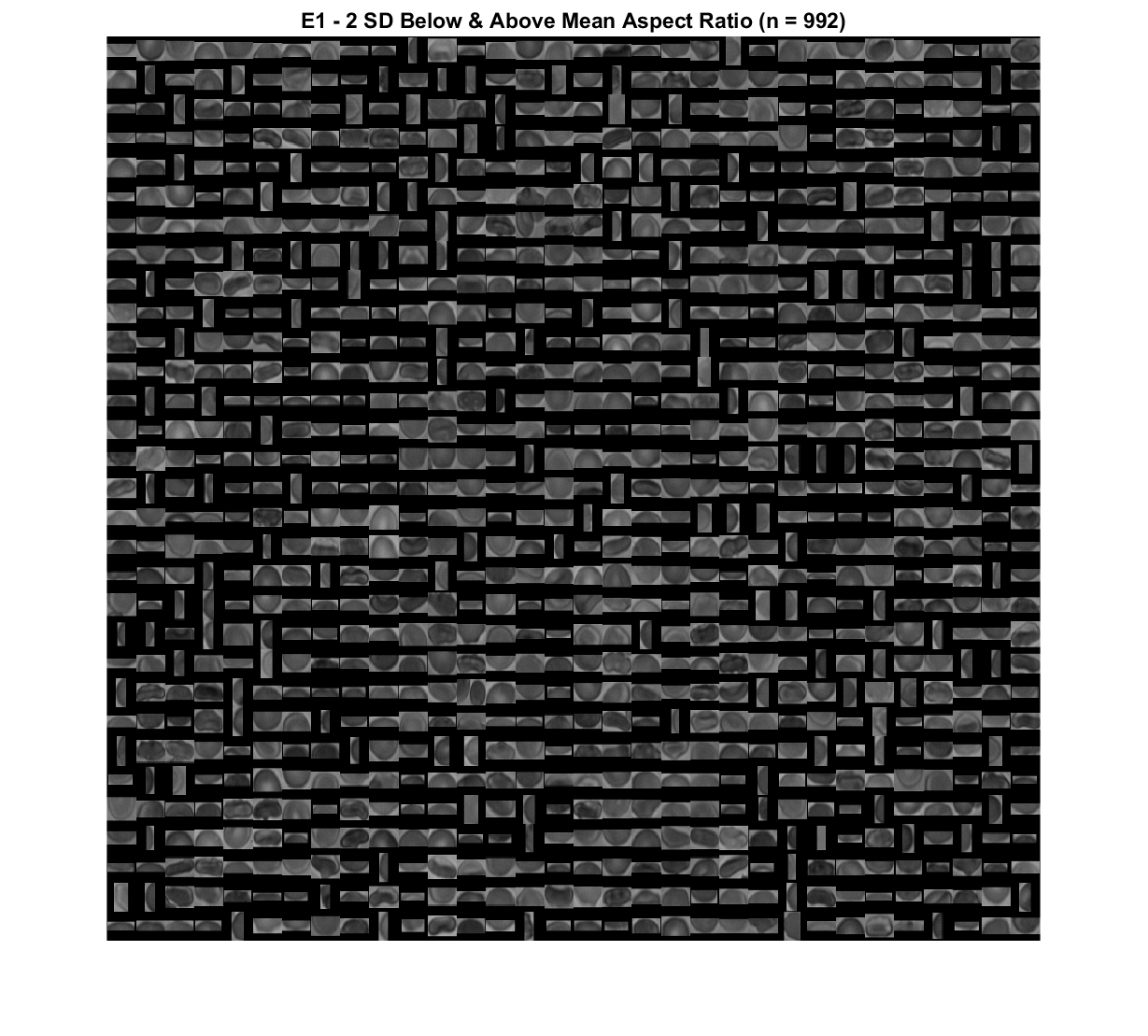 | B)  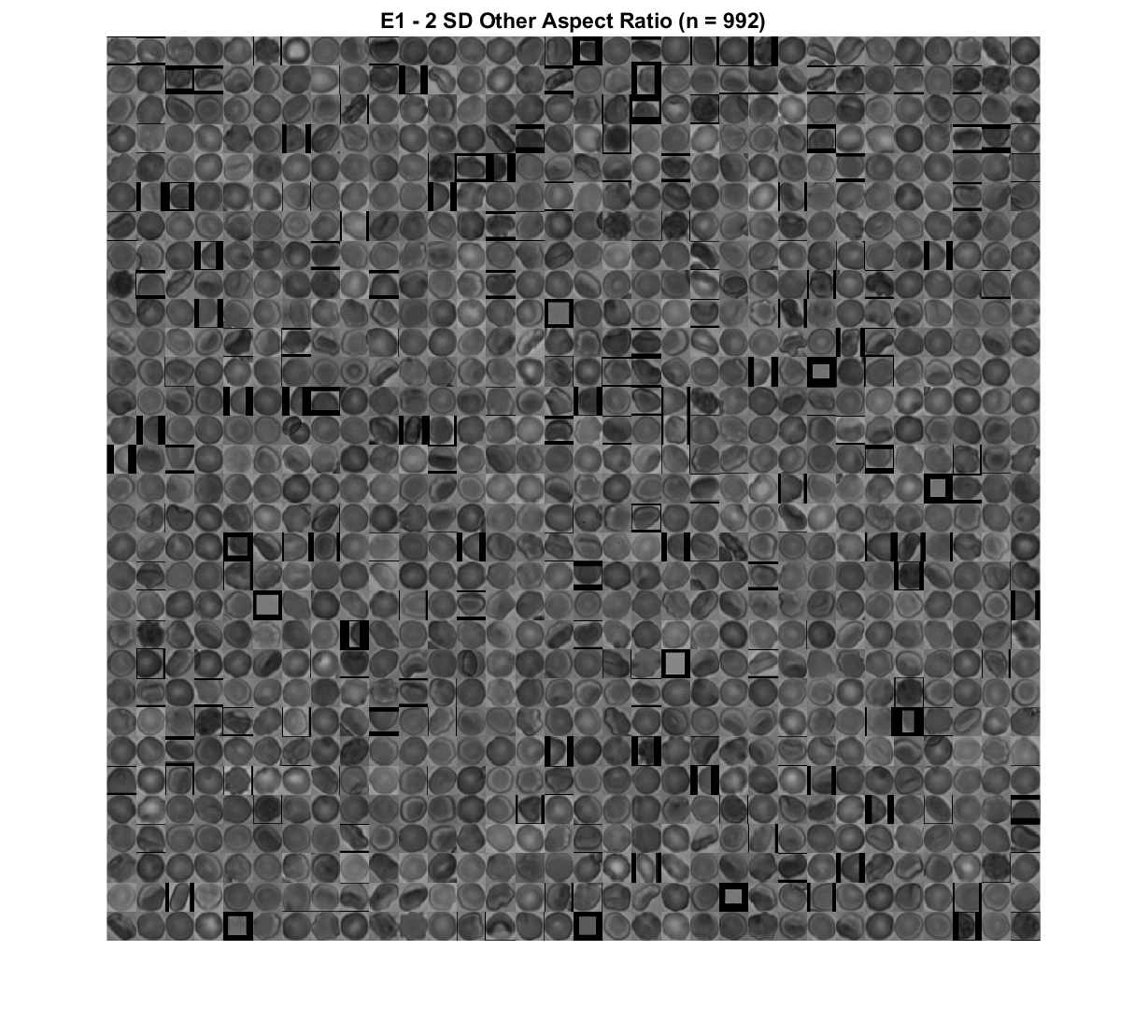 |
| --- | --- |
| C)  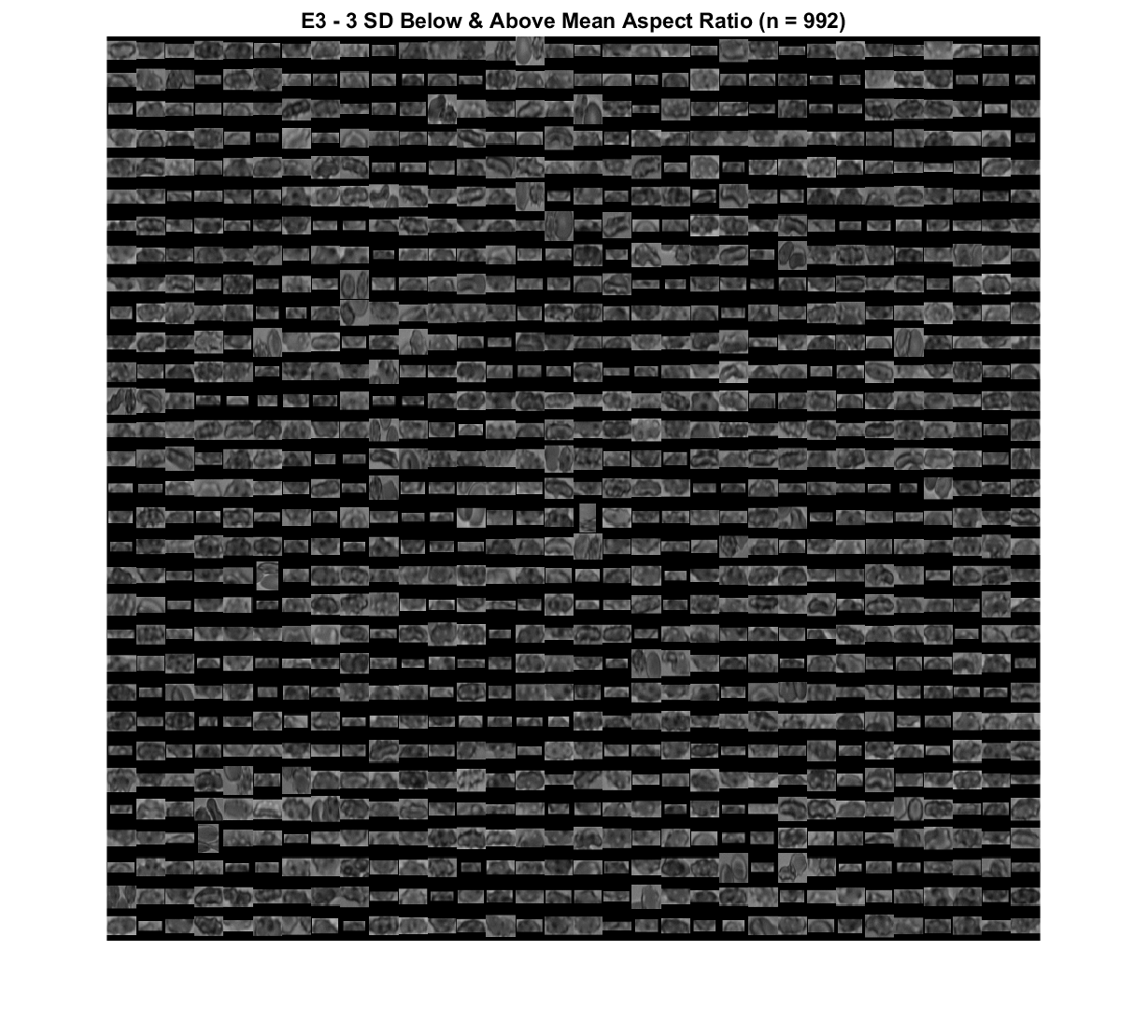 | D)  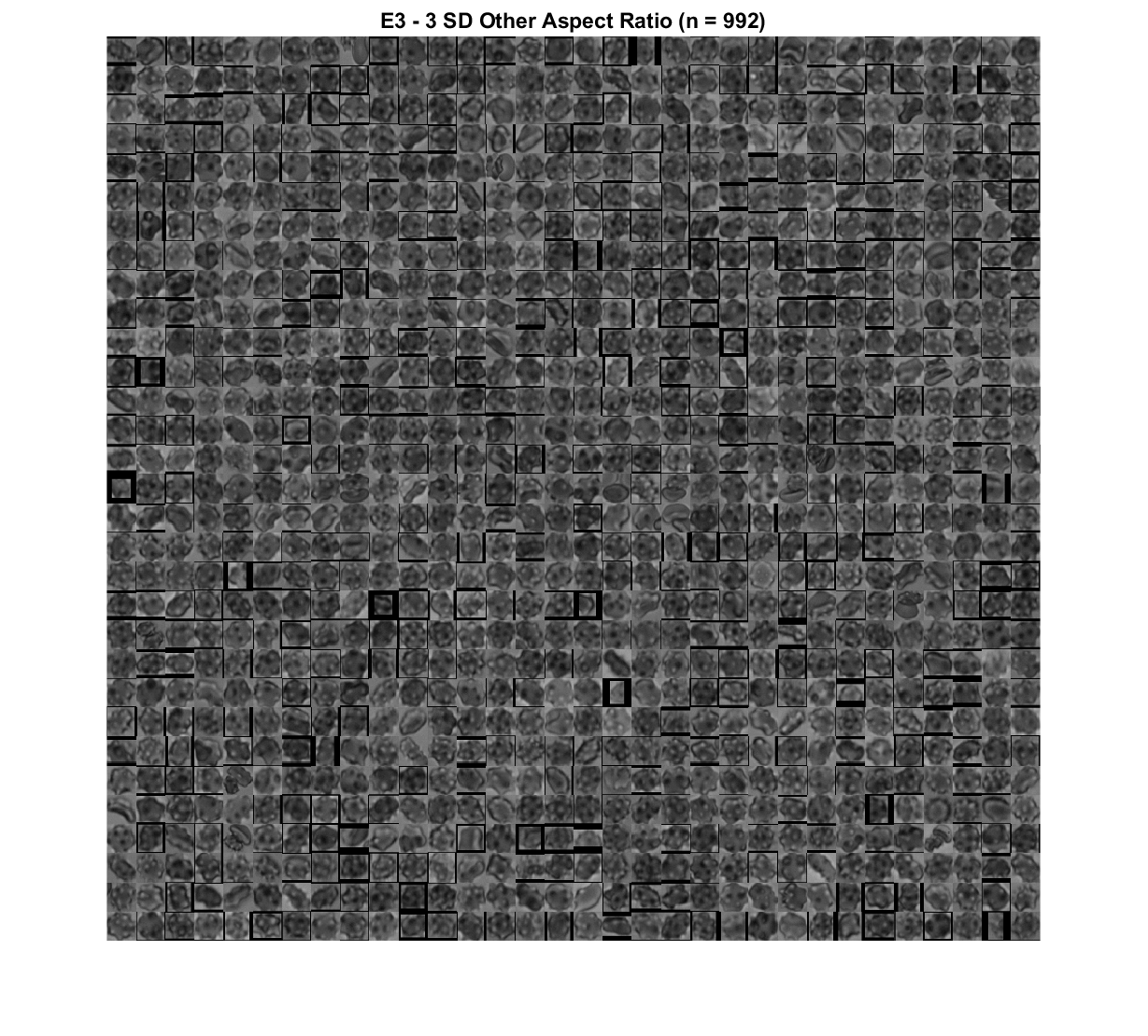 |

**Figures S6:** Random samples of the E1 (top-row) and E3 (bottom-row) bounding box aspect ratios outside (left column) and inside (right column) their threshold standard deviations.

**Figure** **S7**

| A)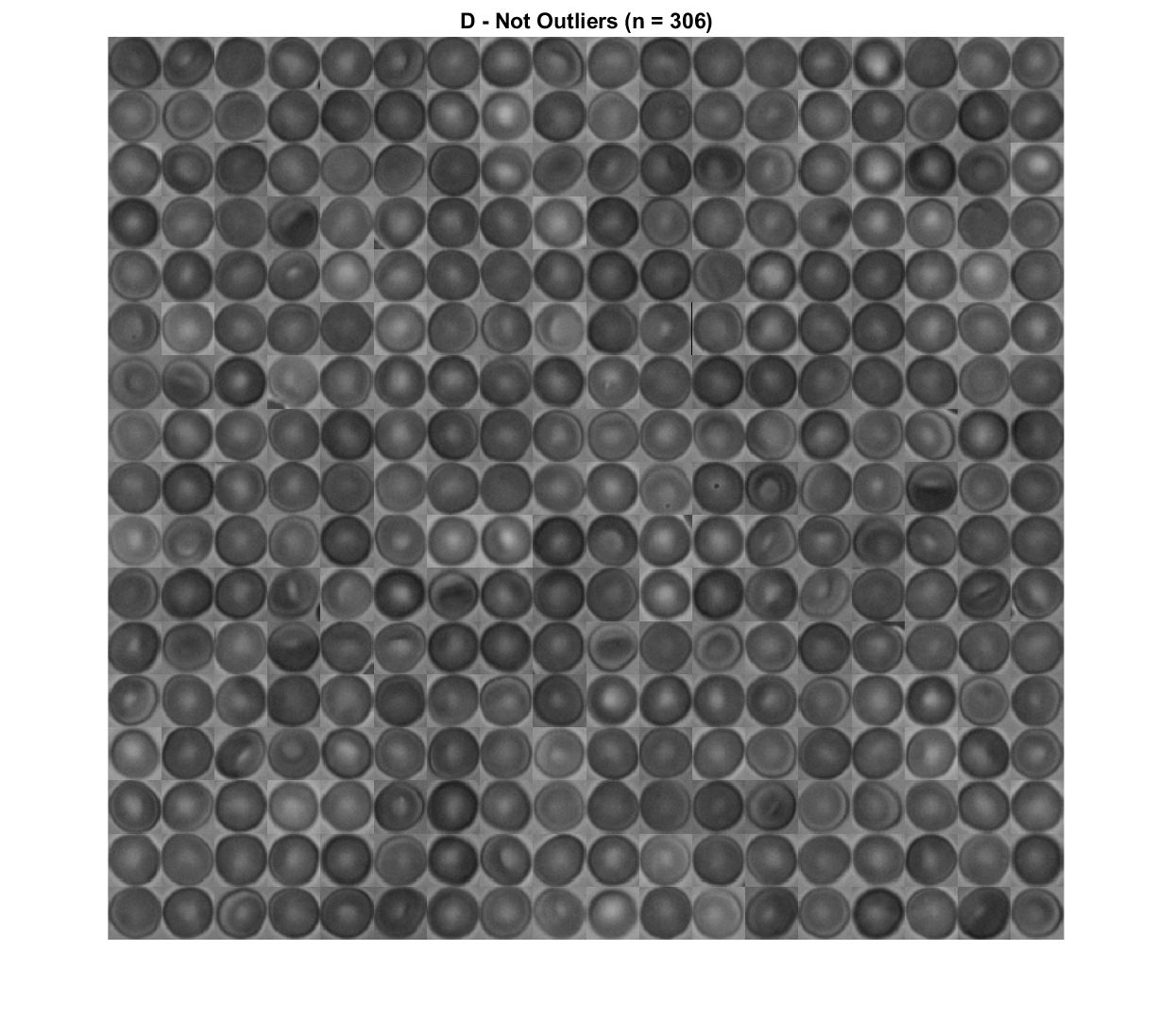 | B)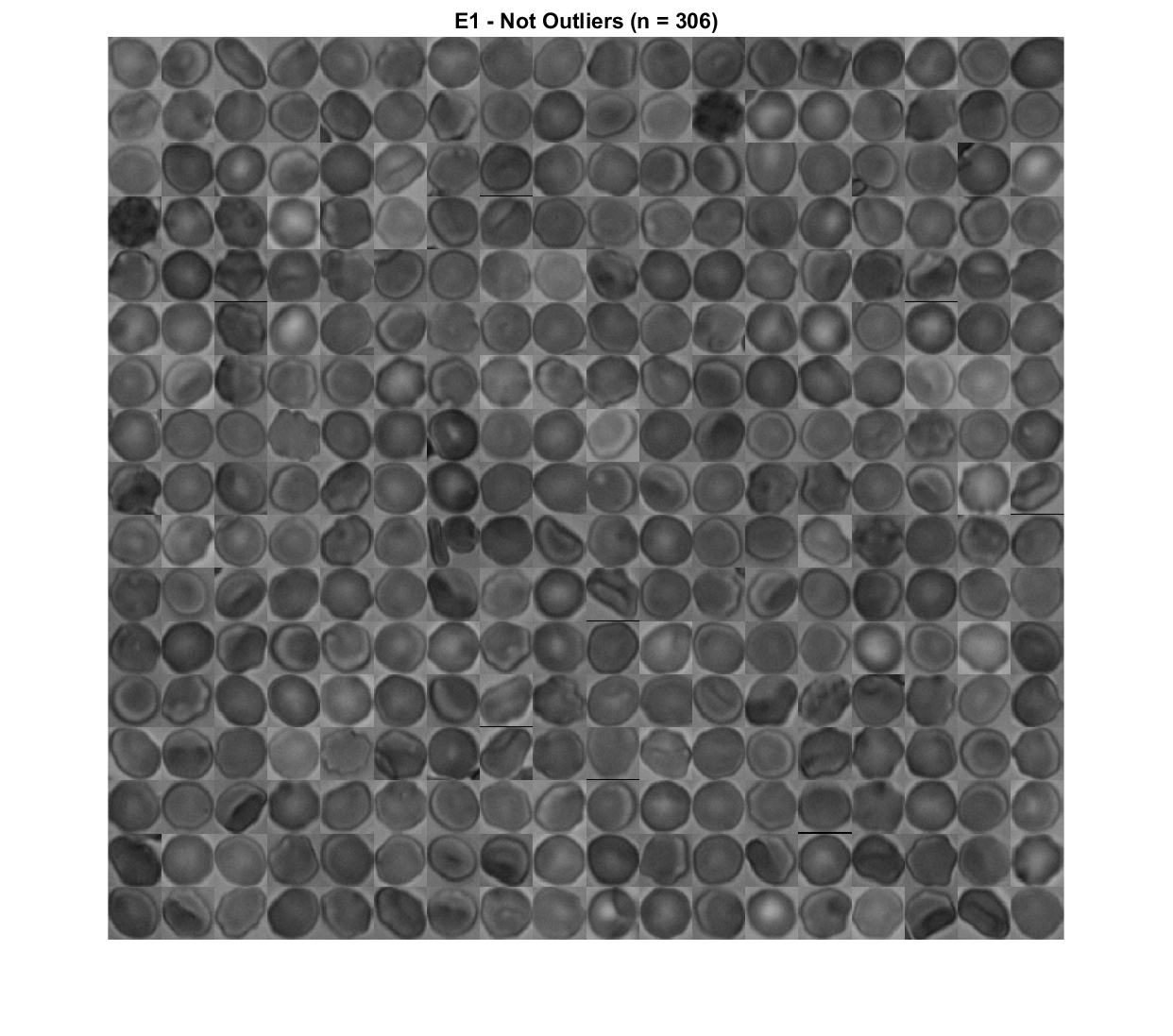 | C)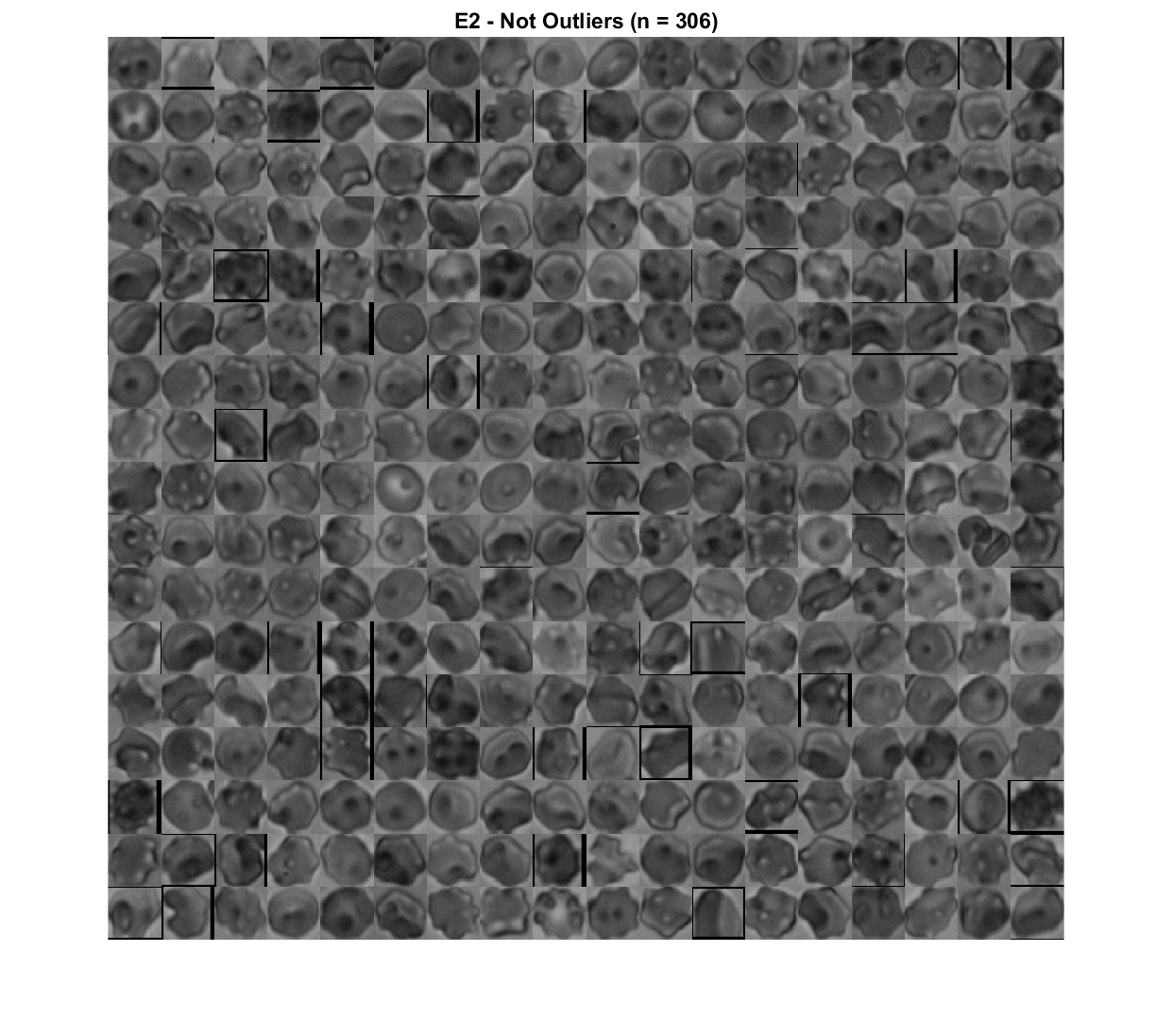 |
| --- | --- | --- |
| D)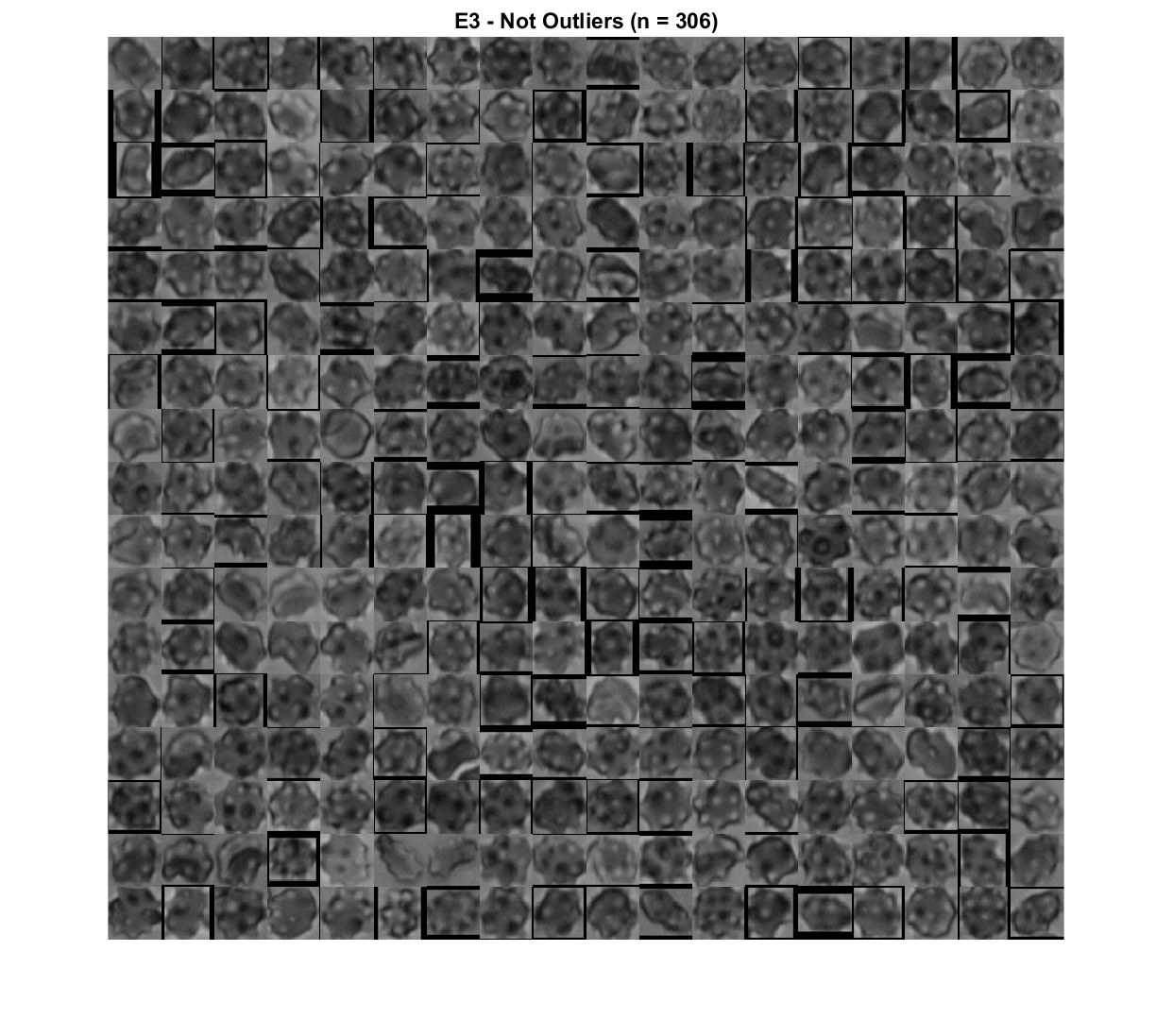 | E)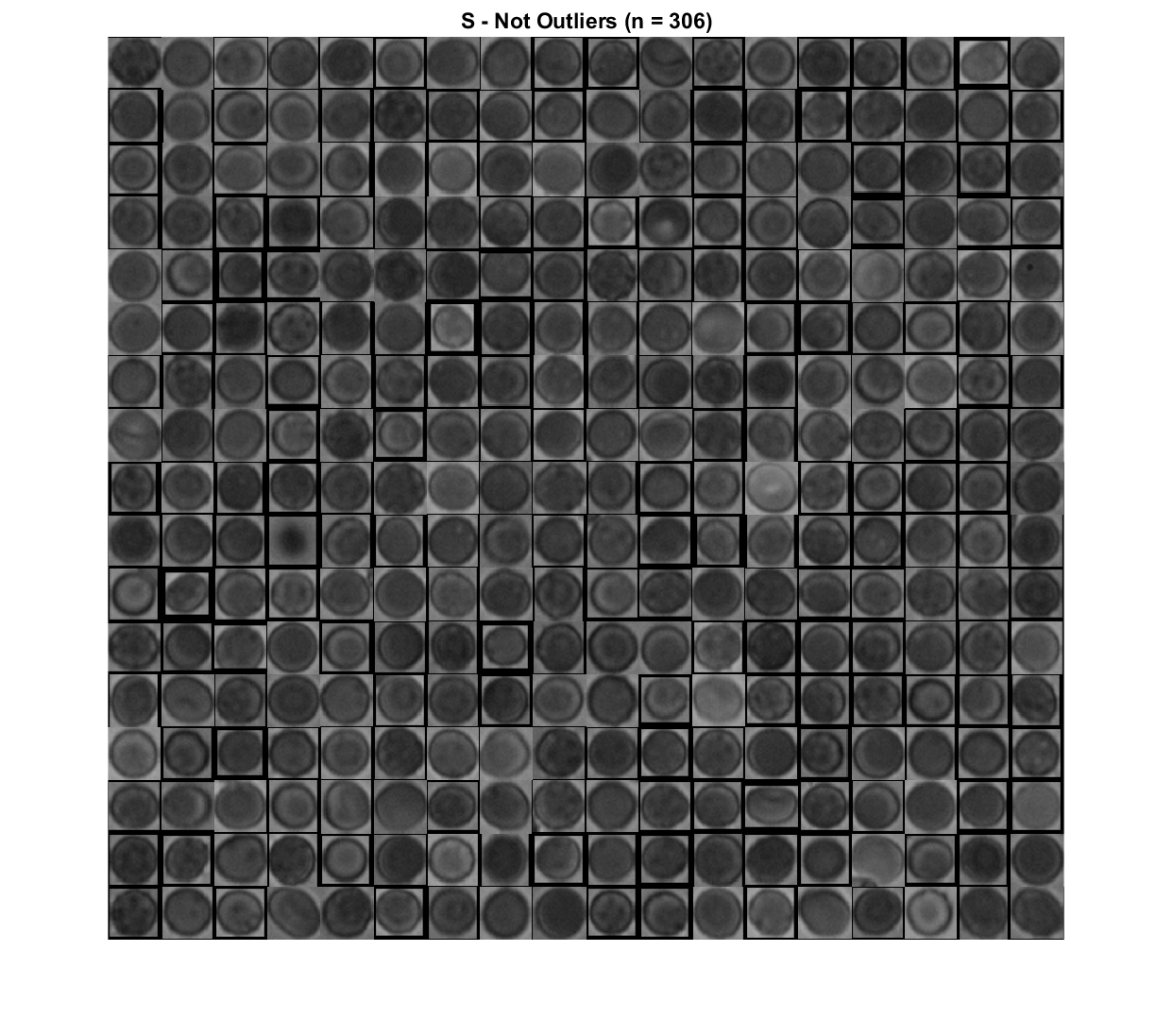 | F) 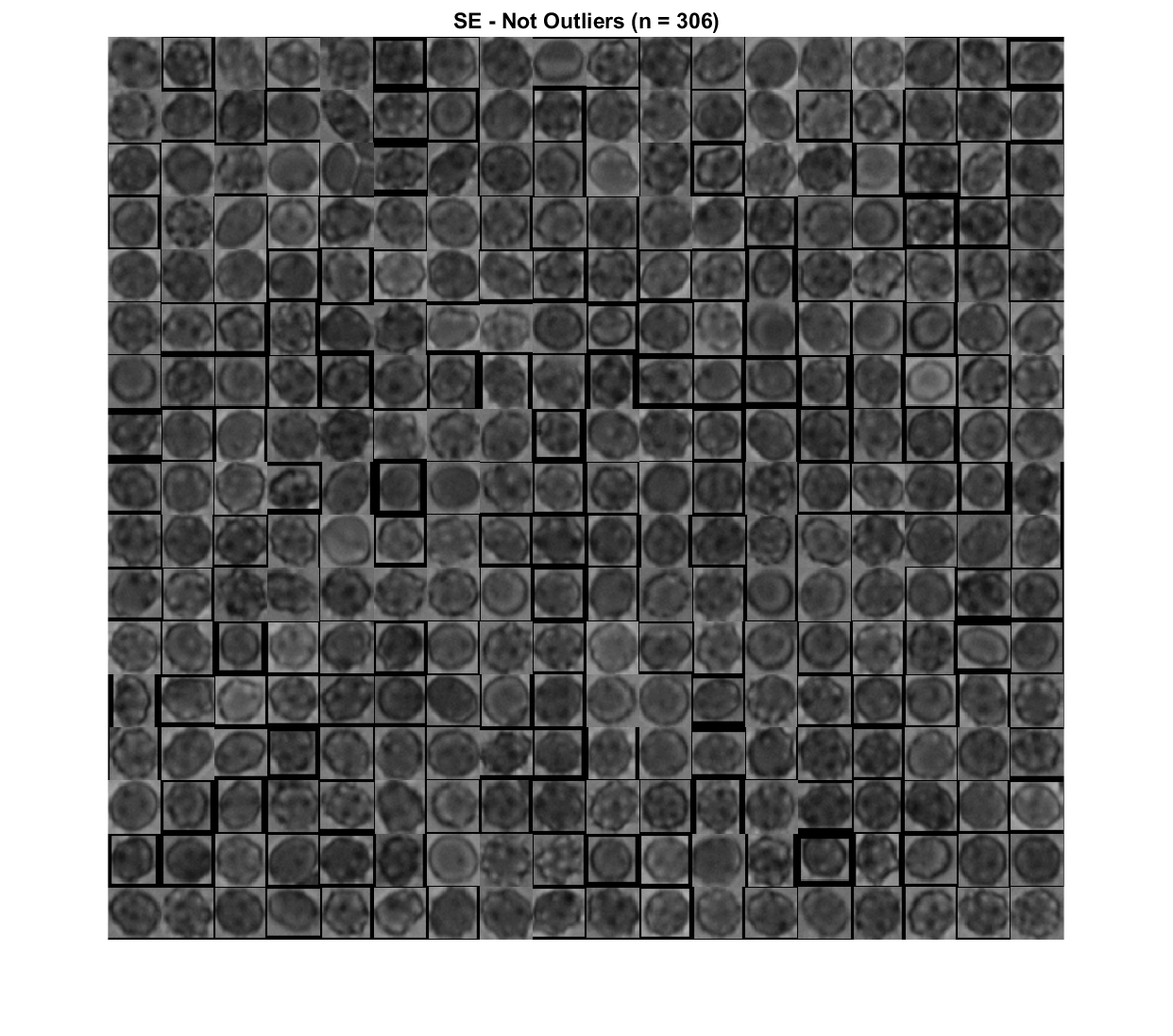 |
| G)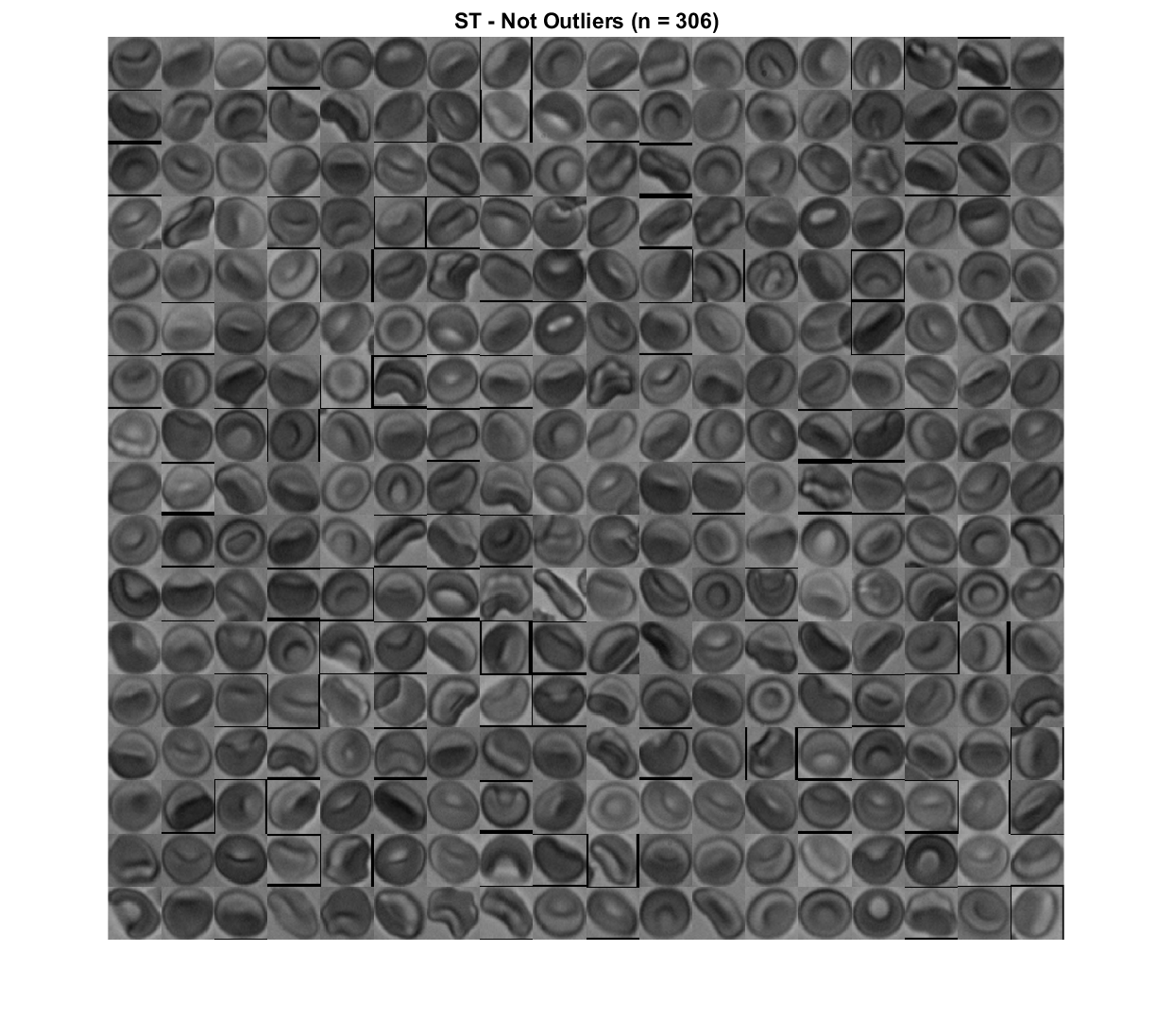 |  | |

**Figure S7:** Three-hundred and six randomly sampled (A) discocytes, (B) stage 1 echinocytes, (C) stage 2 echinocytes, (D) stage 3 echinocytes, (E) sphero-echinocytes, (F) spherocytes, and (G) stomatocytes from the non-outlier subset.

## **High-Resolution Data Cleaning**

Although the **Low-Resolution Data Cleaning** process removed many errors, we wanted to further improve the quality of our effective diameter data. Also, examining the segmentation masks for the low-resolution images revealed a lack of conformity around the contours of each RBC, which is a source of error in our low-resolution diameter calculations.

The high-resolution cleaning process began by centrally positioning, upscaling, and re-segmenting all cells. Using the cleaned low-resolution subset, we iterated through each cell, cropped them from within the larger images, applied their low-resolution binary masks, and pasted them to the center of 51x51 blank images with pixel values set to 220. We then resized these to 227x227, normalized, and re-segmented them using our deep ensemble classifier's segmentation method. New area values were calculated by taking the sum of the high-resolution masks and dividing them by a scaling factor of 19.8112264514. The effective diameter was then calculated as two times the square root of the downscaled area divided by pi. We converted from pixels to micrometers using the same conversion factor of 0.2193.

Once we exported the images, masks, and data, an expert looked through the images and created a binary training set (n=3,743) of good and bad RBCs that were used to train a Darknet-19 binary classifier. Bad RBC images were those with improper orientations, such as discocytes seen from the side, aggregated cells that water shedding did not separate, segmentation errors, and partially visible cells. Using a 10% holdout (n=374) of the training set for model testing, we found an accuracy of 96.5% for the binary classifier (**Figure S8**).

After training and validating the binary classifier, we use it to separate the RBC data into good and bad subsets from which we took random samples. **Figure S9** shows the bad RBCs that were removed from each morphology class.

Based on our initial results from randomly sampling from the good RBC subset, we noticed that spherocytes above two standard deviations from their mean effective diameter were typically misclassified discocytes, so we removed them. Following the removal of spherocytes two standard deviations greater than their mean effective diameter, we took random samples from the good RBC subset within one median absolute deviation from their median effective diameter (**Figures S10A-G**). These confirm the high quality of the effective diameter data.

Finally, we finished the data cleaning process by examining the impact of outliers and the normality of the data through a boxplot (**Figure 11A**), fitted histograms (**Figures 11B**), Q-Q plots (**Figure 11C**), and normal probability plots (**Figure 11D**). Statistical outliers were reduced compared to the raw data, and effective diameter distributions better approximate a normal distribution. Given these data coupled with the random samples, we are confident that our effective diameter statistics for the MH dataset are reasonable and more accurate than our prior work.

**Figure S8**

| A)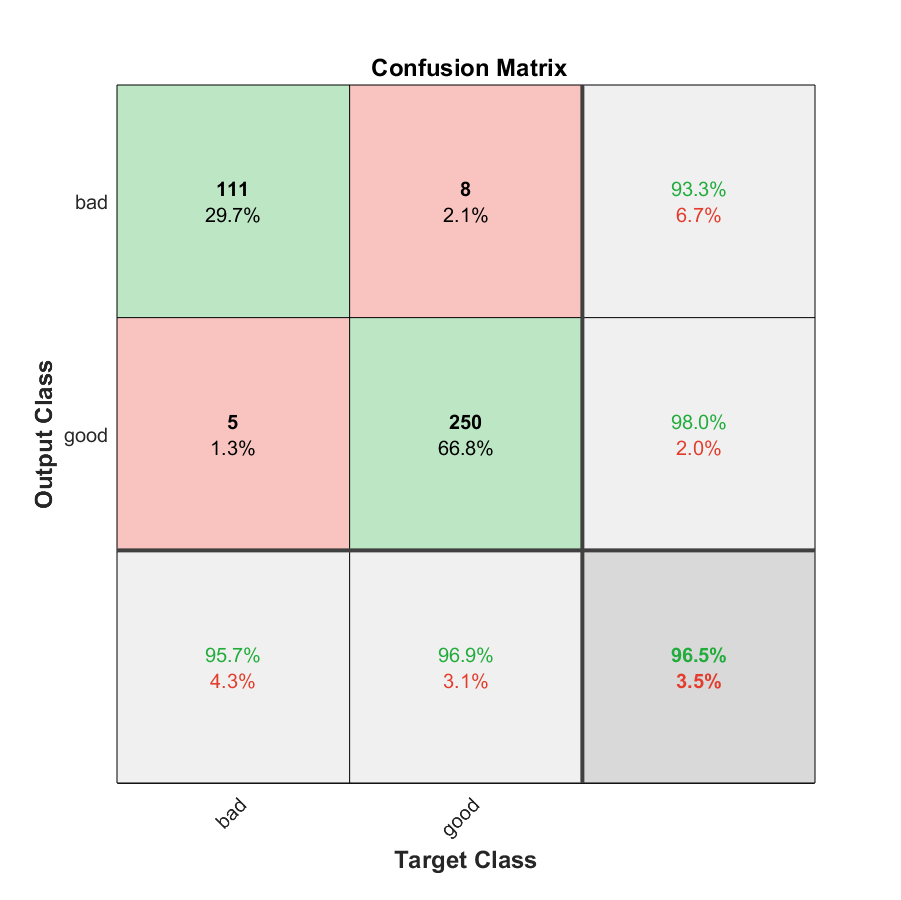 | B)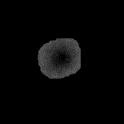 | C)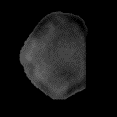 |
| --- | --- | --- |
|  | D)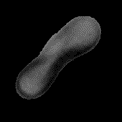 | E)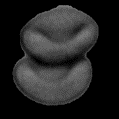 |

**Figure S8:** A) The confusion matrix of our binary classifier to detect bad RBCs. B) An example of segmentation error. C) An example of a partially visible RBC. D) An example of bad RBC orientation. E) An example of aggregate RBCs.

**Figure S9**

| A)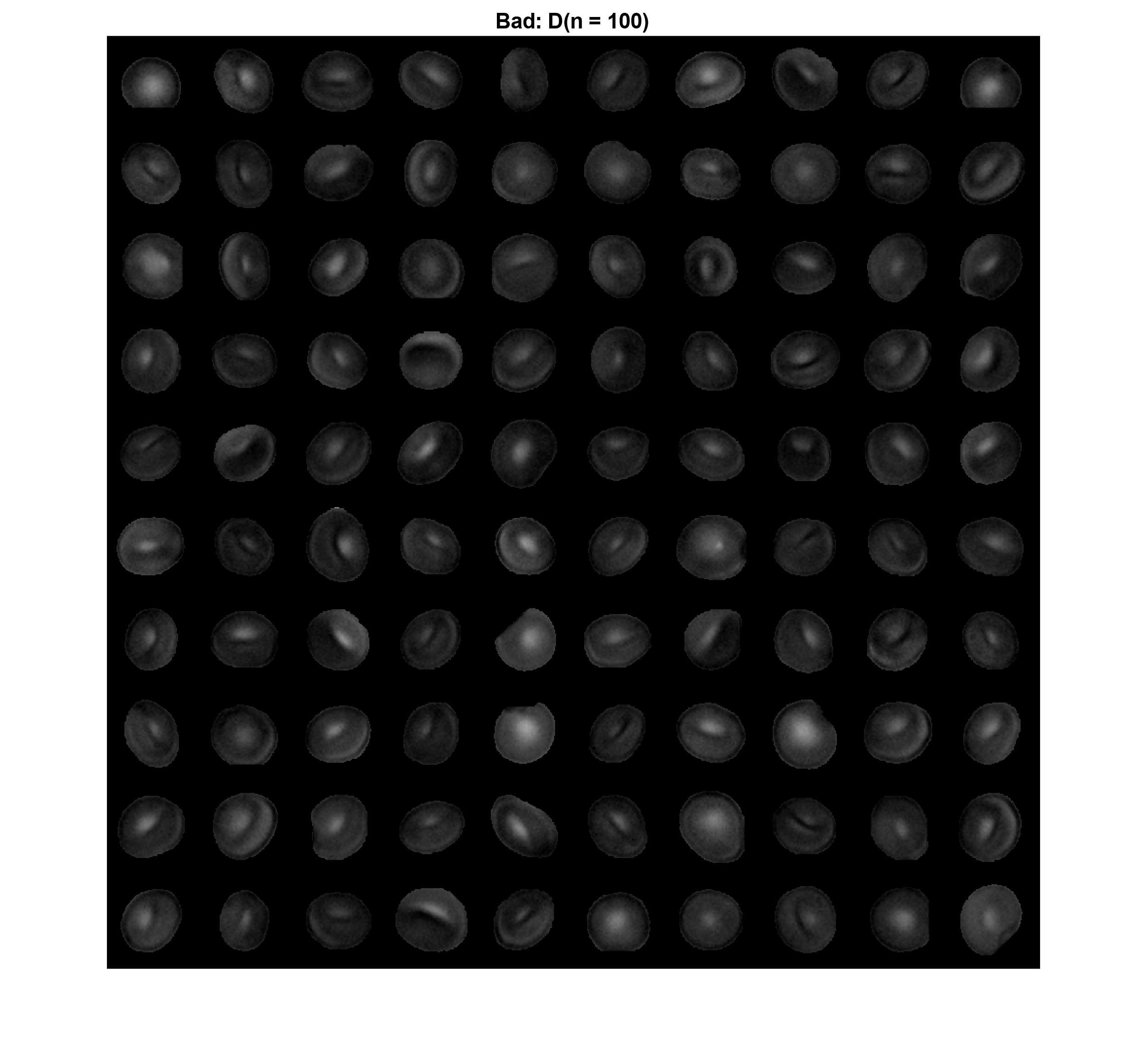 | B)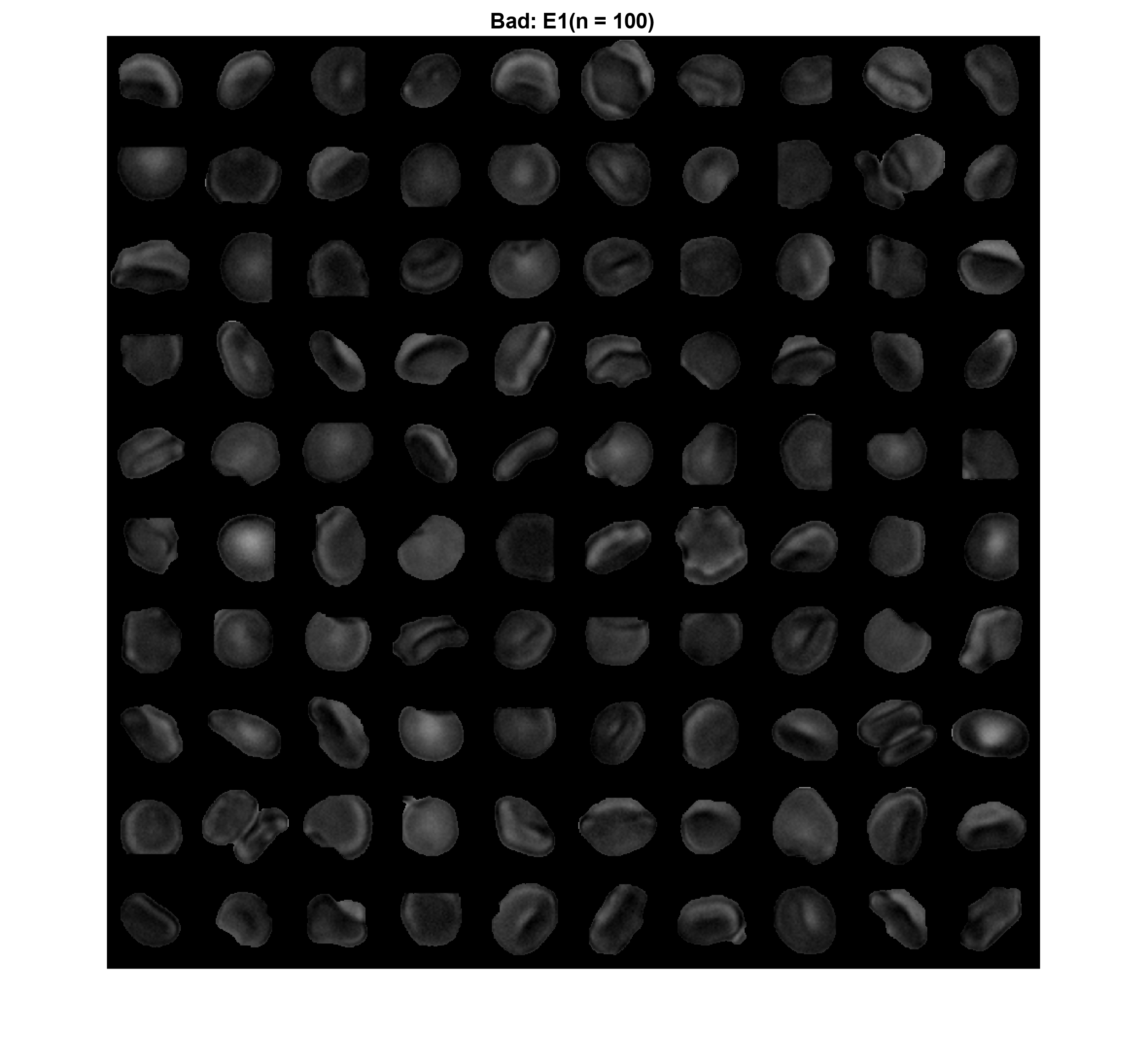 | C)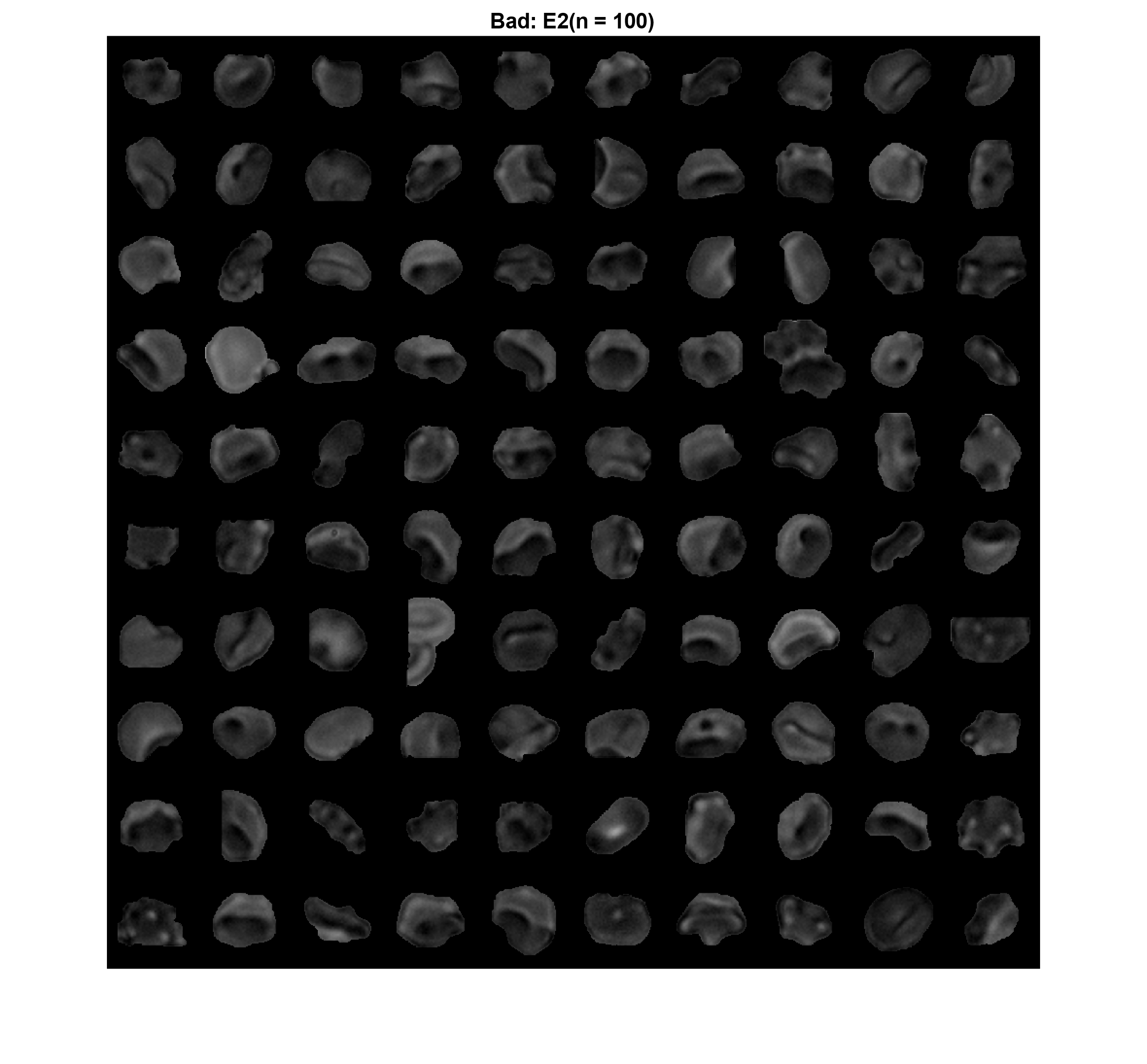 |
| --- | --- | --- |
| D)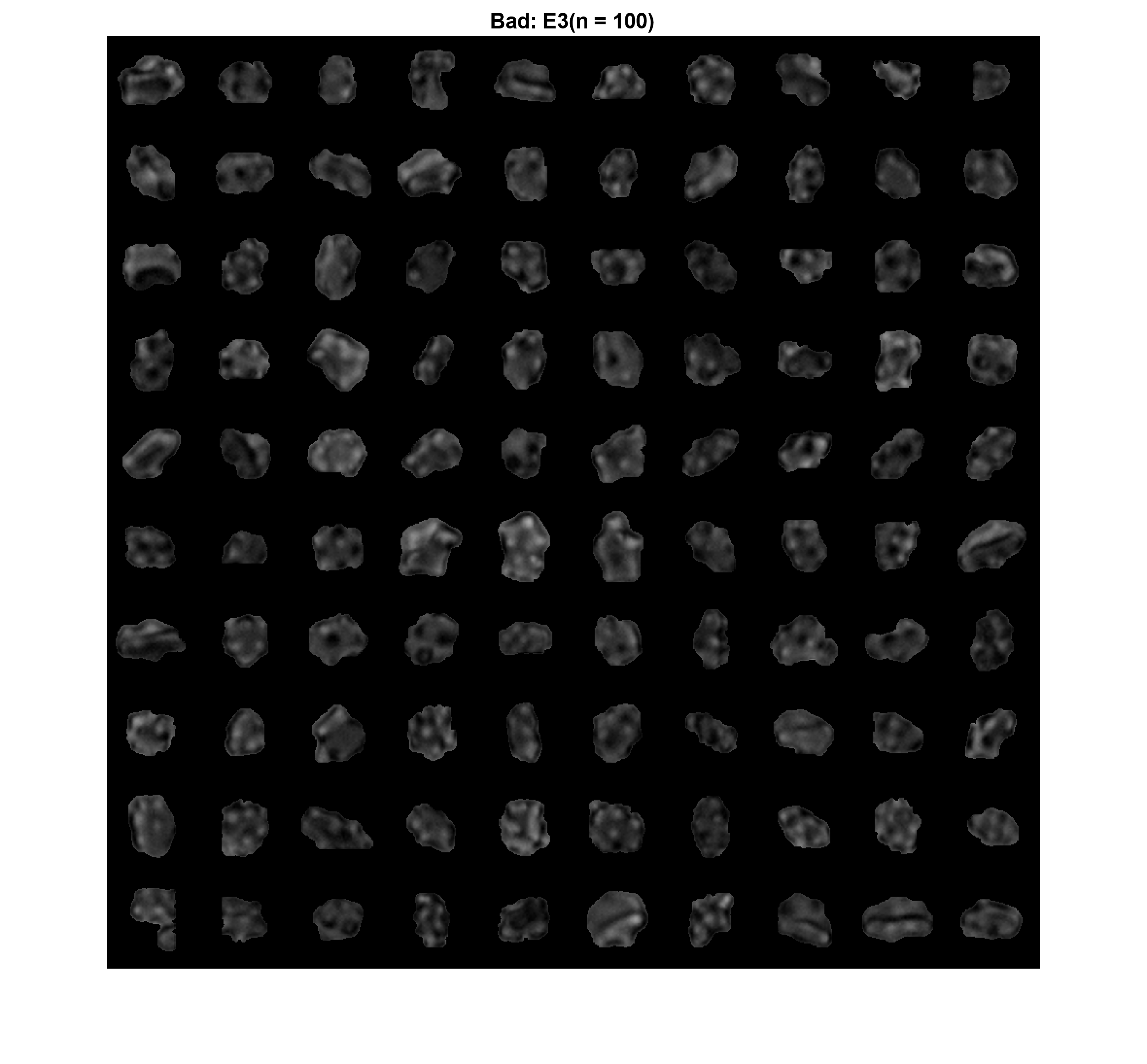 | E)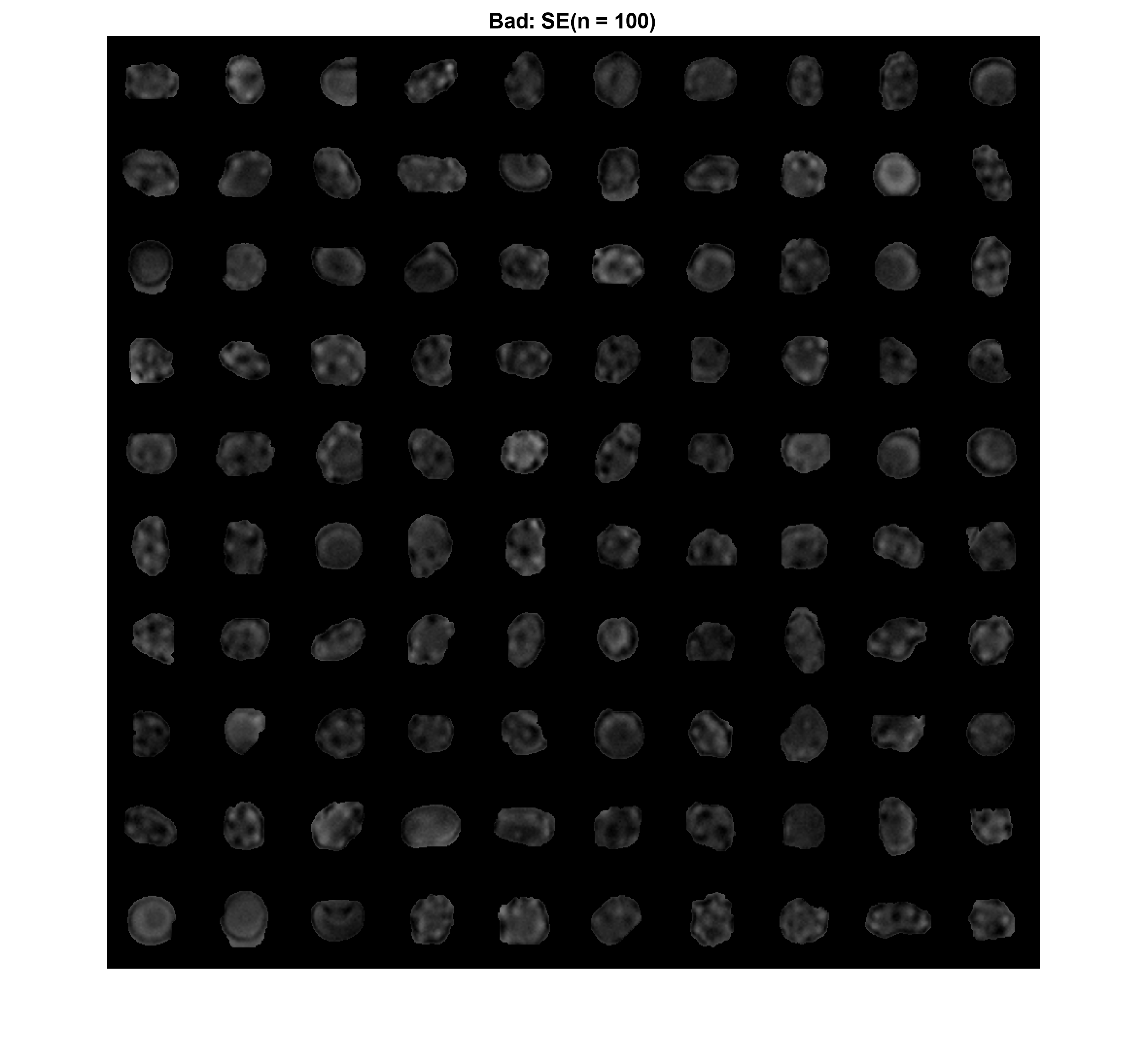 | F)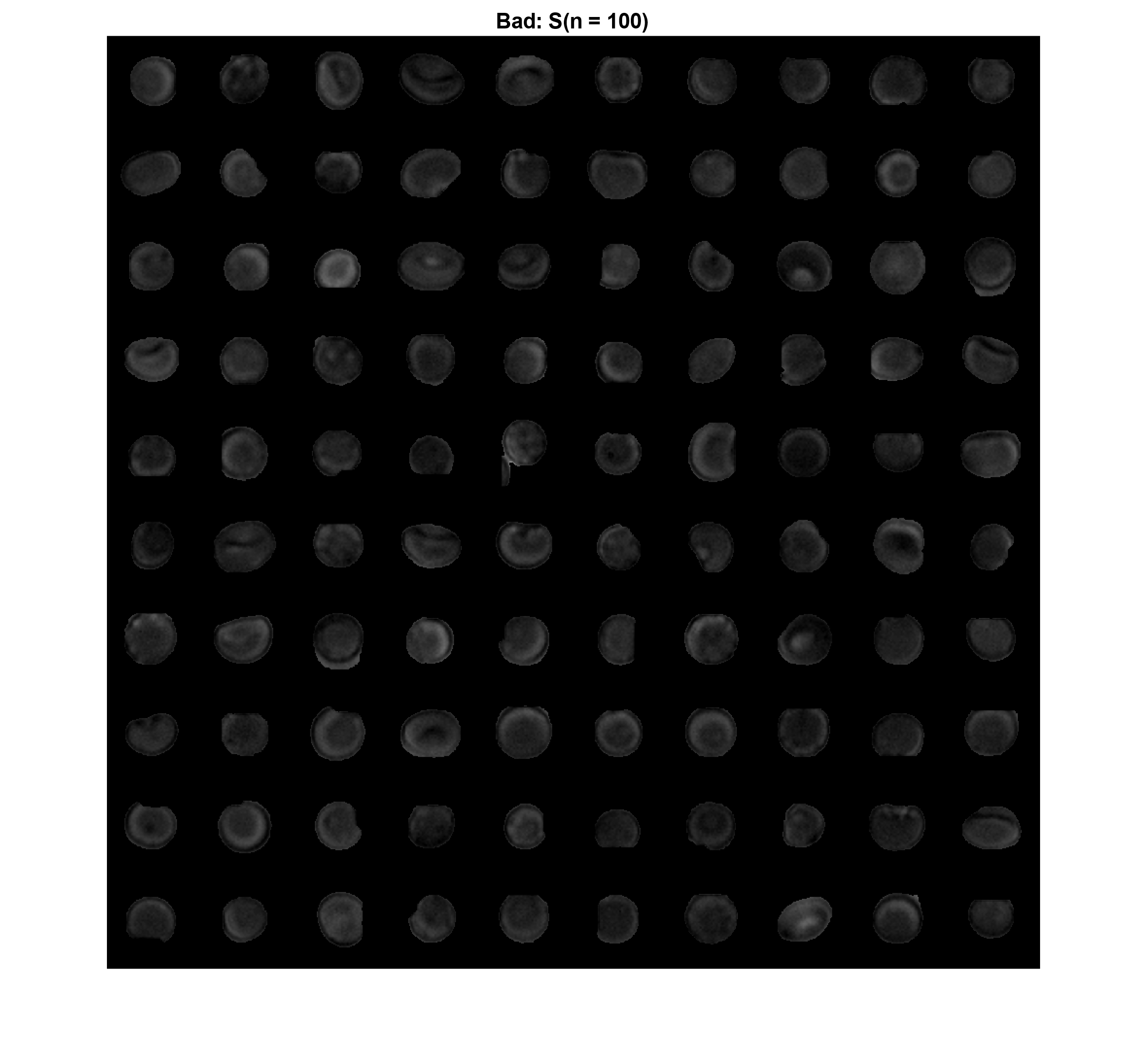 |
| G)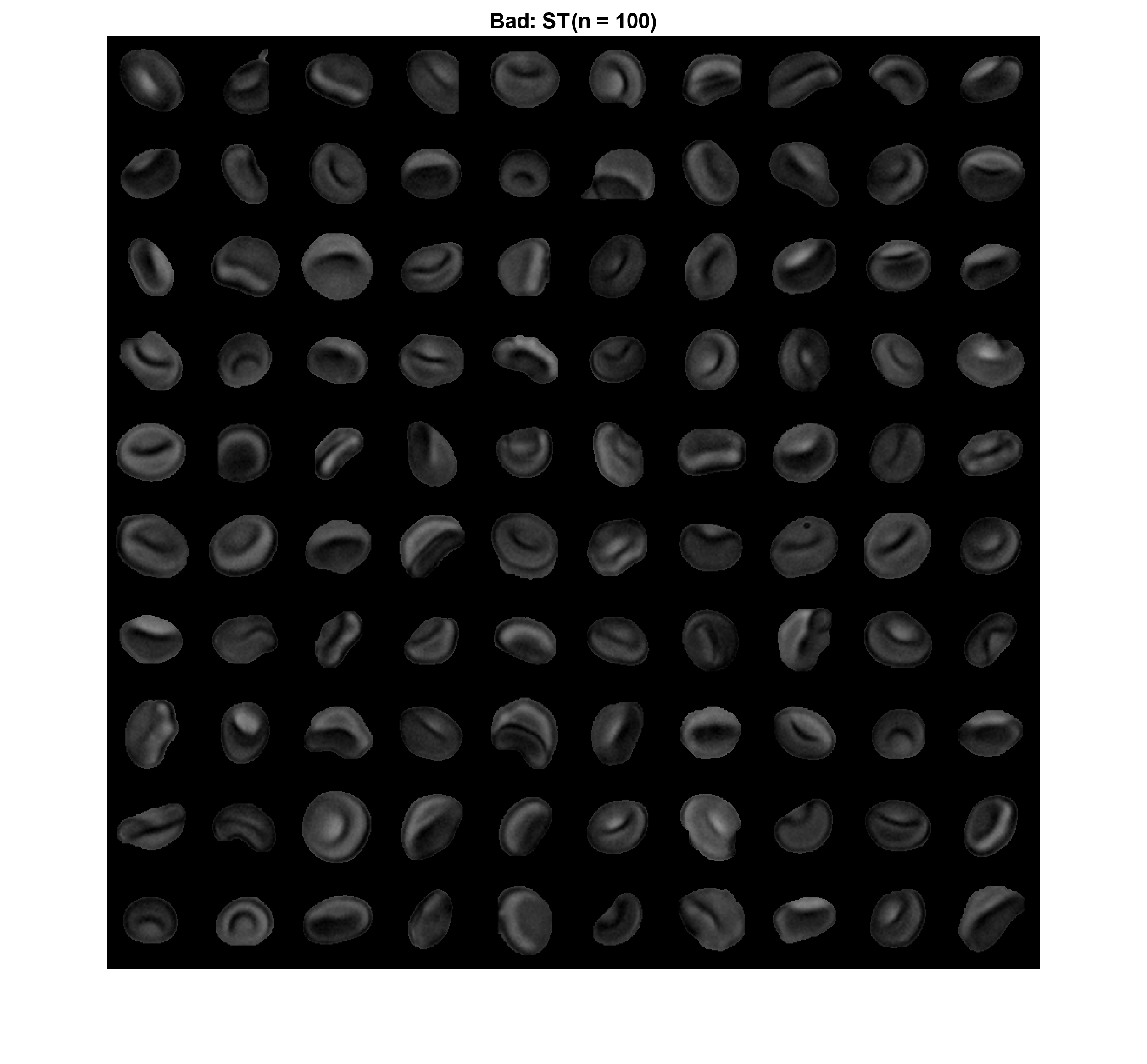 |  | |

**Figure S9:** A hundred randomly sampled bad (A) discocytes, (B) stage 1 echinocytes, (C) stage 2 echinocytes, (D) stage 3 echinocytes, (E) sphero-echinocytes, (F) spherocytes, and (G) stomatocytes.

**Figure S10A**


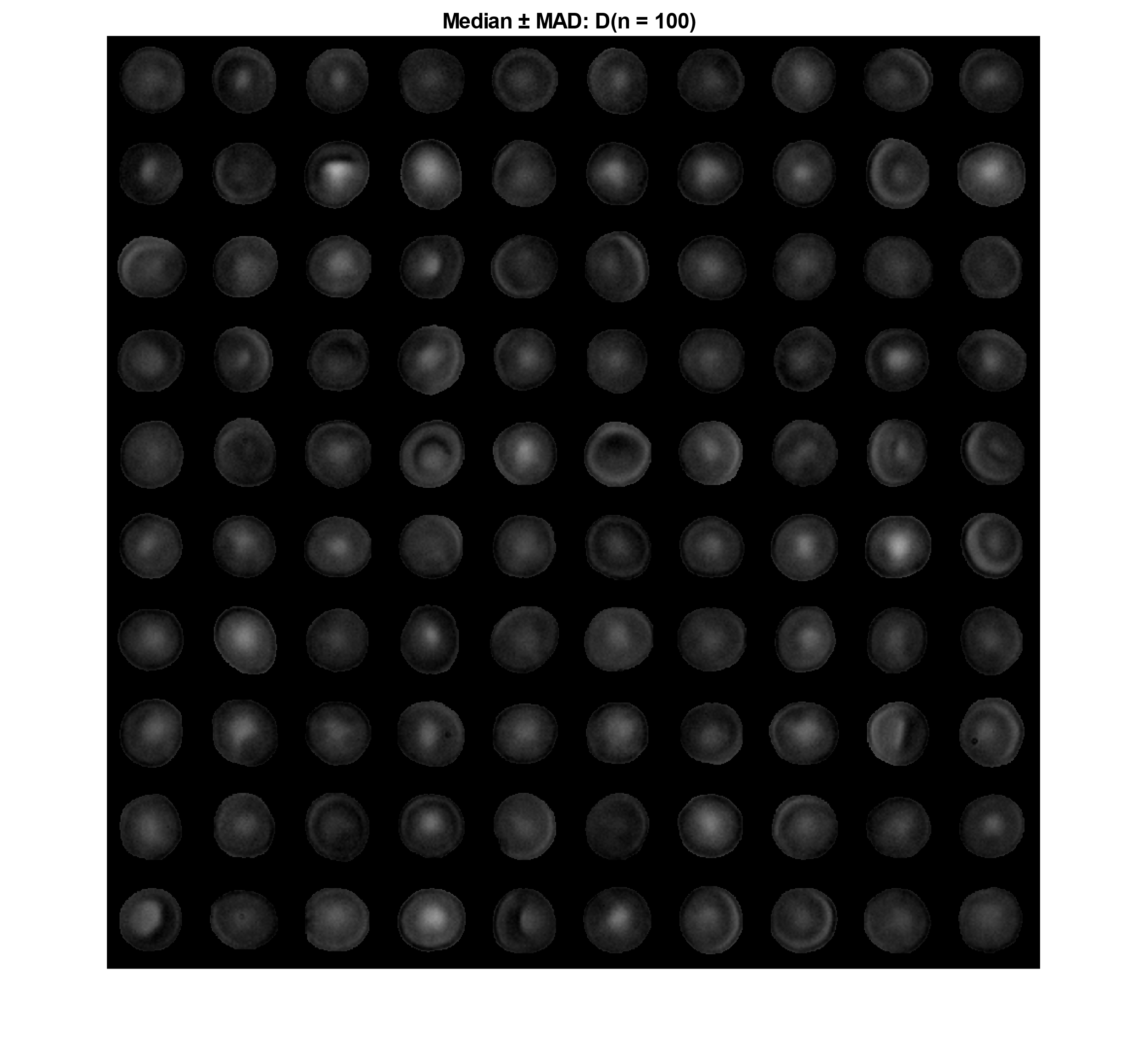


**Figure S10A:** A random selection of 100 discocytes within one median absolute deviation of the median effective diameter of the subset of good discocytes.

**Figure S10B**


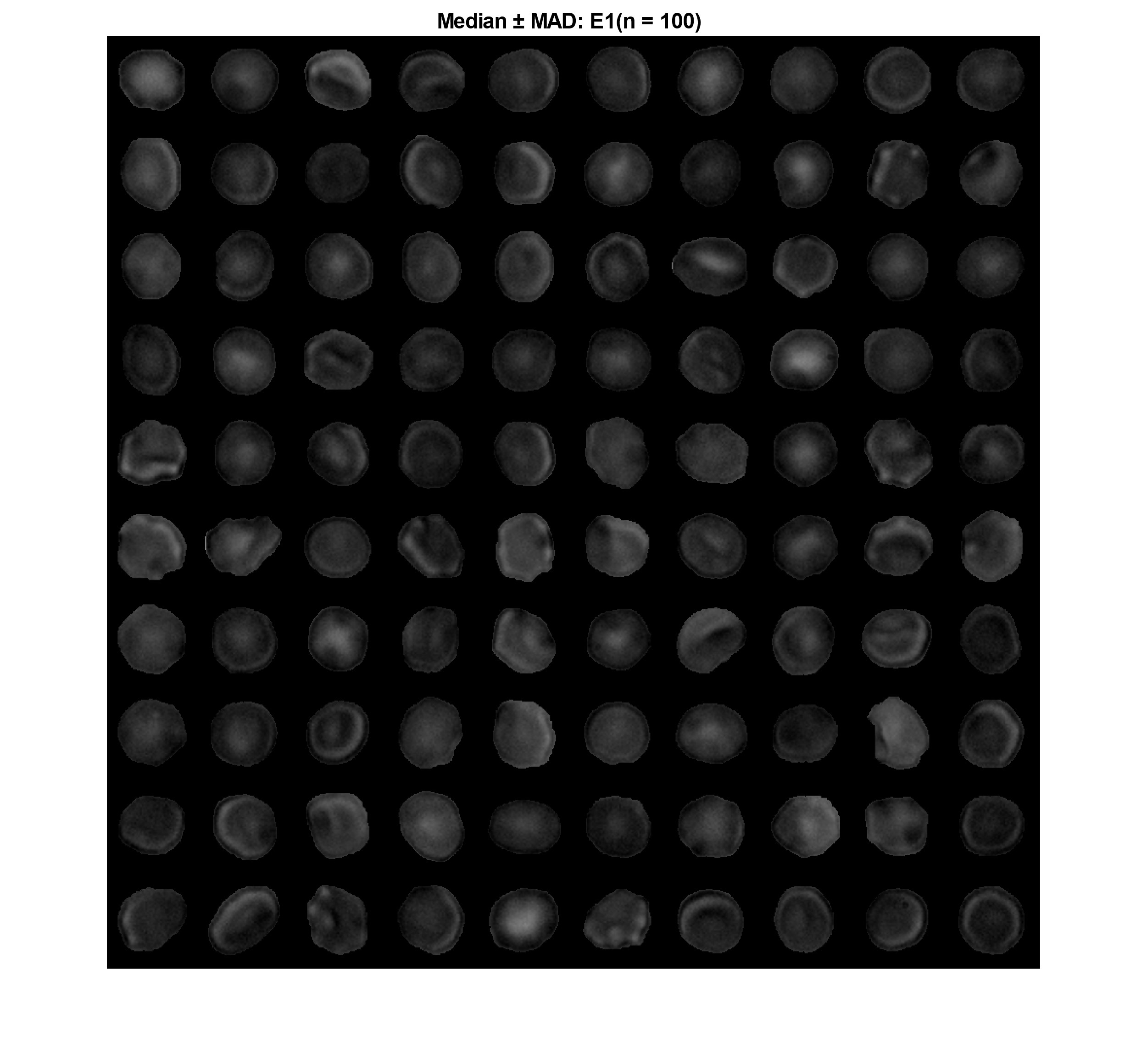


**Figures S10B:** A random selection of 100 echinocyte 1 RBCs within one median absolute deviation of the median effective diameter of the subset of good stage 1 echinocytes.

**Figure S10C**


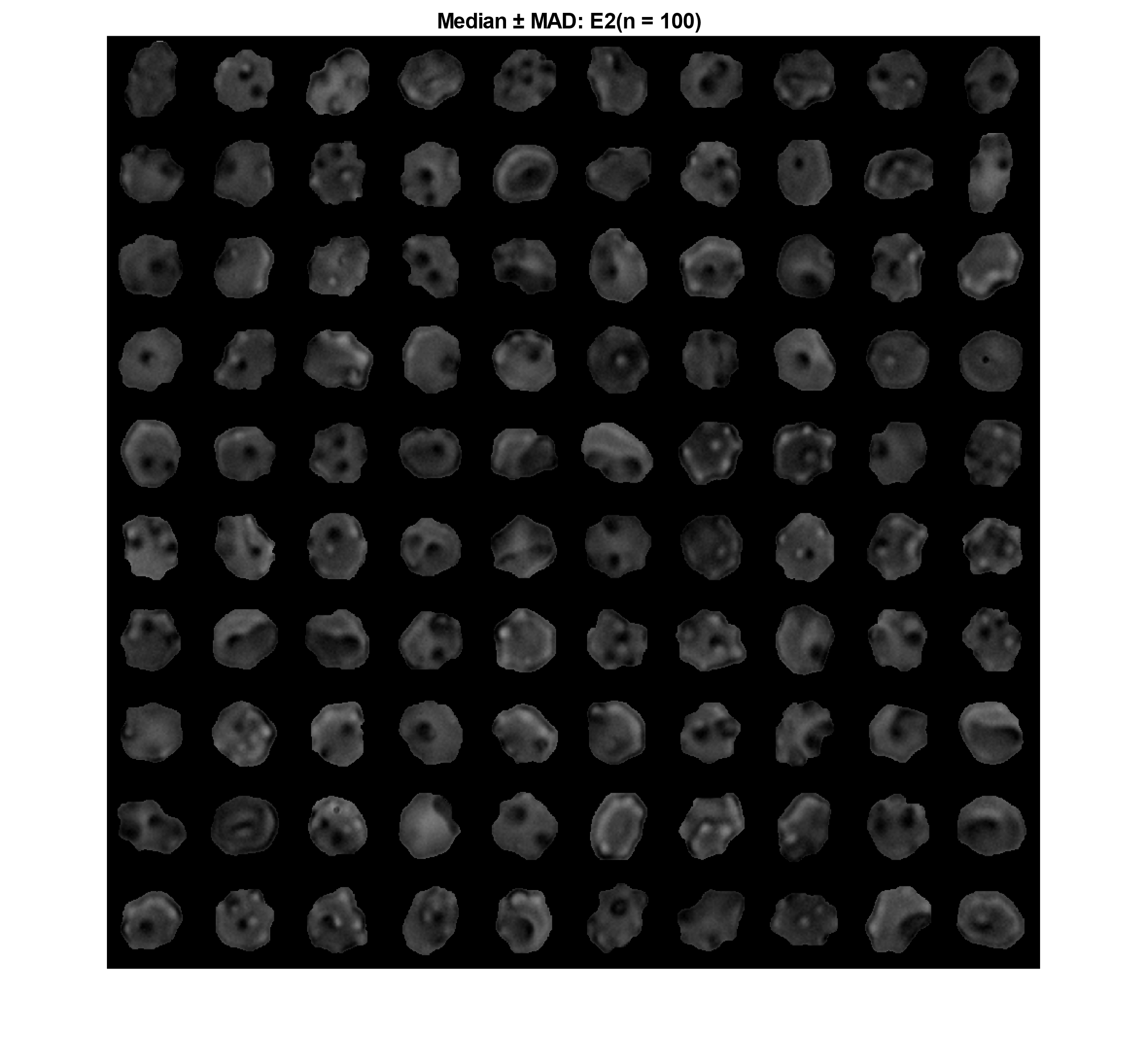


**Figures S10C:** In the subset of good echinocyte stage 2 RBCs, 100 RBCs are selected at random within one median absolute deviation of the median effective diameter.

**Figure S10D**


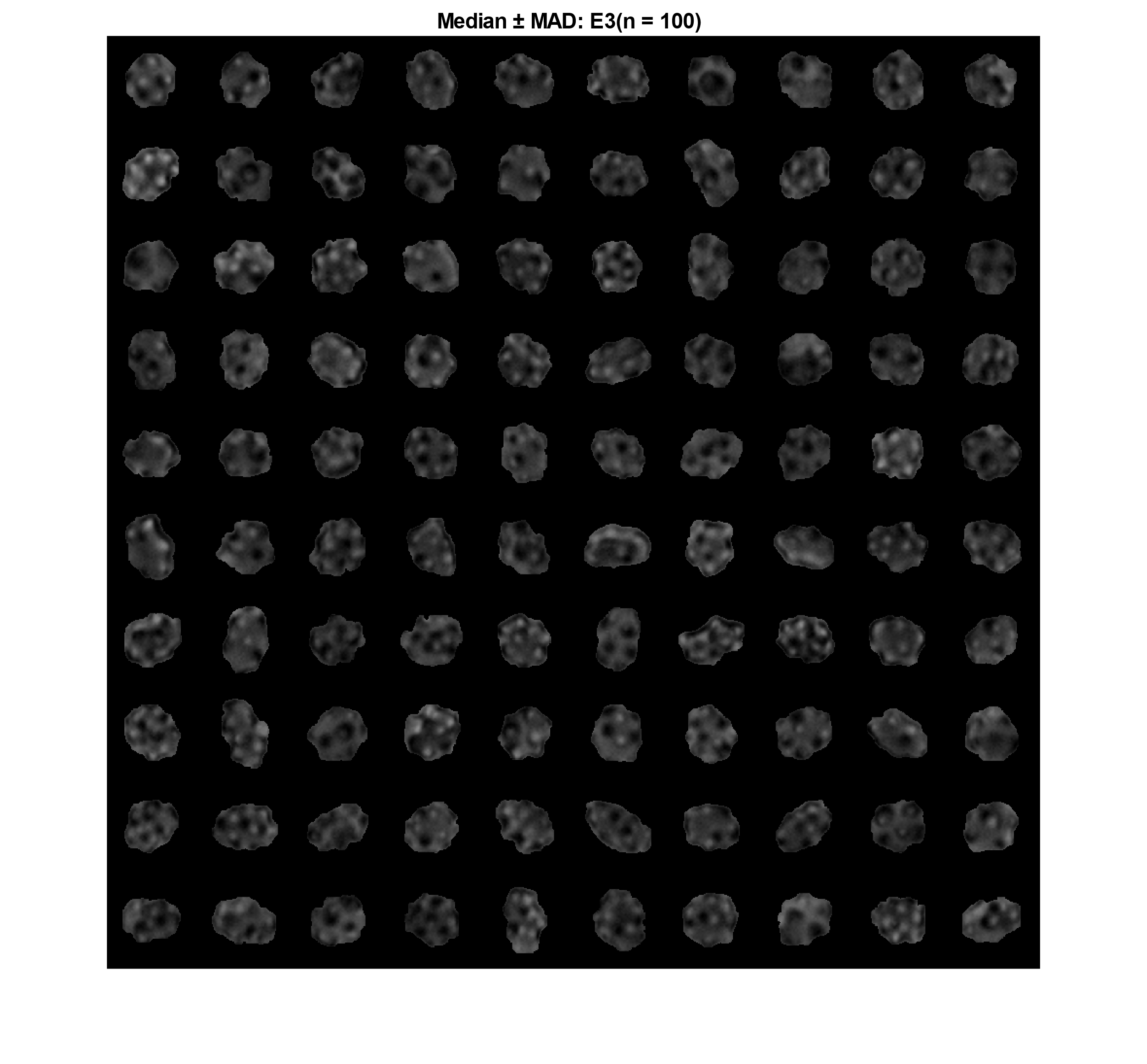
**Figures S10D:** A random selection of 100 echinocyte 3 RBCs within one median absolute deviation of the median effective diameter of the subset of good stage 3 echinocytes.

**Figure S10E**


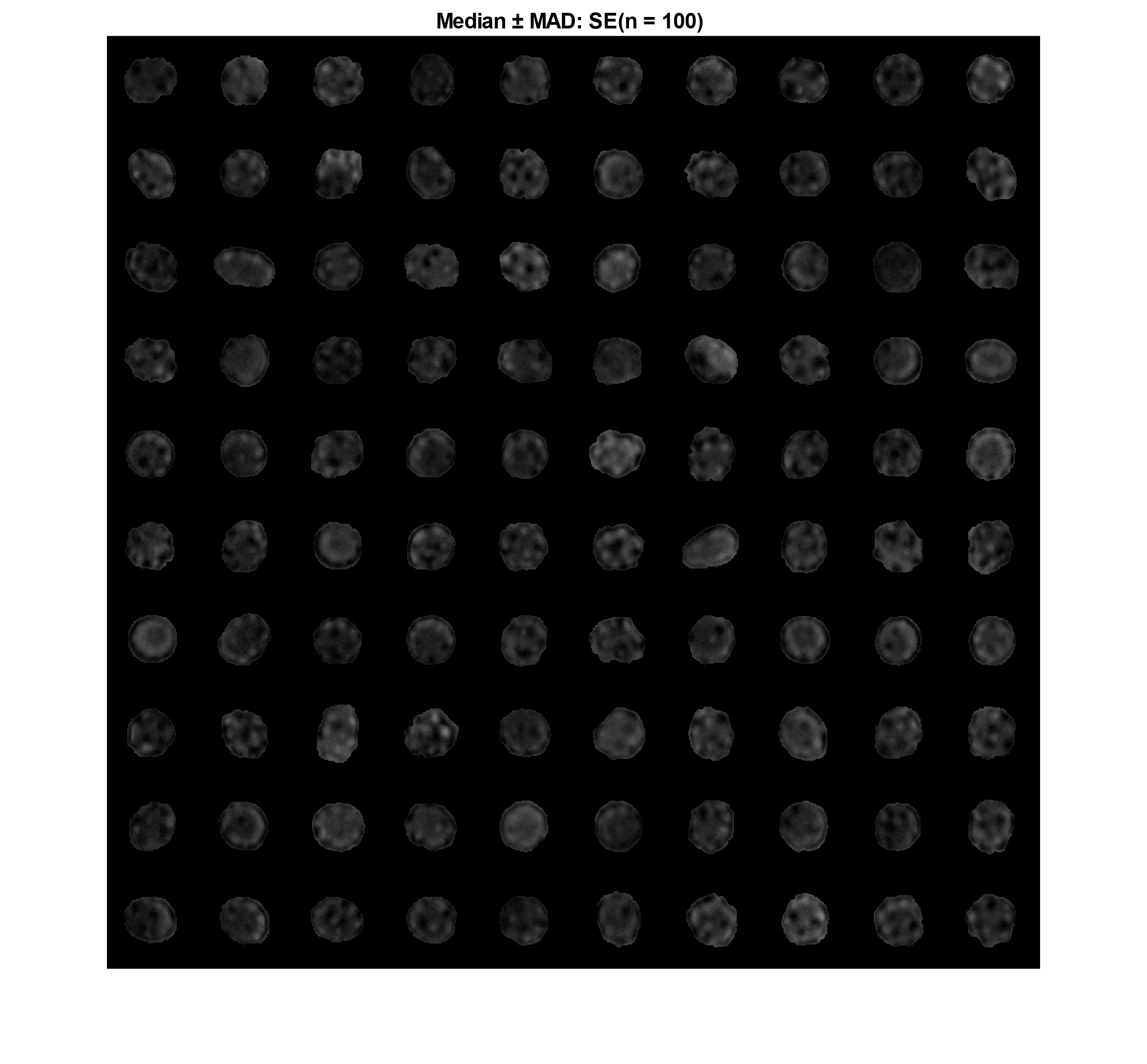


**Figures S10E:** 100 spheroechinocytes selected randomly from within one median absolute deviation of the median effective diameter of the good subset.

**Figure S10F**


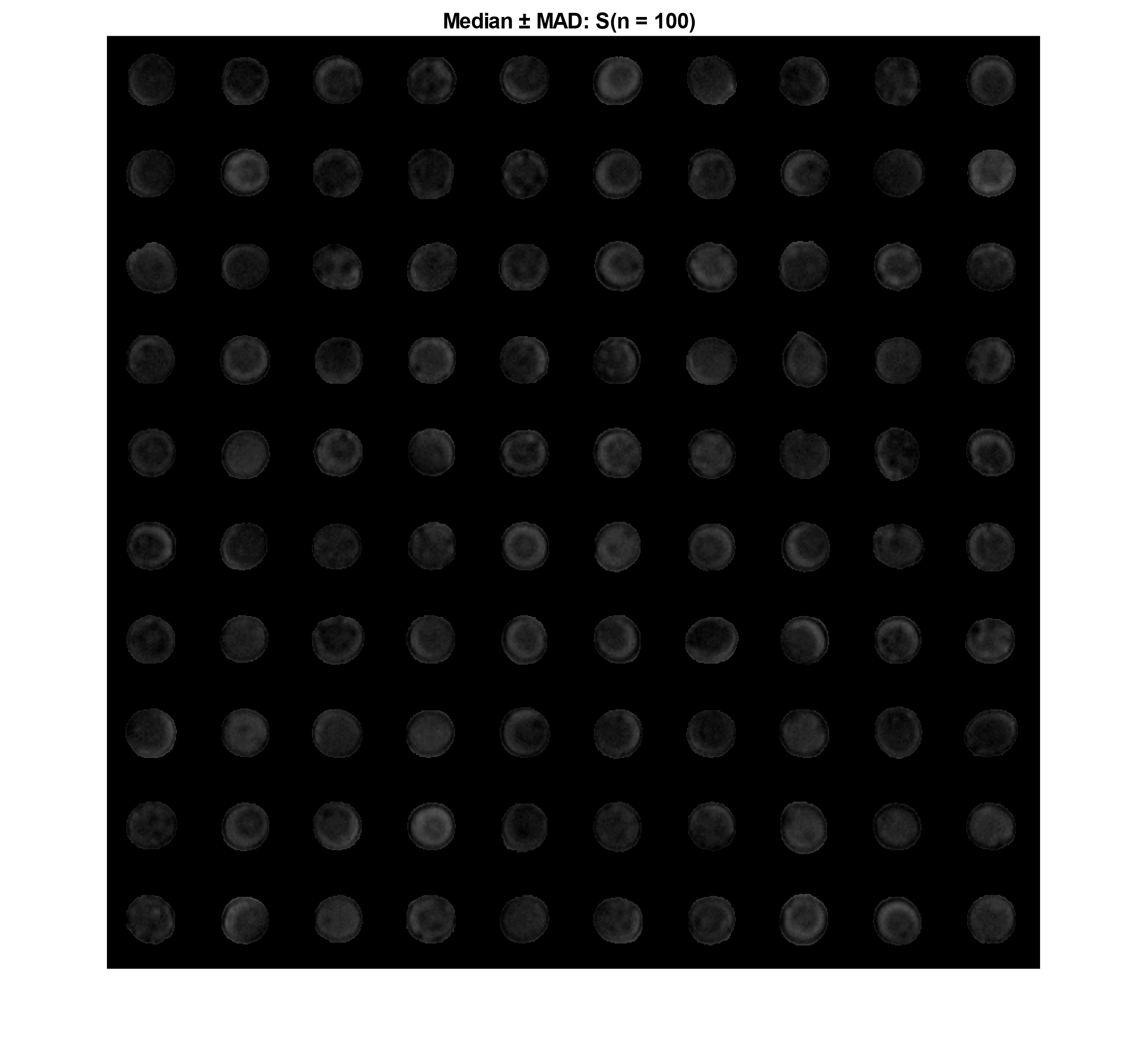


**Figures S10F:** 100 spherocytes were selected randomly from within one median absolute deviation of the median effective diameter of the good subset.

**Figure S10G**


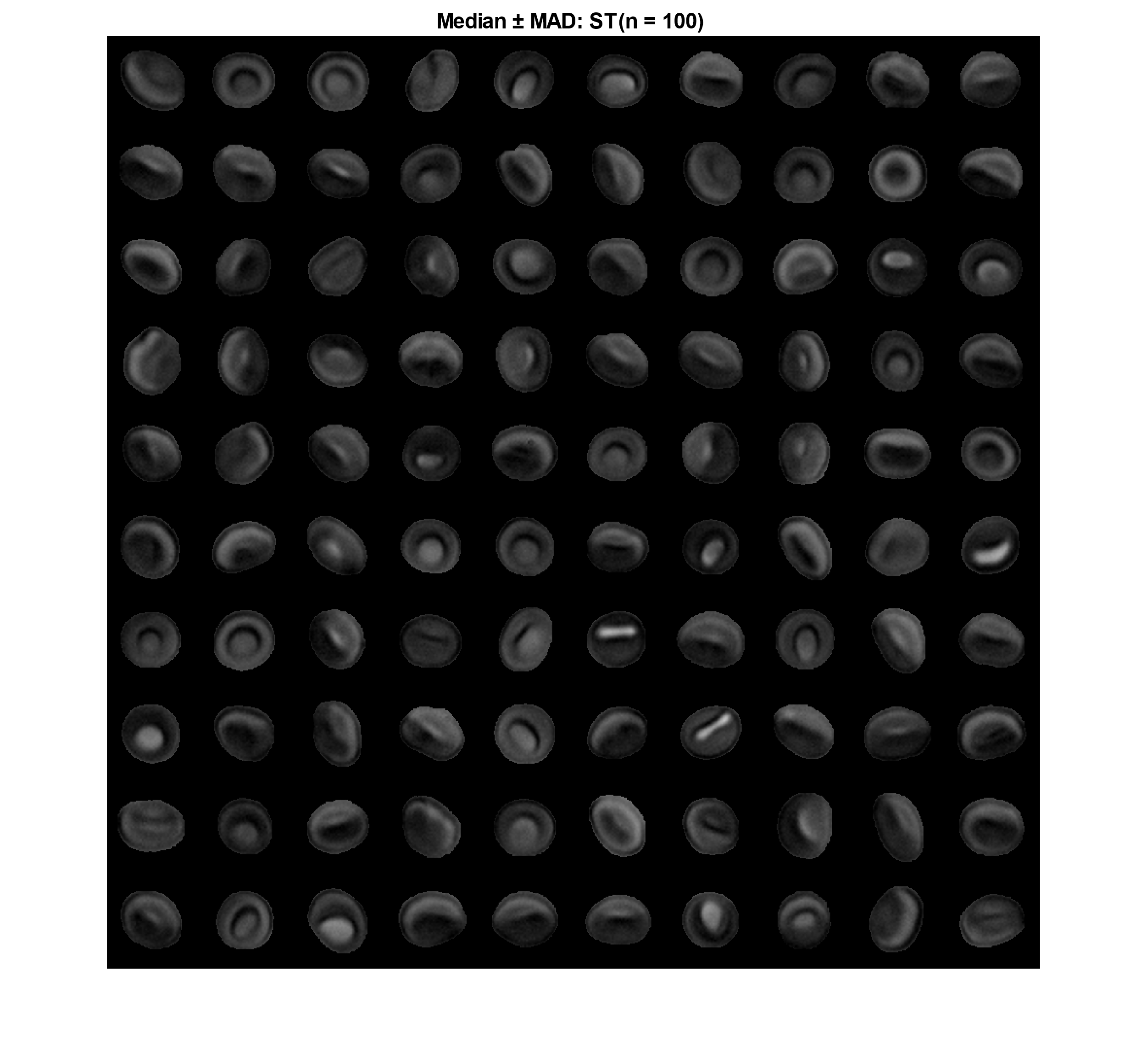


**Figures S10G:** A random selection of 100 stomatocytes within one median absolute deviation of the median effective diameter of the subset of good stomatocytes.

**Figure S11**

| A)  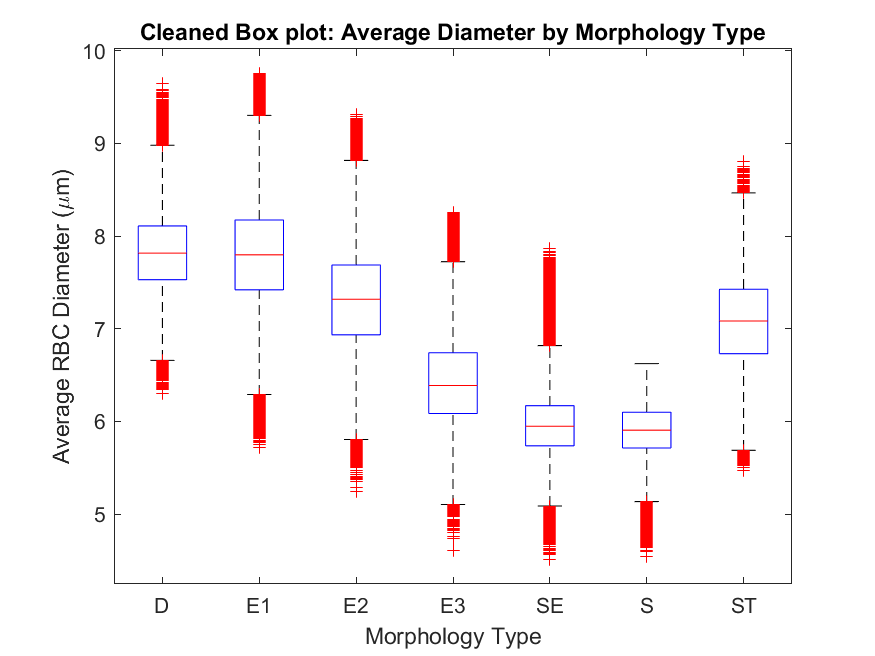 | B)  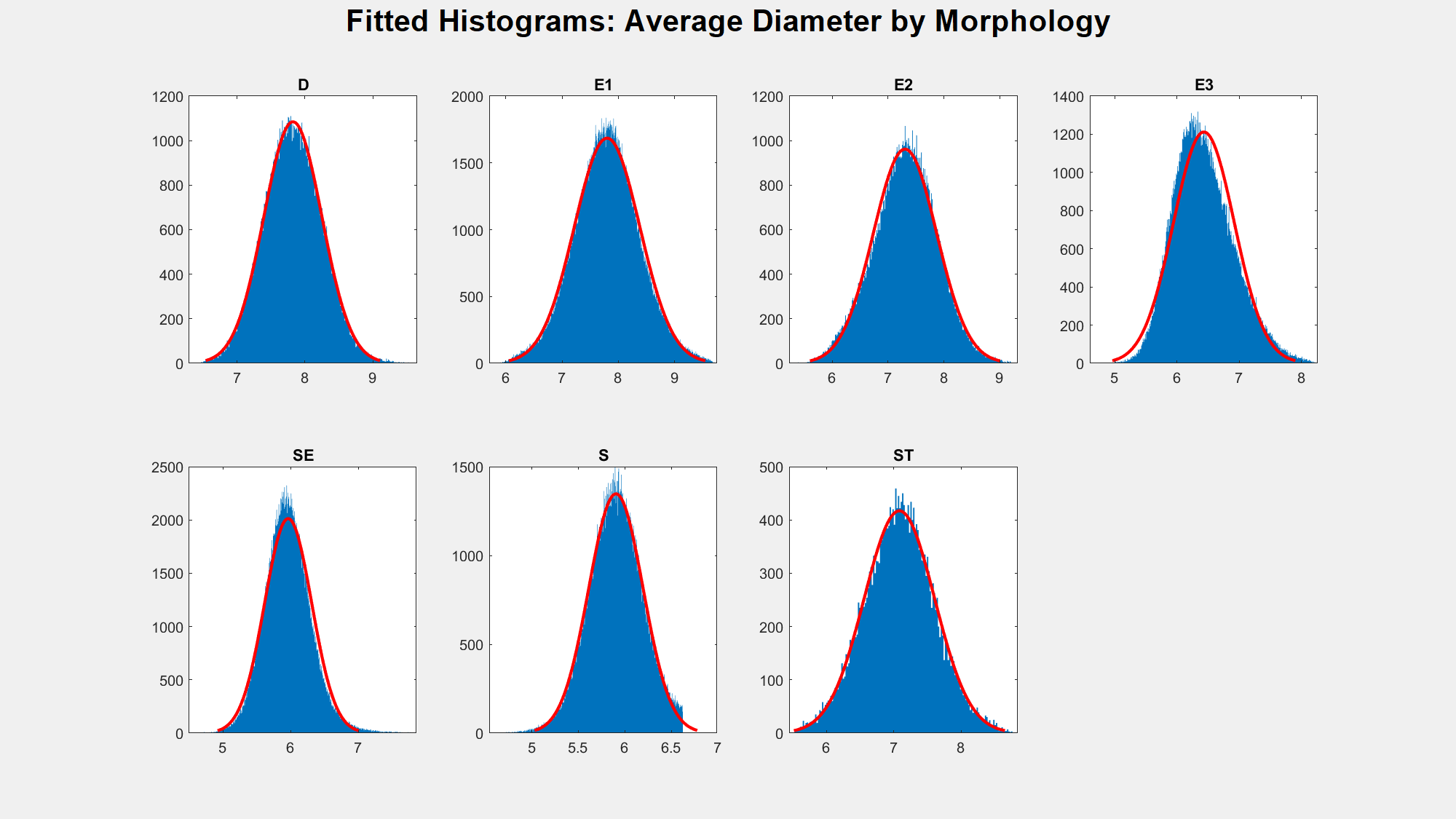 |
| --- | --- |
| C)  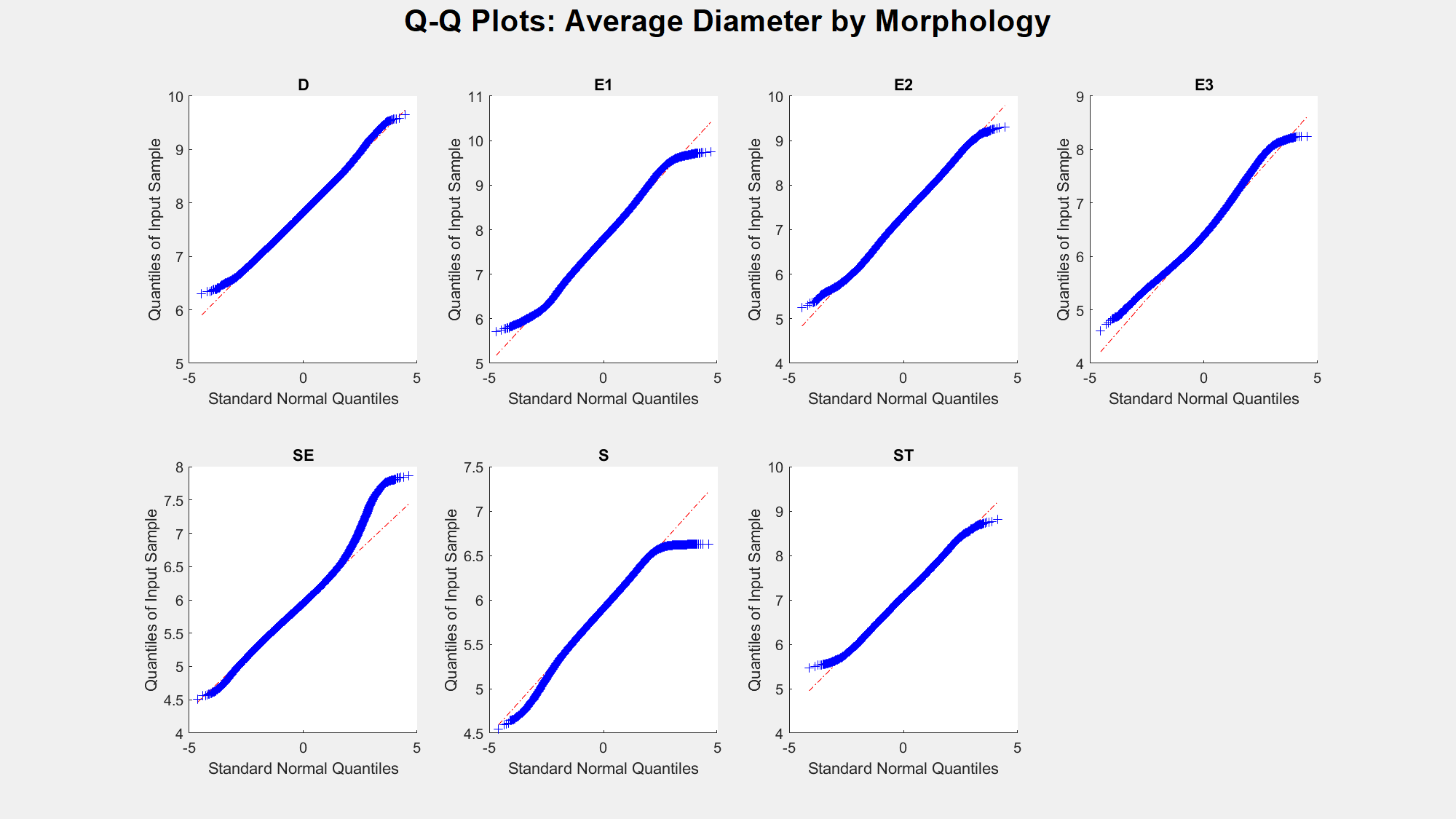 | D)  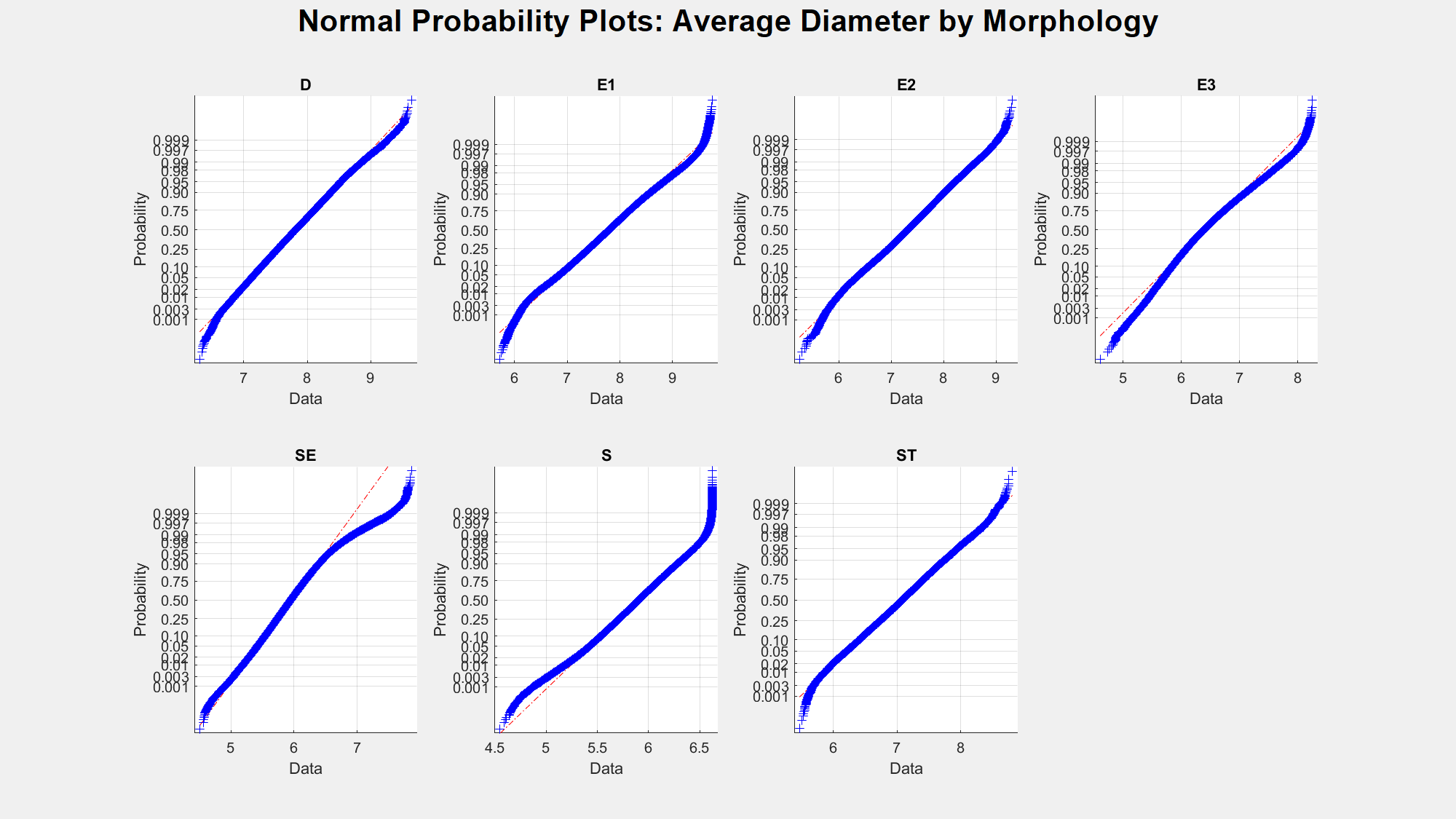 |

**Figures S11:** A) (top-left) The boxplot diagram of the cleaned effective high-resolution diameter indicating a significant decrease in statistical outliers. B) (top-right) Fitted histograms of each morphology’s effective diameter distribution, showing approximately normal distributions. C) (bottom-left) Q-Q plots of each morphology’s effective diameter distribution show much less deviation from normality than the raw data. D) (bottom-right) Normal probability plots of each morphology’s effective diameter distribution, also indicating approximately normal distributions.

**Table S1:** A table showing the descriptive statistics of the cleaned subset from the MH dataset & fresh discocyte data.

| **Morphology** | **Means [μm]** | **SD  [μm]** | **Mean MAD [μm]** | **Medians [μm]** | **Median MAD [μm]** | **IQR [μm]** | **Range [μm]** | **Skewness** | **Kurtosis** | **Sample Size** |
| --- | --- | --- | --- | --- | --- | --- | --- | --- | --- | --- |
| Fresh D | 7.6465 | 0.4531 | 0.3609 | 7.6510 | 0.3028 | 0.6056 | 3.3030 | -0.0487 | 2.9733 | 37273 |
| D | 7.8214 | 0.4288 | 0.3424 | 7.8160 | 0.2901 | 0.5803 | 3.3422 | 0.0922 | 3.0483 | 121571 |
| E1 | 7.7997 | 0.5815 | 0.4578 | 7.7976 | 0.3766 | 0.7531 | 4.0377 | 0.0382 | 3.1525 | 368292 |
| E2 | 7.3041 | 0.5675 | 0.4512 | 7.3177 | 0.3759 | 0.7530 | 4.0636 | -0.0705 | 2.9642 | 112885 |
| E3 | 6.4328 | 0.4897 | 0.3902 | 6.3869 | 0.3242 | 0.6553 | 3.6408 | 0.4398 | 3.1203 | 167345 |
| SE | 5.9625 | 0.3478 | 0.2679 | 5.9479 | 0.2165 | 0.4328 | 3.3538 | 0.4386 | 4.2611 | 273332 |
| S | 5.9039 | 0.2917 | 0.2312 | 5.9052 | 0.1926 | 0.3855 | 2.0772 | -0.1360 | 3.1136 | 224930 |
| ST | 7.0804 | 0.5223 | 0.4150 | 7.0835 | 0.3477 | 0.6957 | 3.3389 | 0.0156 | 2.9525 | 26641 |

# **Morphological Heterogeneity Dataset: Effective Diameter Hypothesis Testing & Effect Size**

Using the cleaned effective diameter distributions, we test for a difference in means and medians using the two-sample t-test for unequal variances, the Kruskal–Wallis test, and bootstrap hypothesis tests. Bootstrap tests used 10,000 replicates (**Table S2**). For all 21 comparisons, we set the significance level to 0.05 and used a Bonferroni adjustment for a conservative adjusted alpha of approximately 0.00238. All tests resulted in p-values below the adjusted significance level. Therefore, we reject the null hypothesis that there is no difference between the morphology effective diameter means or medians.

After hypothesis testing, we use a ratio of the difference in means and average standard deviation to calculate Cohens d for an analysis of effect size:

$$d= \frac{M_{2}-M_{1}}{\sqrt{\frac{{SD}_{1}+{SD}_{2}}{2}}}$$

All non-neighboring morphology effective diameter means had effect sizes there were relatively large (>1), but for D & E1, E2 & ST, and SE & S the effect sizes were small (<0.5). In other words, the number of standard deviations that lie between the non-neighboring effective diameter means ranged from about 1.03 (D & E2) to 5.22 (D & S). However, the number of standard deviations between neighboring effective diameter means ranged from 0.04 (D&E1) to 0.86 (E1 & E2).

**Table S2:** A table showing the p-values from hypothesis testing and values relating to Cohen’s d.

| **Group 1** | **Group 2** | **p-value (t-test)** | **p-value (Kruskal–Wallis)** | **p-value (Bootstrap t-test)** | **p-value (Difference in Median Bootstrap Test)** | **Mean Difference (Group 1- Group 2)** | **Average Standard Deviation** | **Cohen's d** |
| --- | --- | --- | --- | --- | --- | --- | --- | --- |
| D | E1 | 3.66008E-44 | 0 | 0 | 9.999E-05 | 0.021734559 | 0.510864767 | 0.042544644 |
| D | E2 | 0 | 0 | 0 | 9.999E-05 | 0.517271356 | 0.502937622 | 1.028500022 |
| D | E3 | 0 | 0 | 0 | 9.999E-05 | 1.38858099 | 0.460231143 | 3.017138261 |
| D | SE | 0 | 0 | 0 | 9.999E-05 | 1.858872743 | 0.39040822 | 4.761356566 |
| D | S | 0 | 0 | 0 | 9.999E-05 | 1.917488145 | 0.36669913 | 5.229050157 |
| D | ST | 0 | 0 | 0 | 9.999E-05 | 0.740951967 | 0.477821218 | 1.550688708 |
| E1 | E2 | 0 | 0 | 0 | 9.999E-05 | 0.495536796 | 0.574520049 | 0.86252307 |
| E1 | E3 | 0 | 0 | 0 | 9.999E-05 | 1.36684643 | 0.537531152 | 2.542822728 |
| E1 | SE | 0 | 0 | 0 | 9.999E-05 | 1.837138183 | 0.479109187 | 3.834487488 |
| E1 | S | 0 | 0 | 0 | 9.999E-05 | 1.895753585 | 0.459994877 | 4.121249345 |
| E1 | ST | 0 | 0 | 0 | 9.999E-05 | 0.719217408 | 0.552666401 | 1.301359023 |
| E2 | E3 | 0 | 0 | 0 | 9.999E-05 | 0.871309634 | 0.530003001 | 1.643971132 |
| E2 | SE | 0 | 0 | 0 | 9.999E-05 | 1.341601387 | 0.470647485 | 2.850544048 |
| E2 | S | 0 | 0 | 0 | 9.999E-05 | 1.400216789 | 0.451174832 | 3.103490465 |
| E2 | ST | 0 | 0 | 0 | 9.999E-05 | 0.223680611 | 0.545347222 | 0.410161824 |
| E3 | SE | 0 | 0 | 0 | 9.999E-05 | 0.470291753 | 0.424706379 | 1.107333859 |
| E3 | S | 0 | 0 | 0 | 9.999E-05 | 0.528907155 | 0.403020076 | 1.312359325 |
| E3 | ST | 0 | 0 | 0 | 9.999E-05 | -0.647629023 | 0.506231218 | -1.279314669 |
| SE | S | 0 | 0 | 0 | 9.999E-05 | 0.058615402 | 0.32098451 | 0.18261131 |
| SE | ST | 0 | 0 | 0 | 9.999E-05 | -1.117920776 | 0.44370702 | -2.519502118 |
| S | ST | 0 | 0 | 0 | 9.999E-05 | -1.176536178 | 0.422995973 | -2.781435884 |

# **Morphological Heterogeneity Dataset: Visualizing Effective Diameter Bootstrap Statistics**

**Figure S12**

| A)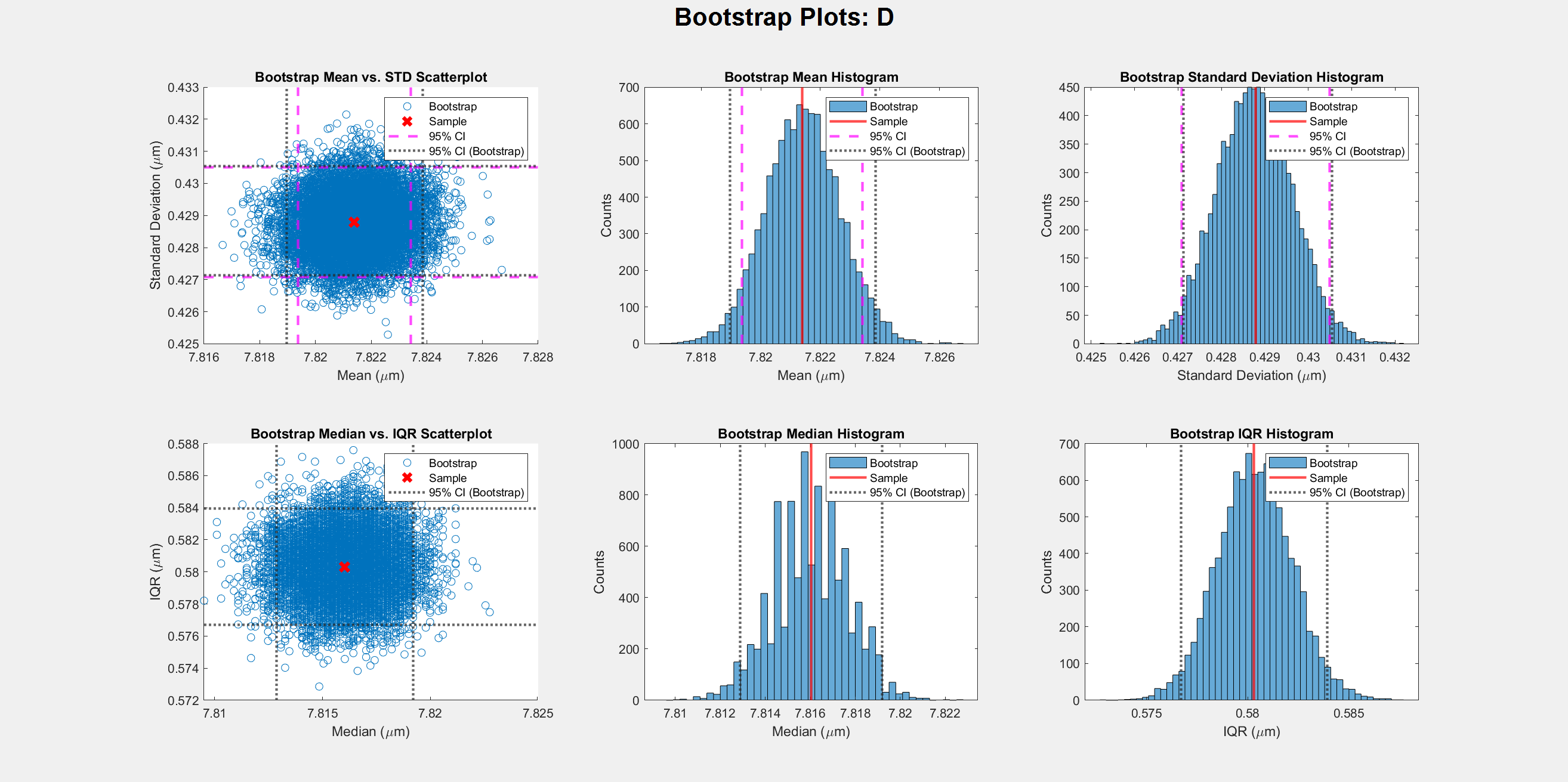 | B)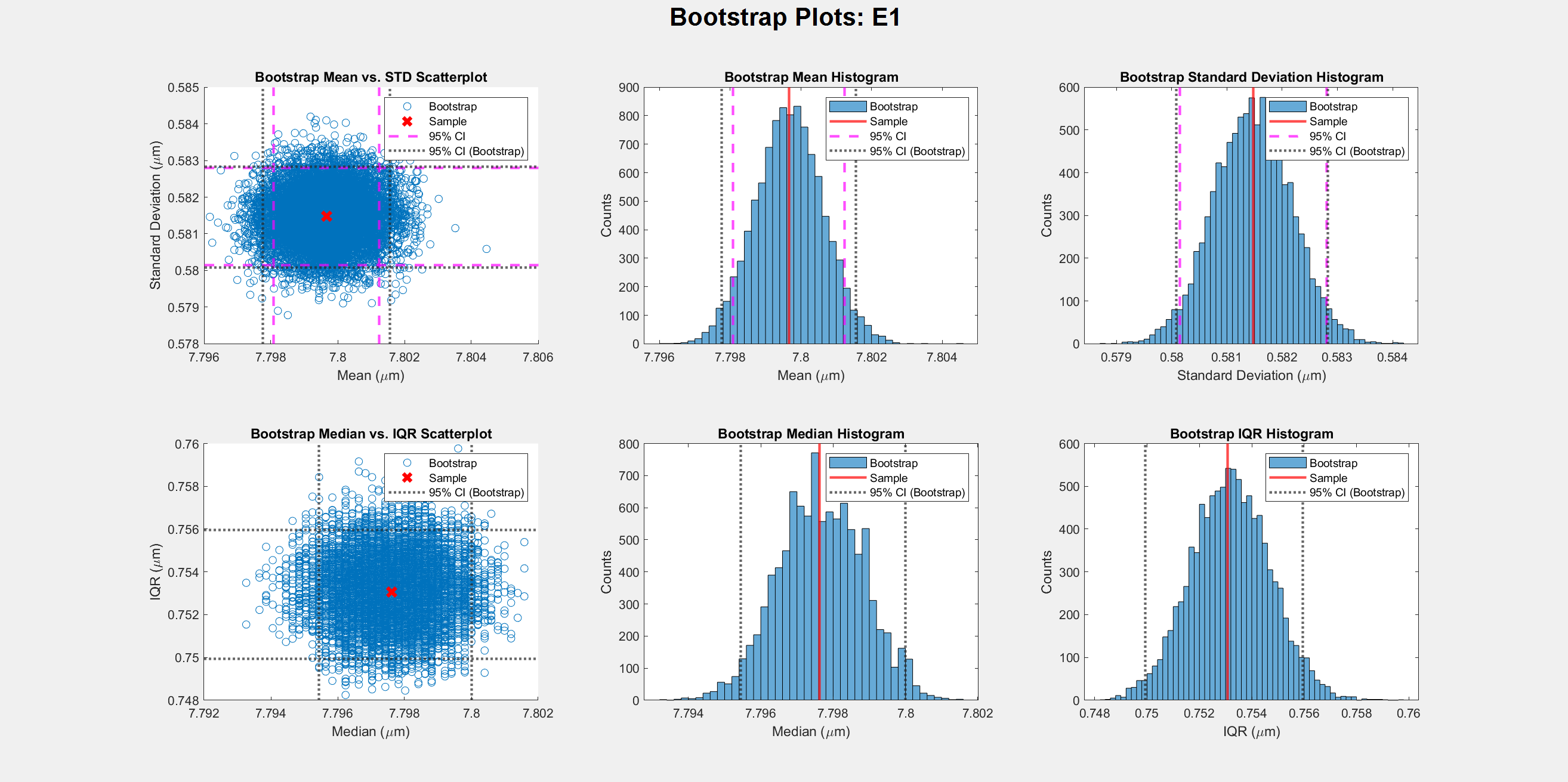 |
| --- | --- |
| C)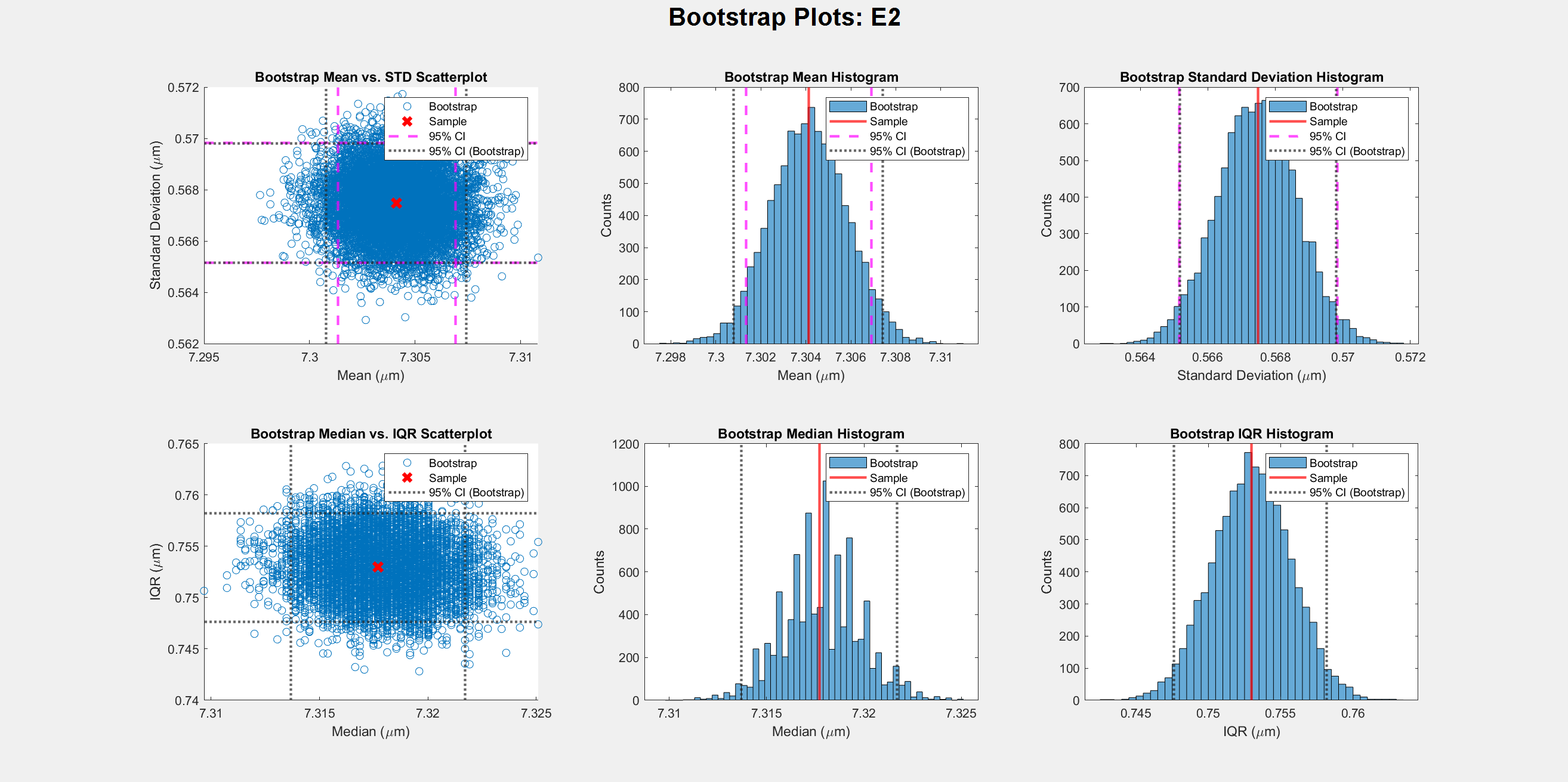 | D)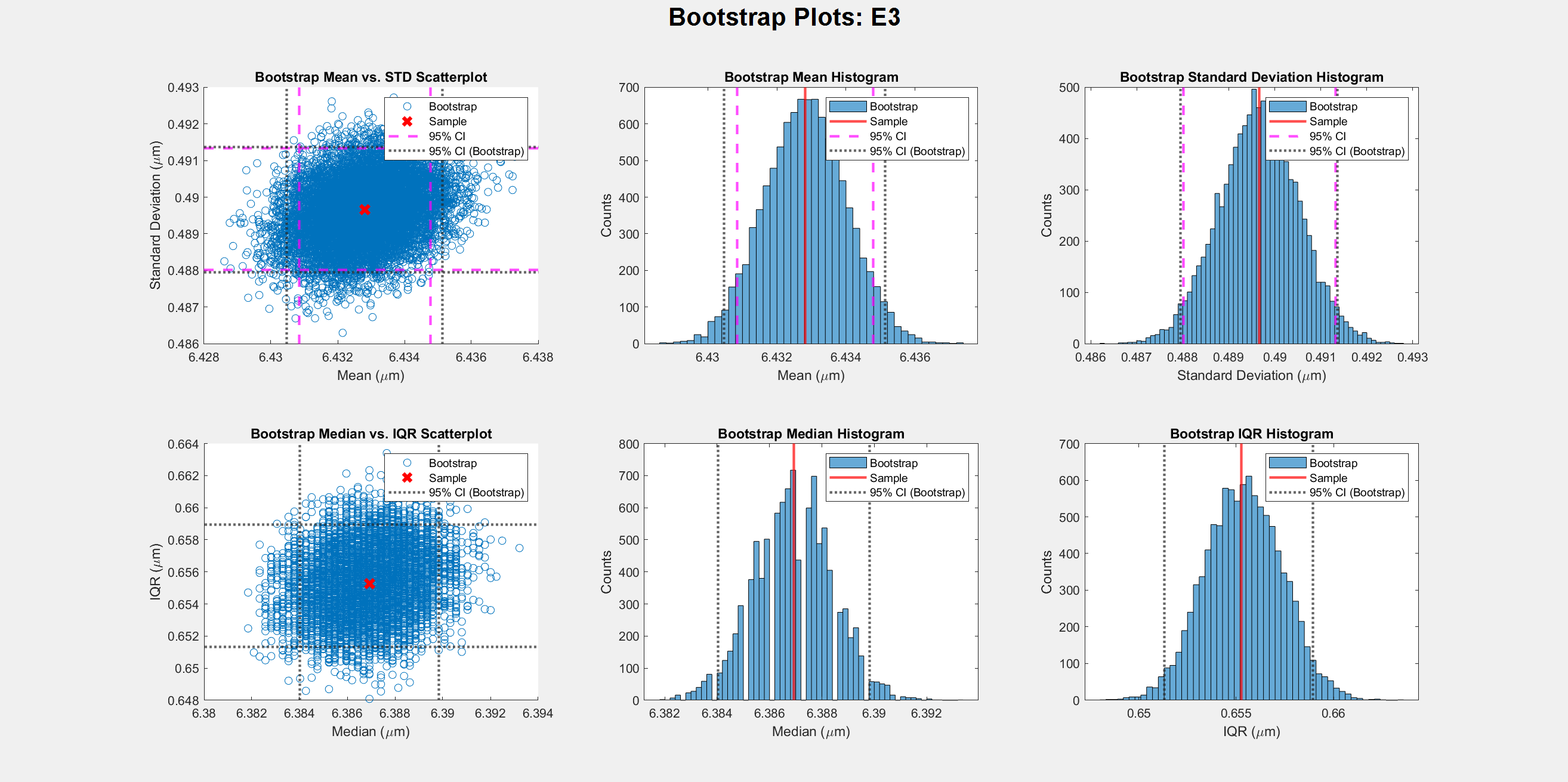 |
| E)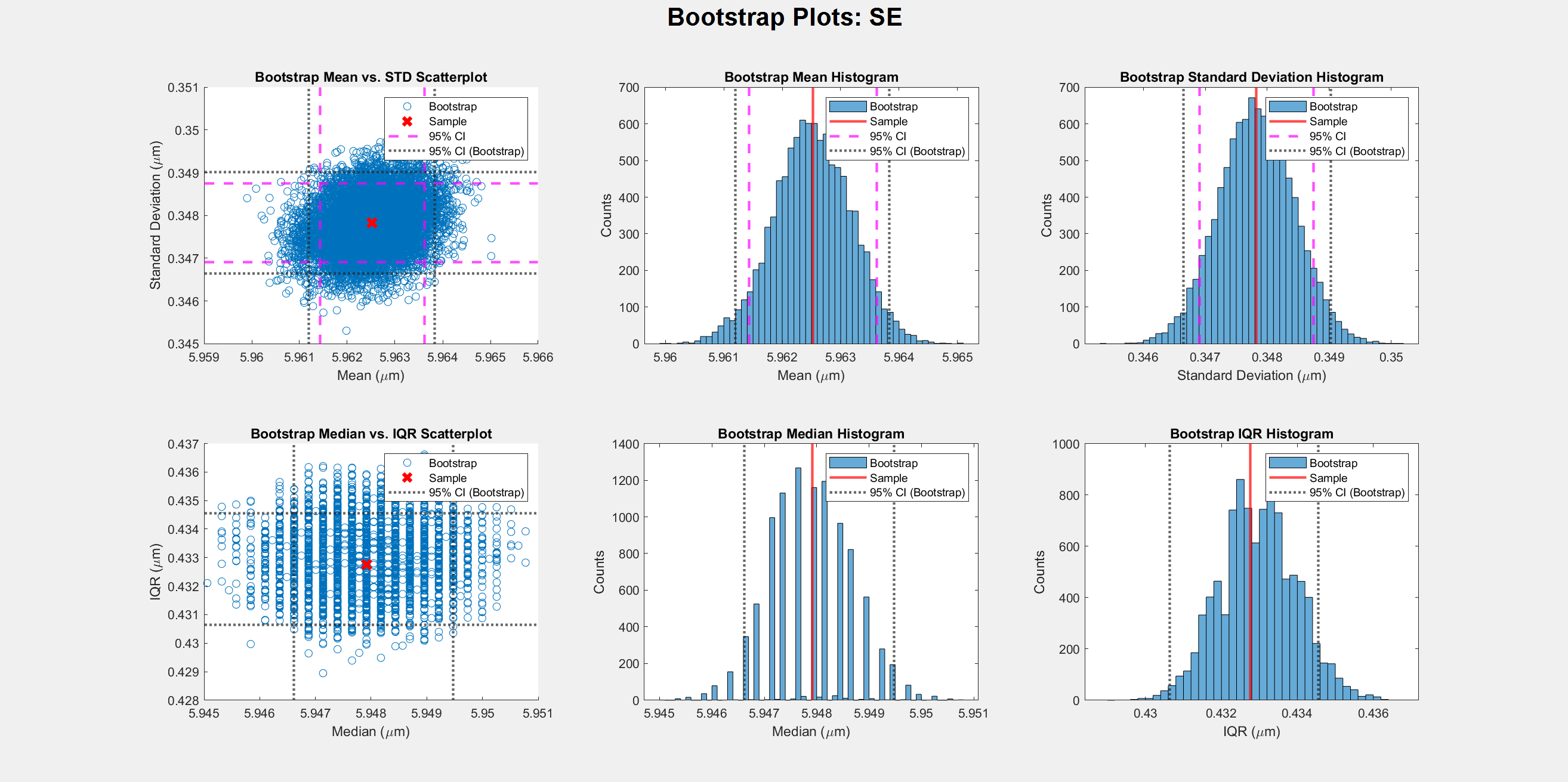 | F)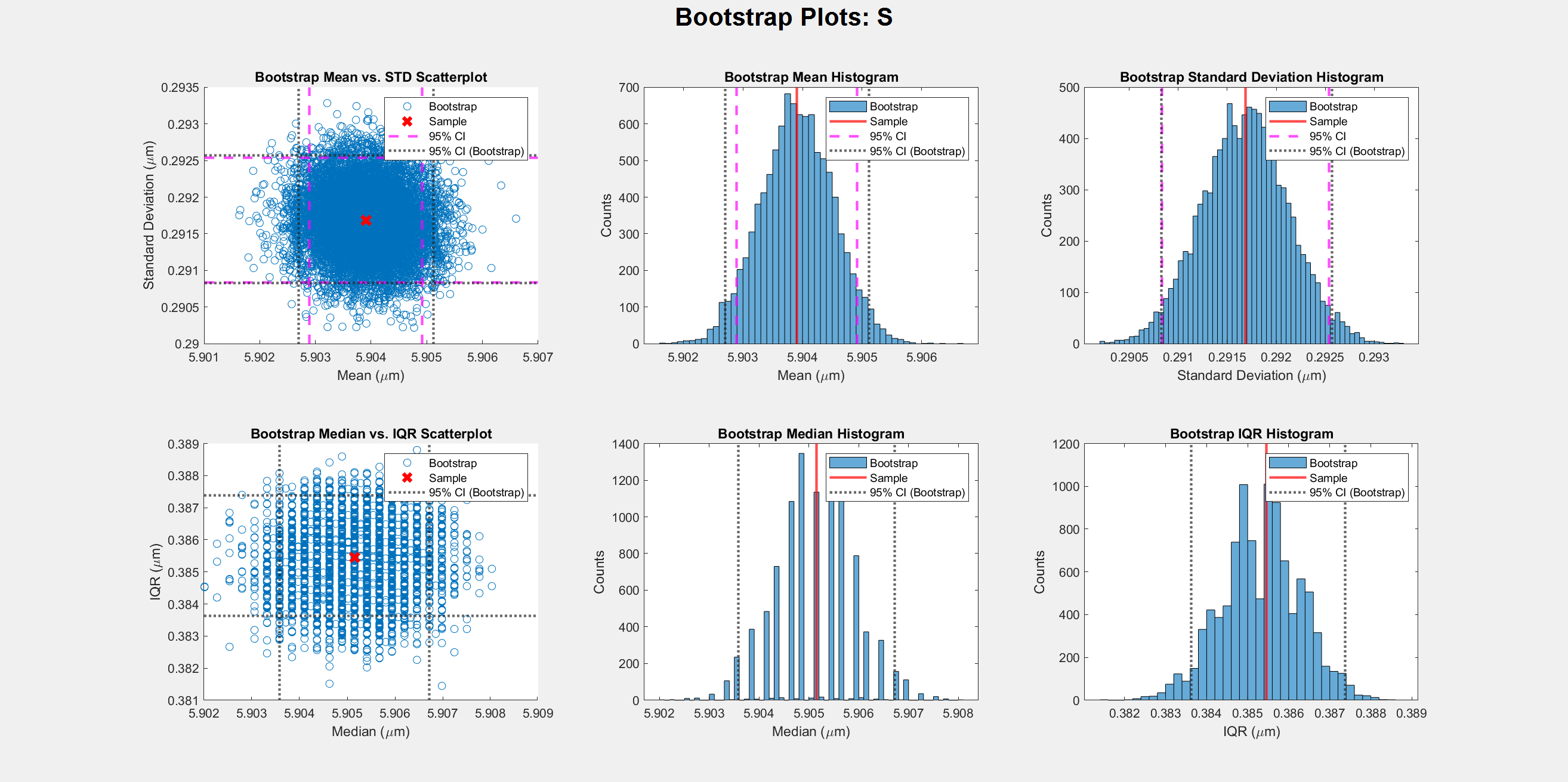 |
| G)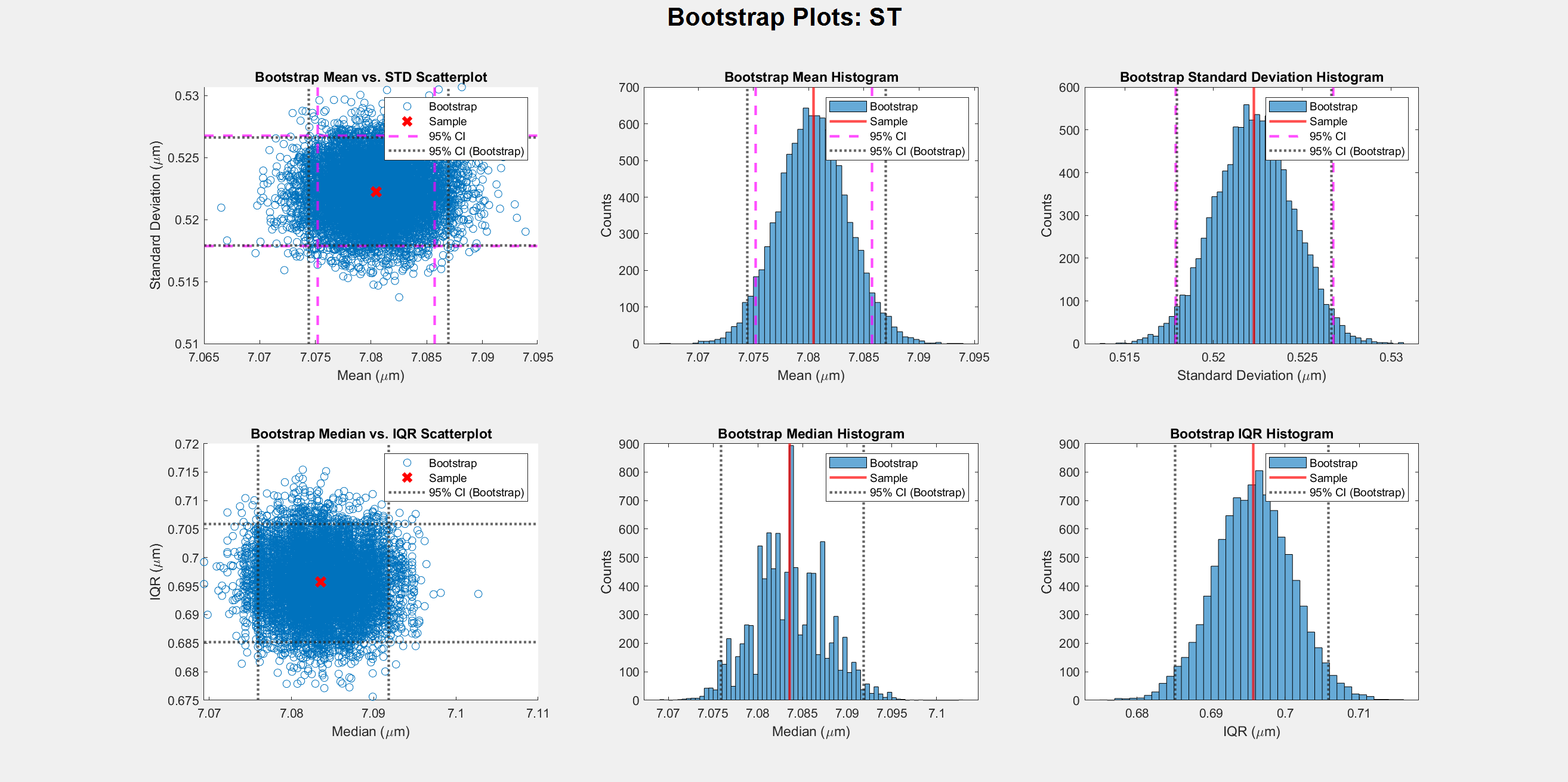 | |

**Figure S12:** For each morphology type, a visualization of 10,000 bias-corrected bootstrap replicates for mean, standard deviation, median, and interquartile range is shown.

# **Singular CNN & Ensemble Analysis**

We made graphs to show our model's individual CNN and cumulative ensemble accuracy when tested against the MH, CIW, and combined test sets. **Figure S13A-B** depict model accuracy after training on the MH training set, whereas **Figure S13C-D** show accuracy after retraining on the combined MH & CIW training set.

**Figure S13**

| A)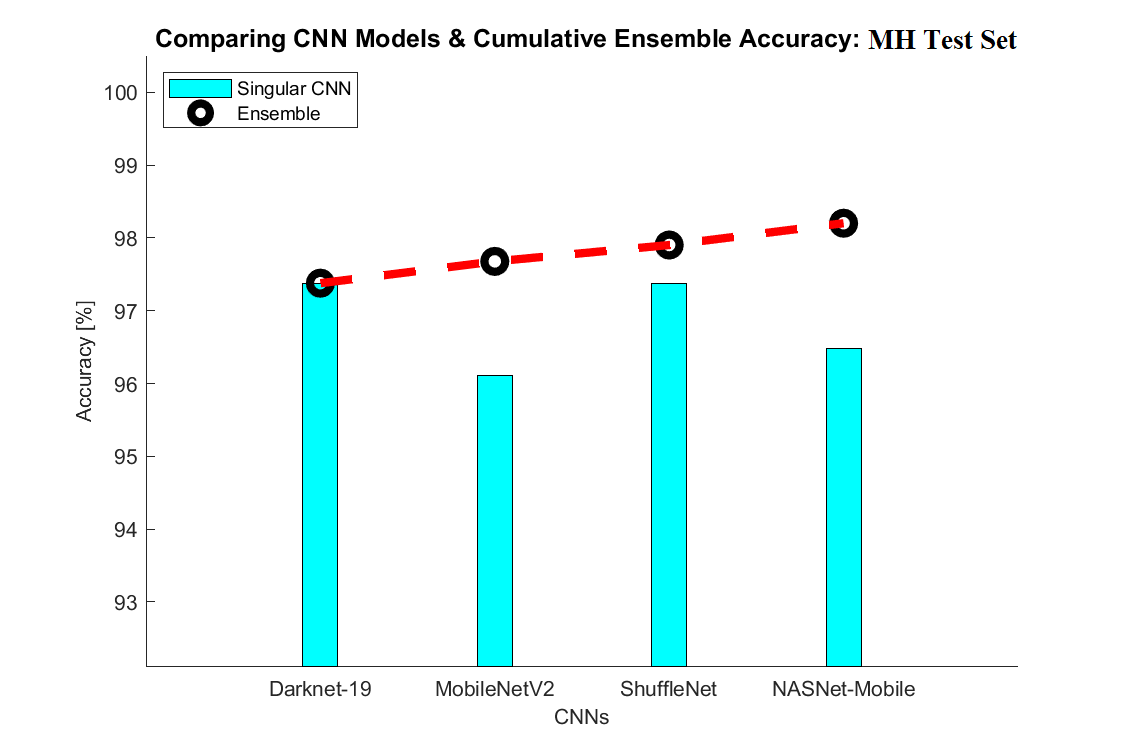 | B)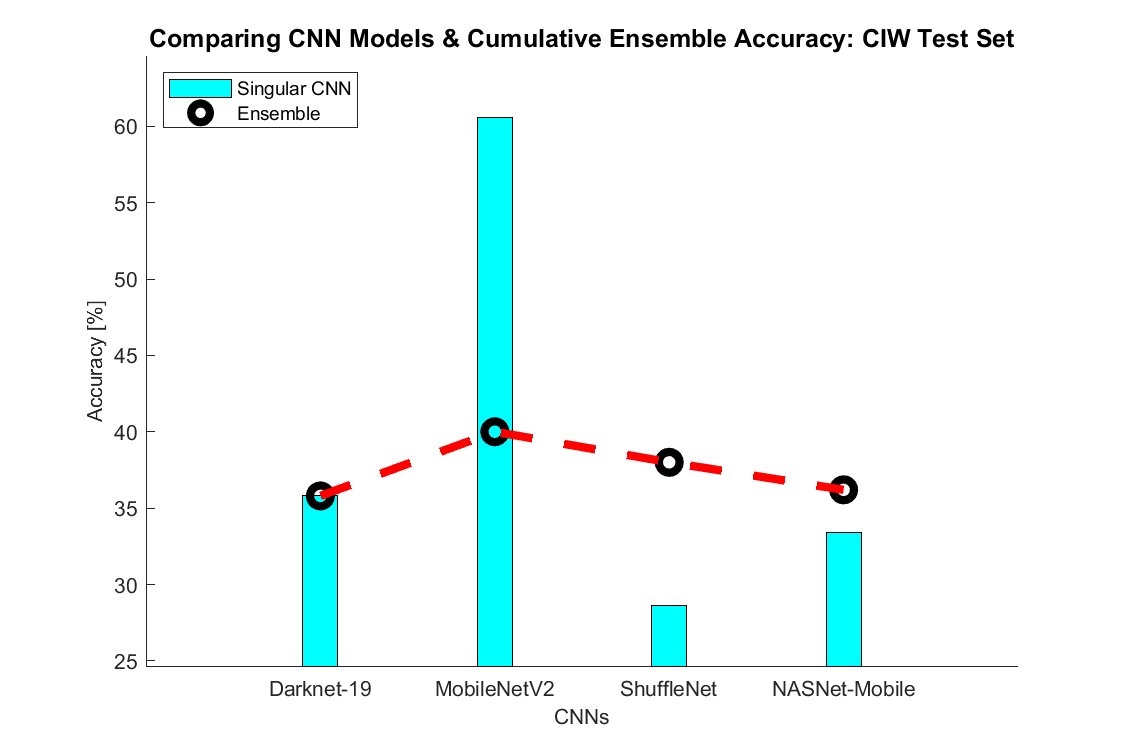 |
| --- | --- |
| C)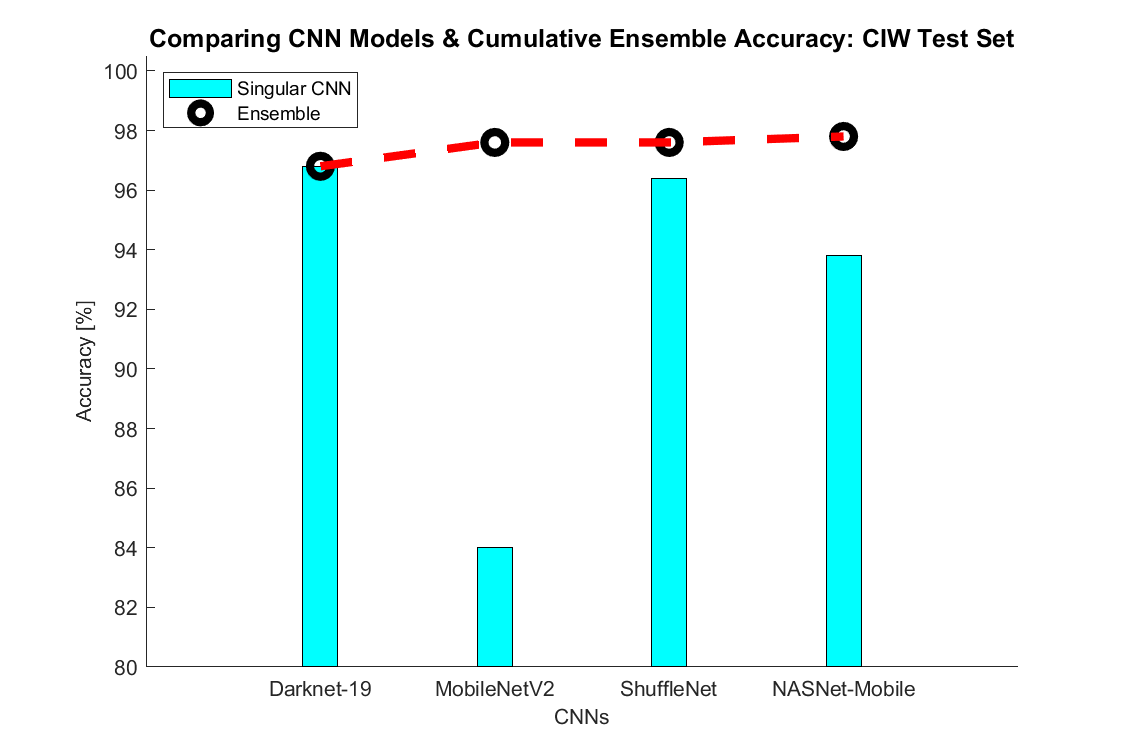 | D)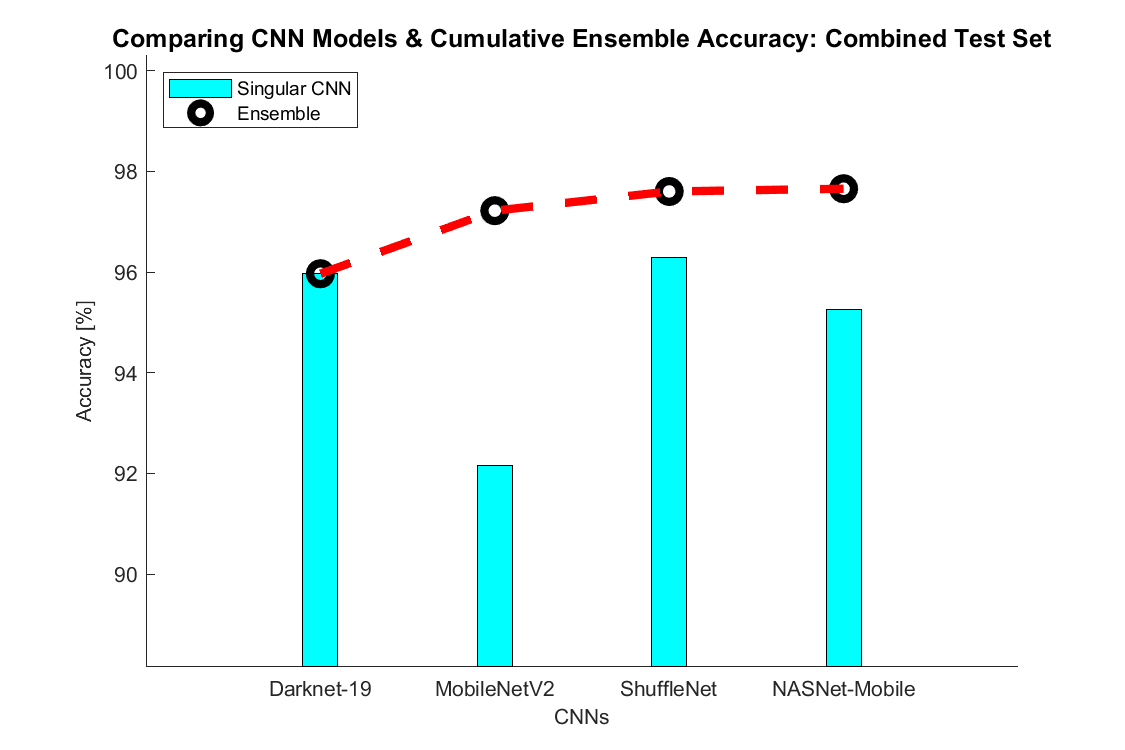 |

**Figure S13:** The graphs above show the accuracy of the individual CNNs and the cumulative ensemble accuracy when tested against the MH (A), CIW (B&C), and combined test (D) sets. A and B illustrate model accuracy after training on the MH training set, while C and D illustrate model accuracy after retraining on the combined MH & CIW training set.

# **Dataset Comparison by Morphology**

**Figure S14**


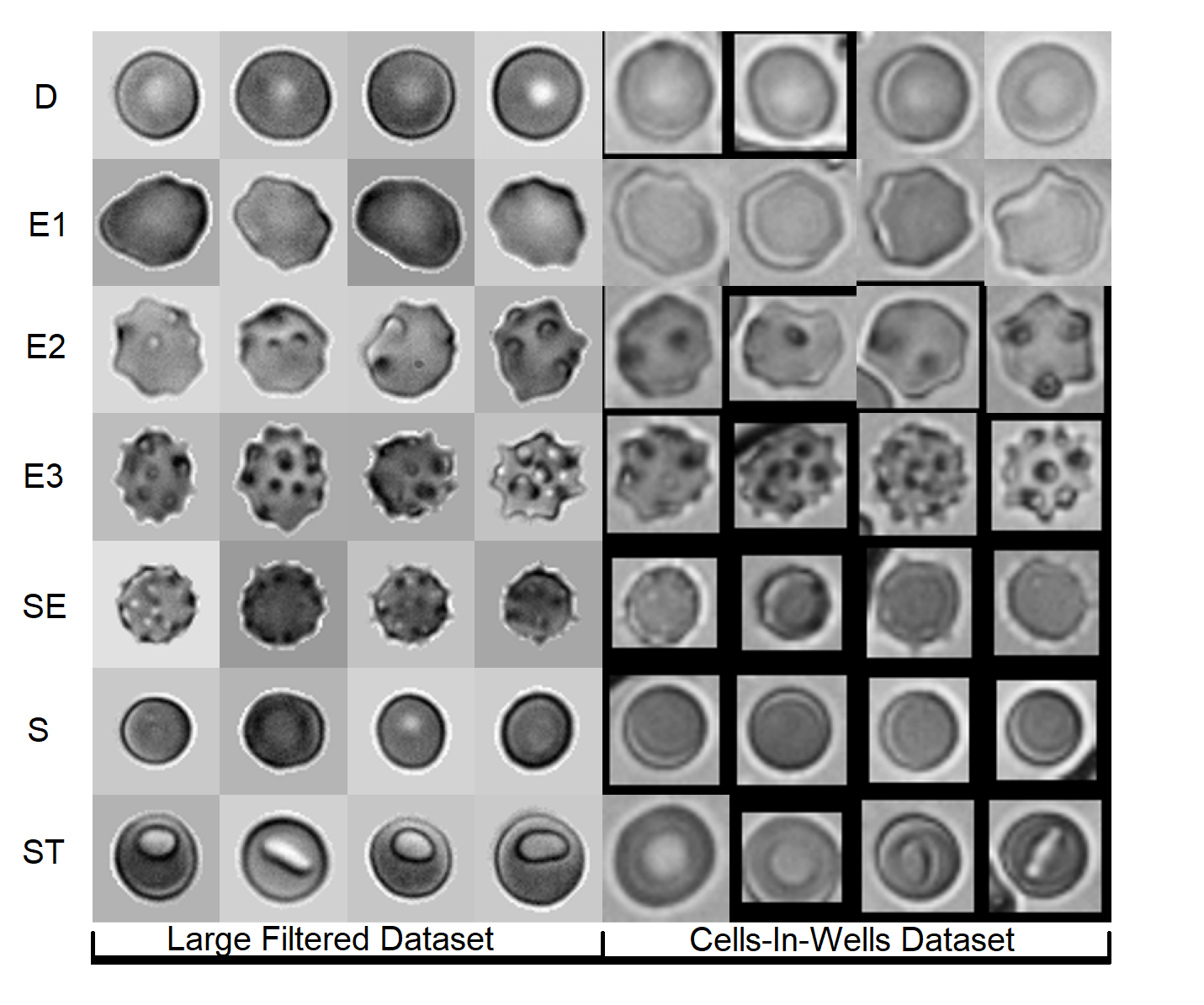


**Figure S14:** Many features differ between the MH (left columns) and CIW (right columns) training sets, including image dimensions, zoom, focus, and background.

# **The Cells in Wells Dataset: Well Video Tracking Samples**

**Figures S15**, **S16**, and **S17** demonstrate the tracking capabilities of our framework as RBCs within microfluidic wells undergo washing. Note that classification is the result of a 100-frame moving average, so a delay is present as cells change morphologies. Videos are divided into 4 quadrants. The top left shows the entire segmented video with a red box around the well of interest. The top right depicts the well of interest with anthropomorphically named and morphology labeled RBCs. The bottom left displays cropped RBC images with morphology labels and their associated confidence scores. The bottom right is the donor, wash, well, and frame count information.

**Figure S15**


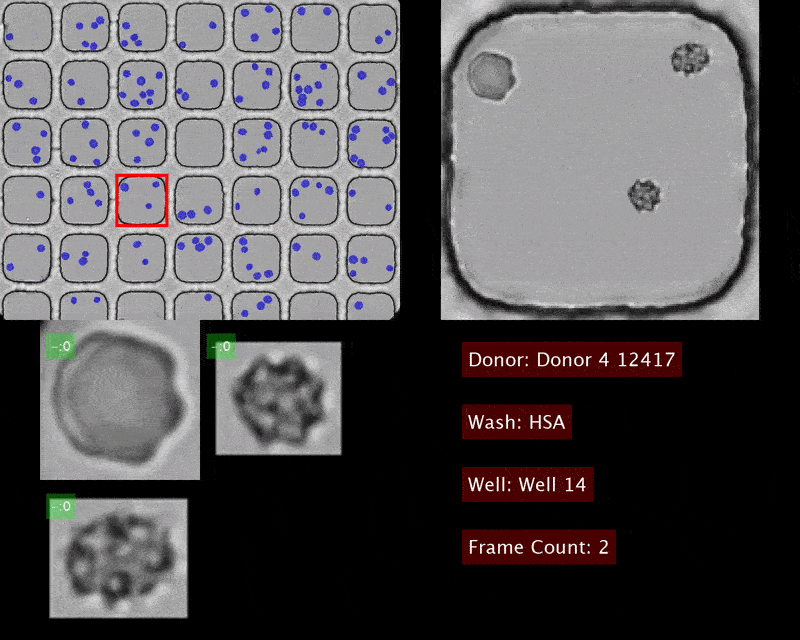


**Figure S15:** A video of Donor 4’s well 14 as it undergoes HSA washing. Note that RBC “Jeff” changes from SE to E1 by frame 600.

**Figure S16**


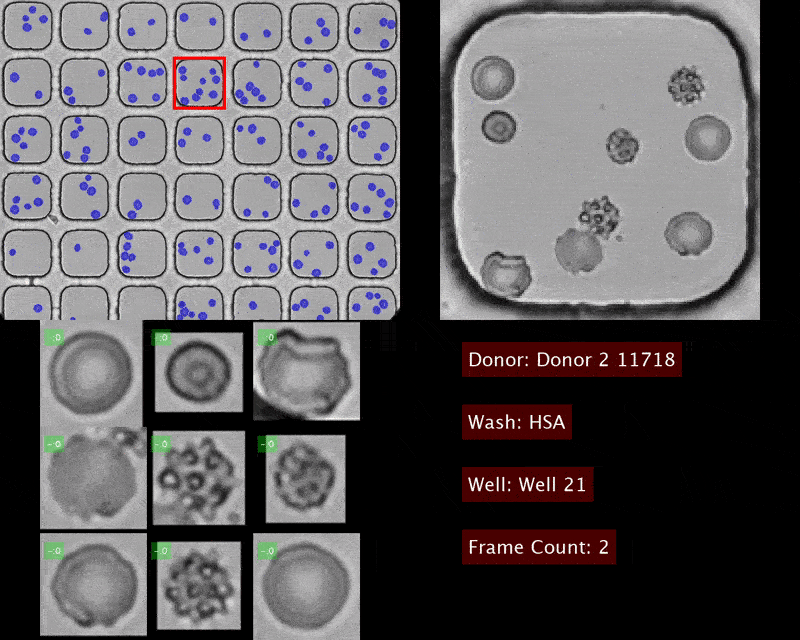


**Figure S16:** A video of Donor 2’s well 21 as it undergoes HSA washing. This video shows a crowded well with ghost RBCs appearing and disappearing from the tracking list. Note that RBC “Rin” changes from SE to E1 quickly, but the moving average delay does not immediately reflect this change.

**Figure S17**


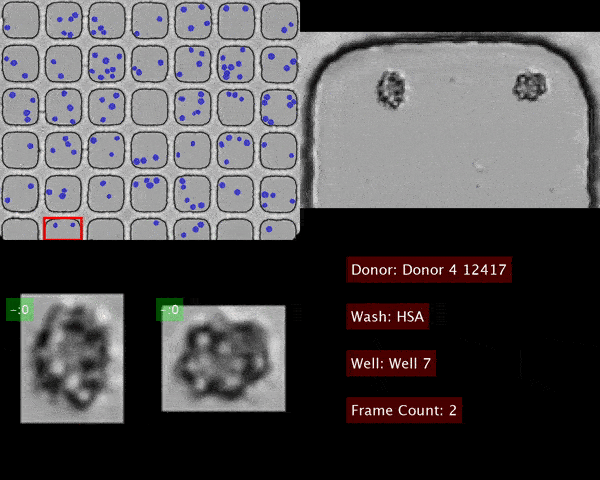


**Figure S17:** A video of Donor 4’s well 7 as it undergoes HSA washing. This video shows RBC “Yuno” changing from SE to E1.
